# Supplementary material for: Health and well-being issues of Nepalese migrant workers in the Gulf Cooperation Council countries and Malaysia: a systematic review
Source: BMJ Open. 2020 Oct 26;10(10):e038439. doi: 10.1136/bmjopen-2020-038439 (PMC7592279; doi:10.1136/bmjopen-2020-038439)
Supplement: Supplementary data [file bmjopen-2020-038439supp001.pdf]

Appendix 1 Keywords used for search in MEDLINE

1. Migration {Including Related Terms}
2. Migrant {Including Related Terms}
3. Emigrant {Including Related Terms}
4. Immigrant{Including Related Terms}
5. Expatriate {Including Related Terms}
6. Foreign worker {Including Related Terms}
7. Labor migration {Including Related Terms}
8. Left-behind {Including Related Terms}
9. Migrant families {Including Related Terms}
10. Or/1-9
11. Nepal {Including Related Terms}
12. Nepalese {Including Related Terms}
13. Nepali {Including Related Terms}
14. UAE or United Araba Emirates {Including Related Terms}
15. GCC or Gulf Cooperating council {Including Related Terms}
16. Middle East {Including Related Terms}
17. Bahrain {Including Related Terms}
18. Saudi Arabia {Including Related Terms}
19. Oman {Including Related Terms}
20. Qatar {Including Related Terms}
21. Kuwait {Including Related Terms}
22. Malaysia {Including Related Terms}
23. Or/11-22
24. 10 AND 23
