# Supplementary material for: Health and well-being issues of Nepalese migrant workers in the Gulf Cooperation Council countries and Malaysia: a systematic review
Source: BMJ Open. 2020 Oct 26;10(10):e038439. doi: 10.1136/bmjopen-2020-038439 (PMC7592279; doi:10.1136/bmjopen-2020-038439)
Supplement: Supplementary data [file bmjopen-2020-038439supp002.pdf]

Appendix 2:

Quality Assessment of Quantitative Studies (Prevalence Surveys)

Reference	Was the sample frame appropriate to address the target population?	Were study participants sampled in an appropriate way?	Was the sample size adequate?	Were the study subjects and the setting described in detail?	Was the data analysis conducted with sufficient cover age of the identified sample?	Were valid methods used for the identification of the condition?	Was the condition measured in a standard, reliable way for all participants?	Was there appropriate statistical analysis?	Was the response rate adequate, and if not, was the low response rate managed appropriately?	Scores	Overall quality
Overview N-28 studies	Yes - 27 (96.5%)	Yes - 20 (71.4%)	Yes - 17 (60.7%)	Yes – 25 (89.3%)	Yes – 26 (92.9%)	Yes – 26 (92.9%)	Yes – 25 (89.3%)	Yes – 25 (89.3%)	Yes – 15 (53.6%)		H- 15 (53.6%)
	Unclear - 1 (3.5%)	Unclear- 7 (25.0%)	Unclear– 11 (39.3%)	Unclear – 3 (10.7%)	Unclear – 2 (7.1%)	Unclear – 2 (7.1%)	Unclear – 3 (10.7%)	Unclear – 3 (10.7%)	Unclear – 13 (46.4%)		M- 13 (46.4%)
	No - 0	No - 1 (3.6%)	No - 0	No – 0	No - 0	No - 0	No - 0	No - 0	No - 0		
Abu-Madi et al, 2016a	Yes	Yes	Yes	Yes	Yes	Yes	Yes	Yes	Yes	9	High
Abu-Madi et al, 2016b	Yes	Yes	Yes	Unclear	Yes	Yes	Yes	Unclear	Yes	7	Moderate
Abu-Madi et al, 2011	Yes	Yes	Unclear	Yes	Yes	Yes	Yes	Yes	Yes	8	High
Al-Awadhi et al, 2019	Yes	Yes	Yes	Yes	Yes	Yes	Yes	Yes	Unclear	8	High
Al-Marri et al, 2001	Yes	Yes	Yes	Yes	Yes	Yes	Yes	Unclear	Yes	8	High
Alswaidi et al, 2013	Yes	Yes	Yes	Yes	Yes	Yes	Yes	Yes	Yes	9	High
Al-Thani et al, 2015	Yes	Unclear	Yes	Yes	Yes	Yes	Unclear	Yes	Yes	7	Moderate
Chan et al, 2008	Unclear	Yes	Yes	Yes	Yes	Yes	Yes	Yes	Yes	8	High
Chattu et al, 2013	Yes	Yes	Unclear	Yes	Unclear	Yes	Unclear	Yes	Unclear	5	Moderate
Dafalla et al, 2017	Yes	Unclear	Yes	Yes	Yes	Yes	Yes	Yes	Unclear	7	Moderate
Dhakal et al, 2020	Yes	Yes	Unclear	Yes	Yes	Unclear	Yes	Yes	Unclear	6	Moderate
Humphery et al, 2016	Yes	Unclear	Unclear	Yes	Yes	Yes	Yes	Yes	Unclear	6	Moderate
Ibrahim et al, 2009	Yes	Yes	Unclear	Yes	Unclear	Yes	Yes	Yes	Unclear	6	Moderate
Imam et al, 2015	Yes	Yes	Unclear	Yes	Yes	Yes	Yes	Yes	Yes	8	High
Irfan et al, 2016	Yes	Yes	Yes	Yes	Yes	Yes	Yes	Yes	Unclear	8	High

Reference	Was the sample frame appropriate to address the target population?	Were study participants sampled in an appropriate way?	Was the sample size adequate?	Were the study subjects and the setting described in detail?	Was the data analysis conducted with sufficient cover age of the identified sample?	Were valid methods used for the identification of the condition?	Was the condition measured in a standard, reliable way for all participants?	Was there appropriate statistical analysis?	Was the response rate adequate, and if not, was the low response rate managed appropriately?	Scores	Overall quality
Joshi et al, 2011	Yes	Yes	Yes	Yes	Yes	Yes	Yes	Yes	Yes	9	High
Joshi et al, 2014	Yes	No	Yes	Yes	Yes	Unclear	Yes	Unclear	Yes	7	Moderate
Kavarodi et al, 2014	Yes	Yes	Yes	Yes	Yes	Yes	Yes	Yes	Yes	9	High
Latifi et al, 2015	Yes	Yes	Yes	Unclear	Yes	Yes	Yes	Yes	Yes	8	High
Min, 2016	Yes	Yes	Unclear	Yes	Yes	Yes	Yes	Yes	Yes	8	High
Noordin et al, 2017	Yes	Yes	Unclear	Unclear	Yes	Yes	Yes	No	Unclear	5	Moderate
Pradhan et al, 2019	Yes	Unclear	Unclear	Yes	Yes	Yes	Unclear	Yes	Unclear	5	Moderate
Sahimin et al, 2019	Yes	Yes	Yes	Yes	Yes	Yes	Yes	Yes	Unclear	8	High
Sahimin et al, 2018	Yes	Yes	Yes	Yes	Yes	Yes	Yes	Yes	Yes	9	High
Sahimin et al, 2017	Yes	Yes	Yes	Yes	Yes	Yes	Yes	Yes	Yes	9	High
Simkhada et al, 2017	Yes	Unclear	Unclear	Yes	Yes	Yes	Yes	Yes	Unclear	6	Moderate
Woh et al, 2016	Yes	Unclear	Unclear	Yes	Yes	Yes	Yes	Yes	Unclear	6	Moderate
Woh et al, 2017	Yes	Unclear	Unclear	Yes	Yes	Yes	Yes	Yes	Yes	7	Moderate

Quality Assessment of Quantitative Studies (Analytical Cross-sectional Surveys)

Reference (n=3 studies)	Were the criteria for inclusion in the sample clearly defined?	Were the study subjects and the setting described in detail?	Was the exposure measured in a valid and reliable way?	Were objective, standard criteria used for measurement of the condition?	Were confounding factors identified?	Were strategies to deal with confounding factors stated?	Were the outcomes measured in a valid and reliable way?	Was appropriate statistical analysis used?	Score	Overall Quality
Adhikary et al, 2017	Yes	Yes	Yes	Yes	No	No	Yes	Yes	6	Moderate
Adhikary et al, 2018	Yes	Yes	Yes	Yes	No	No	Yes	Yes	6	Moderate
Khaled and Gray, 2019	Yes	Yes	Yes	Yes	No	No	Yes	Yes	6	Moderate

Quality Assessment of the Qualitative Studies

Reference (n=2 studies)	Is there congruity between the stated philosophical perspective and the research methodology?	Is there congruity between the research methodology and the research question or objectives?	Is there congruity between the research methodology and the methods used to collect data?	Is there congruity between the research methodology and the representation and analysis of data?	Is there congruity between the research methodology and the interpretation of results?	Is there a statement locating the researcher culturally or theoretically?	Is the influence of the researcher on the research, and vice- versa, addressed?	Are participants, and their voices, adequately represented?	Is the research ethical according to current criteria or, for recent studies, and is there evidence of ethical approval by an appropriate body?	Do the conclusions drawn in the research report flow from the analysis, or interpretation, of the data?	Score	Overall Quality
Adhikary et al, 2019	Yes	Yes	Yes	Yes	Yes	Unclear	Unclear	Yes	Yes	Yes	8	High
Regmi et al, 2019	Yes	Yes	Yes	Unclear	Yes	No	Unclear	Yes	Yes	Yes	7	Moderate
