# Supplementary material for: Health and well-being issues of Nepalese migrant workers in the Gulf Cooperation Council countries and Malaysia: a systematic review
Source: BMJ Open. 2020 Oct 26;10(10):e038439. doi: 10.1136/bmjopen-2020-038439 (PMC7592279; doi:10.1136/bmjopen-2020-038439)
Supplement: Author's manuscript [file bmjopen-2020-038439.draft_revisions.pdf]

BMJ Open

BMJ Open is committed to open peer review. As part of this commitment we make the peer review history of every article we publish publicly available.

When an article is published we post the peer reviewers' comments and the authors' responses online. We also post the versions of the paper that were used during peer review. These are the versions that the peer review comments apply to.

The versions of the paper that follow are the versions that were submitted during the peer review process. They are not the versions of record or the final published versions. They should not be cited or distributed as the published version of this manuscript.

BMJ Open is an open access journal and the full, final, typeset and author-corrected version of record of the manuscript is available on our site with no access controls, subscription charges or pay-per-view fees (http://bmjopen.bmj.com).

If you have any questions on BMJ Open's open peer review process please email info.bmjopen@bmj.com

BMJ Open

Health and Wellbeing Issues of Nepalese Migrant Workers in the Gulf Cooperation Council Countries and Malaysia: A Systematic Review

Journal:	*BMJ Open*
Manuscript ID	bmjopen-2020-038439
Article Type:	Original research
Date Submitted by the Author:	10-Mar-2020
Complete List of Authors:	Paudyal, Priyamvada; Brighton and Sussex Medical School, Primary Care and Public Health<br>Kulasabanathan, Kavian ; Brighton and Sussex Medical School, Primary Care and Public Health<br>Cassell, Jackie; Brighton and Sussex Medical School, Department of Sexually Transmitted Diseases<br>Memon, Anjum; Brighton and Sussex Medical School Division of Primary Care and Public Health Medicine, ; Brighton and Sussex Medical School Division of Primary Care and Public Health Medicine,<br>Simkhada, Padam; University of Huddersfield, School of Human and Health Sciences<br>Wasti, Sharada; Green Tara Nepal
Keywords:	PUBLIC HEALTH, EPIDEMIOLOGY, Health & safety < HEALTH SERVICES ADMINISTRATION & MANAGEMENT

SCHOLARONE™
Manuscripts

I, the Submitting Author has the right to grant and does grant on behalf of all authors of the Work (as defined in the below author licence), an exclusive licence and/or a non-exclusive licence for contributions from authors who are: i) UK Crown employees; ii) where BMJ has agreed a CC-BY licence shall apply, and/or iii) in accordance with the terms applicable for US Federal Government officers or employees acting as part of their official duties; on a worldwide, perpetual, irrevocable, royalty-free basis to BMJ Publishing Group Ltd ("BMJ") its licensees and where the relevant Journal is co-owned by BMJ to the co-owners of the Journal, to publish the Work in this journal and any other BMJ products and to exploit all rights, as set out in our licence.

The Submitting Author accepts and understands that any supply made under these terms is made by BMJ to the Submitting Author unless you are acting as an employee on behalf of your employer or a postgraduate student of an affiliated institution which is paying any applicable article publishing charge ("APC") for Open Access articles. Where the Submitting Author wishes to make the Work available on an Open Access basis (and intends to pay the relevant APC), the terms of reuse of such Open Access shall be governed by a Creative Commons licence – details of these licences and which Creative Commons licence will apply to this Work are set out in our licence referred to above.

Other than as permitted in any relevant BMJ Author's Self Archiving Policies, I confirm this Work has not been accepted for publication elsewhere, is not being considered for publication elsewhere and does not duplicate material already published. I confirm all authors consent to publication of this Work and authorise the granting of this licence.

For peer review only

Health and Wellbeing Issues of Nepalese Migrant Workers in the Gulf Cooperation Council Countries and Malaysia: A Systematic Review

Priyamvada Paudyal[1], Kavian Kulasabanathan[1], Jackie Cassell[1], Anjum Memon[1], Padam Simkhada[2], Sharada Pd. Wasti[3]

[1]Department of Primary Care and Public Health, Brighton and Sussex Medical School, Brighton UK; [2]Faculty of Health, University of Huddersfield, UK; [3]Green Tara Nepal, Kathmandu, Nepal

Corresponding Author: Dr Priyamvada Paudyal
Department of Primary Care and Public Health, Brighton and Sussex Medical School, Room 322, Watson Building, Village Way, Falmer, BRIGHTON, BN1 9PH, UK
+44 (0) 1273 644548; p.paudyal@bsms.ac.uk

Running Title: Health issues of Nepalese migrant workers in Gulf Countries and Malaysia

Word Count: 3439

For peer review only

Abstract:

Objective: Approximately 3.8 million Nepalese nationals (14% of the total population) work abroad. This systematic review aimed to summarise the evidence on health and wellbeing of Nepalese migrant workers in Gulf Cooperation Council (GCC) countries and Malaysia, the destination countries for 85% of Nepalese migrant workers.

Data sources: A combination of migration and country specific search terms were used to identify relevant studies using EMBASE and MEDLINE databases.

Eligibility Criteria: Studies were eligible if they: 1) included Nepalese migrant workers aged ⩾18 years working currently in the GCC countries or Malaysia or returnee migrant workers from these countries; 2) were primary studies that investigated health and wellbeing status/issues; and 3) were published in English language before 20 May 2019.

Data Synthesis: Narrative summary of the data was provided along with the data from the individual studies presented in the summary table.

Quality Assessment: All the included studies were assessed using the Joanna Briggs Institute Checklist for Prevalence Studies.

Results: A total of 23 studies were eligible for inclusion; nine studies were conducted in Qatar, six in Malaysia, five in Nepal, two in Saudi Arabia and one in UAE. The majority of the studies (n=15) scored as 'high' quality and all others (n=8) as 'moderate' quality. Five key health and wellbeing related issues were identified in this population: a) occupational hazards; b) sexual health; c) mental health; d) healthcare access and e) infectious diseases.

Conclusion: To our knowledge, this is the most comprehensive review of the health and well-being of Nepalese migrant workers in the GCC countries and Malaysia. This review highlights an urgent need to identify and implement policies and practices across Nepal and destination countries to protect the health and wellbeing of migrant workers.

Protocol Registration: The review protocol can be found at http://sro.sussex.ac.uk/id/eprint/86400/.

Key Terms: Migrants, GCC countries, Health, Wellbeing, Nepalese

For peer review only

Strengths and limitation of this study

- To our knowledge, this is the most comprehensive review on health and wellbeing issues of Nepalese migrant workers in GCC and Malaysia.
- Screening of studies and quality assessment was conducted by two independent reviewers, ensuring the methodological robustness of the review.
- The review did not systematically include grey literature although a number of key reports were used as reference points to compare to our findings from the peer-reviewed literature.

For peer review only

For peer review only - http://bmjopen.bmj.com/site/about/guidelines.xhtml

Introduction

Migration is the overarching narrative of our time, and its impact is increasingly being recognised in global public health agendas. The United Nations (UN) Sustainable Development Goals (SDGs) identify migration as a catalyst for development and recommend that 'no-one should be left behind' to achieve Universal Health Coverage for all[1]. According to the World Migration Report 2020, the number of international migrants has reached approximately 272 million, and two third of these are estimated to be labour migrants[2]. Labour migration has been a key determinant of population changes in Asia, especially in Gulf Cooperation Council (GCC) countries, a major destination for workers within Asia[2].

Nepal is a low-income country going through a demographic transition, with an ageing population and attendant chronic diseases. Approximately 3.8 million Nepalese (14% of the total population) work abroad (not counting those working in India)[3]. The Nepal Demographic and Health Survey (2016) reported that nearly half (47%) the households have at least one family member who migrated in the last 10 years either in internal or international destinations[4]. These migrant workers contribute over a quarter (26.9%) of the country's gross domestic product (GDP) through remittance from abroad[5]. The most recent Nepalese Government report shows that the migration outflow consists predominantly of low-skilled male workers, primarily to Malaysia and the GCC countries[5].

Labour migration contributes significantly to the sociocultural and economic development of both origin and destination countries. However, migrant workers experience specific vulnerabilities, and face a range of health risks while working abroad. These risks are particularly significant for Nepalese workers in the GCC countries, as they are often employed in occupations considered 'difficult, dirty, and dangerous (3Ds)'. These are sectors with higher occupational risks such as agriculture, construction, transport and heavy industry. Furthermore, Nepalese migrant workers consistently work for longer hours as compared to native workers[6 7] and are often exposed to factors which promote poor health and wellbeing, including low wages, poor housing, an unhealthy diet, and difficulty in accessing health services[6, 8]. Many Nepalese migrant workers die abroad every year including a significant number that are unexplained, while a large number return home with debilitating injuries, and both mental and physical illness[6]. This systematic review identified and summarised the evidence from primary studies on the health and wellbeing of Nepalese migrant workers in the GCC countries and Malaysia, the destination countries for 85% of labour migration. This review was conducted as a part of University of Sussex internally funded Global Challenges Research Fund (GCRF)

For peer review only - http://bmjopen.bmj.com/site/about/guidelines.xhtml

project to develop a culturally relevant intervention to support the health and wellbeing of Nepalese migrant workers in GCC countries.

Methods

Protocol Registration

This study protocol was registered at the University of Sussex (http://sro.sussex.ac.uk/id/eprint/86400/). The study followed the PRISMA (Preferred Reporting Items for Systematic Reviews and Meta-Analyses) guidelines and recommendations of the Cochrane Collaboration (www.prisma-statement.org).

Electronic Search

A combination of migration specific search terms (migration, migrant, emigrant, immigrant, expatriate, foreign worker, labor migration, left-behind, migrant families) and country specific search terms (Nepal, Nepalese, Nepali, UAE, United Arab Emirates, GCC, Gulf Cooperation Council, Middle East, Bahrain, Saudi Arabia, Oman, Qatar, Kuwait, Malaysia) were used to identify relevant studies using EMBASE and MEDLINE databases (Appendix 1). The search aimed to identify all relevant studies regardless of any health outcomes used. As such, no health outcome specific terms were used to limit the electronic search. Reference lists of the relevant studies including those of related systematic reviews and reference lists of the selected studies were further screened to identify potentially eligible studies.

Inclusion and Exclusion Criteria

Studies were eligible if they: 1) included Nepalese migrant workers aged ⩾18 years working currently in the GCC countries or Malaysia or returnee migrant workers from these countries; 2) provided primary data on health and wellbeing status/issues (physical health, mental health, accidents and injuries); and 3) were published in English language before 20 May 2019.

Article Screening and Selection

Once the electronic search was completed, the identified articles were exported to Rayyan (https://rayyan.qcri.org/welcome) and screening was carried out by two reviewers (SW and KK) independently to identify eligible articles. The titles of the identified studies were screened to remove any duplicates and irrelevant articles. The abstract of all remaining articles was screened to identify eligible full text articles. Full text articles were reviewed and a consensus

For peer review only - http://bmjopen.bmj.com/site/about/guidelines.xhtml

was reached to finalise the articles for inclusion. If more than one study were published using the same data source (e.g. routine healthcare date), we used the study with the largest sample size. Any disagreement over eligibility of studies was resolved through discussion with the third reviewer (PP).

Data Extraction and Synthesis

The information extracted from each article included: study reference (authors, publication year and country), study design and settings, participants' characteristics (sample size, age, and gender), health outcomes and key findings (Table 1). Extracted data were analysed and a summary of the narrative synthesis is reported in the results section.

Quality Assessment

Study quality assessment was done using the Joanna Briggs Institute (JBI) Critical Appraisal Tools 'Checklist for Prevalence Studies' (available at https://joannabriggs.org/research/critical-appraisal-tools.html). The assessment was undertaken independently by two reviewers (SW and KK) with any discrepancies resolved by a third reviewer (PP). As the number of studies in this population is limited, we did not exclude studies based on quality assessment. Studies were scored based on the total number of 'Yes' responses on the checklist. As in previous review[9], studies with eight or more 'Yes' response were rated as 'high' quality, four to seven as 'moderate' and three or below as 'low' quality. The results of the quality assessment are presented in Appendix 2.

Results

Screening Results

Database searches yielded 1325 articles, of these, 1183 were excluded at the title screening stage. Abstracts of the remaining 127 publications were further screened and 56 of these were excluded. Full text screening of the remaining 71 papers were carried out and a further 50 papers were excluded for various reasons (Figure 1). Altogether, 23 papers were included in this review; 21 were identified from the systematic data search and an additional two from the hand search (Figure 1).

For peer review only - http://bmjopen.bmj.com/site/about/guidelines.xhtml

Figure 1: PRISMA Flow Diagram of Study Selection

For peer review only

For peer review only - http://bmjopen.bmj.com/site/about/guidelines.xhtml

Study Characteristics

A total of 23 papers were included in the review among them nine studies were conducted in Qatar[10-18], six in Malaysia[19-24], five in Nepal[6 8 25-27], two in Saudi Arabia[28 29,] and one in UAE[30]. One study included all GCC countries and Malaysia[8], whilst another included disaggregated data for the 'Gulf states' but did not specify particular name of the country[14] (Table 1). The study design varied across the studies; the review included 10 retrospective analysis of routine healthcare data[8 10-13 16 18 20 28 29], 11 cross-sectional studies[6 17 19 21-27 30] and two prospective observational studies[14 15]. Five studies focused specifically on Nepalese migrants as their primary study population[6 8 25-27] whilst the remaining 18 studies mentioned Nepalese migrant workers as part of a sub-analysis (Table 1). The study mainly fell into two categories: those exploring the health risks and experiences of migrants while abroad and those focusing on infectious diseases (mostly done as a part of arrival screening).

Studies exploring health risk and experiences

Occupational Health and Hazards

Four studies specifically assessed occupational morbidity, mortality, and fitness to work in the destination countries[13 18 25 28]. Adhikari et al; (2017) reported that around one-fifth (17%) of migrant workers had experienced work related accidents[25]. Poor working environment and not being registered with a doctor was associated with a greater perceived health risk at the work place. Another study reported that over a quarter (27.9%) of migrant workers had experienced occupational injuries: more than half (52%) of these workers fell from a height, 21% had injuries due to fall of a heavy object, 17% had motor vehicle accident injuries, 5% had machinery injuries and remaining 5% had other work related injuries[13]. In a study conducted in Saudi Arabia, Nepalese migrant workers were the third-most unfit population to work; 1.6% were unfit due to the presence of infectious disease and 5.3% due to non-communicable disease[28]. Another study reported that more than one quarter (25.4%) of migrants had traffic related pedestrian injuries during abroad work[18] (Table 1).

Sexual Health

Only one study in this review assessed the knowledge, attitudes and perceptions (KAP) of HIV/AIDS related risks[27.] The study was conducted among 408 adult Nepalese migrants with at least six months of work experience in one of the three Gulf countries (Qatar, Saudi Arabia and UAE). The study showed that 91% of respondents had concerns about HIV/AIDS, and 17.2% of workers reported having sexual intercourse with a partner other than their spouse

For peer review only - http://bmjopen.bmj.com/site/about/guidelines.xhtml

within the last 12 months. More than half (59%) of the respondents perceived themselves at high risk of being infected due to their sexual activities[27] (Table 1).

Mental Health

Two studies examined mental health issues among migrant workers. One study on Nepalese female returnee migrant workers from Middle East and Malaysia reported the prevalence of mental health problems as 8.3%[8]. Another study reported that almost a quarter (23%) of labour migrants to Malaysia, Qatar and Saudi Arabia had experienced mental health issues, with a strong positive link between perceived health risk in the work environment and mental health status[26] (Table 1).

Healthcare Access

Three studies focused on labour migrants' healthcare access issues[6 8 25]. Adhikari et al; (2017) reported that workers who were not registered with a doctor had poor health outcomes compared to those who were registered[25]. Another study also reported that only 36.5% workers had access to health insurance and about half (48.7%) did not have paid sick leave during their health problems[6]. Another study on Nepalese female returnee migrant workers reported that only 11% of respondents received health services during their abroad work[8] (Table 1).

Other Health Issues

One study on Nepalese female returnee migrant workers reported a prevalence of workplace abuse, torture or maltreatment at the workplace, and physical harm at 41%, 31% and 11%, respectively[8]. Another study reported a higher proportion of traffic related pedestrian injuries among Nepalese migrant workers in Qatar (24%) despite only 16% of the population of Qatar being Nepalese[18]. Clinical prevalence of oral lesions among migrant workers was found to be 4.6%[17] (Table 1).

Studies on Infectious Diseases (Parasitic and Bacterial Infections, TB and Hepatitis E)

Of the 23 included studies, 14 studies reported the proportion of sero-and/or faeco positive cases of infectious diseases (parasitic and bacterial Gastroenteric infections, tuberculosis, hepatitis E) [10-12 14 15 19 21-24 29-31]. In several of these studies, Nepalese migrant workers had the higher proportion of infectious disease cases among the population studied. These infectious diseases included, toxoplasmosis (46.2%, working in Malaysia)[19], tuberculosis (7%, Saudi Arabia)[29], diarrhoeal bacterial infection (26.6%, Qatar)[14], protozoan ova/cysts (13.7%),

For peer review only - http://bmjopen.bmj.com/site/about/guidelines.xhtml

helminths (6.2%), and hookworms (4.3%, Qatar)[10], Hepatitis E (74%, Qatar)[15], Brugian Lymphatic Filariasis (BmR1) (2.9%, Malaysia), parasitic infection (BmSXP) (13%, Malaysia)[21], and intestinal parasites (21.4%, Malaysia)[23] (Table 1).

Quality Assessment

All the included studies were assessed the using the Joanna Briggs Institute Checklist for Prevalence Studies, and majority of the studies (65% n=15/23) scored as 'high' quality and remaining all (35%, n=8/27) found as a 'moderate' quality[8 10 13-15 21 24 29]. None of the studies were rated as poor quality. The results of the quality assessment are presented in Appendix 2.

Discussion

To our knowledge, this is the most comprehensive review of the health and well-being status/issues of the Nepalese migrant workers in the GCC countries and Malaysia. The resultant lack of disaggregated demographic data means that the overall characteristics of Nepalese participants is difficult to determine. The dissonance between issues covered in the peer-reviewed and grey literature for this population, namely in national and international media and in government reports, is notable. Disproportionately few studies focused on occupational and mental health of migrant workers.

Occupational Health

Our review identified only four papers focusing on occupational morbidity, mortality and fitness to work in the destination countries[13 18 25 28]. Only one of these focused solely on Nepalese migrants, and none compared occupation or working conditions with morbidity and mortality experienced[25]. This a crucial gap in the literature and further studies are needed to guide policy change. There has been widespread media coverage of the poor working conditions faced by Nepalese migrant workers and health impacts of these conditions are highlighted by the plight of manual labourers working for the forthcoming 2022 FIFA Qatar World Cup. Close to a fifth of labour migrants to Malaysia, Qatar and Saudi Arabia had experienced a workplace accident[25]. According to a Nepalese government report, there were circa 6,000 deaths among Nepalese migrant workers abroad between 2008 and 2017, and over 38% of the deaths were deemed either of natural or other/unidentified cause[5]. The magnitude of this proportion raises questions about robustness of post-mortem investigative practices and classification methodologies, a concern highlighted by both the Nepalese government and civil

For peer review only - http://bmjopen.bmj.com/site/about/guidelines.xhtml

society groups[32]. Indeed, Pradhan et al; (2019) suggest that many deaths attributed to cardiovascular diseases and 'natural causes' correlate with longer hours worked in high temperatures in this setting[33]. It is worth noting that Nepalese migrant workers themselves are not oblivious to these occupational risks- those who reported a poor or very poor work environment were found to be 3.5 times more likely to suffer a workplace accident[25].

Mental Health

Only two studies in the review reported on mental health issues. Adhikari et al; (2018) reported that almost a quarter of labour migrants to Malaysia, Qatar and Saudi Arabia had experienced mental health issues, with a strong positive correlation between perceived health risk in the work environment and mental health status[26]. Similar findings were reported in a cross-sectional study of 5000 migrant workers in Shanghai, where 21% reported mental disorders such as obsessive-compulsive disorder, anxiety, and hostility[34].

The Nepalese government report suggests that suicide is a significant cause of mortality in labour migrants to GCC countries and Malaysia, and there is evidence that mental health is an underexplored issue facing this population[35-37]. The paucity of peer-reviewed studies exploring risk factors of poor mental status and psychiatric morbidity for this population requires urgent attention.

Migration for work is a time of significant turmoil: new language, new culture and poor working conditions. Loss of protective familial and wider social networks exacerbate feelings of homesickness, loneliness and hopelessness that commonly develop amongst this population[38-40]. Psychiatric under-diagnosis is common in deprived populations and is compounded by poor screening of those with pre-existing psychiatric conditions[41-43]. The result is lack of mental health support and omission of medications in destination contexts that can worsen conditions. Most common psychiatric morbidity in this population centred around depressive and anxiety-related disorders, although the impact of addiction particularly of alcohol consumption remains underexplored[37 44-46]. The impacts of labour migration on the mental health of left-behind families is also important, but beyond the scope of this review[35 47].

Sexual Health

Only a single study in this review examined sexual health issues amongst this population and exploring HIV/AIDS knowledge, attitudes and perceptions amongst Nepalese migrant workers. Joshi et al; (2014) reported that over 17% had had sexual intercourse with someone

For peer review only - http://bmjopen.bmj.com/site/about/guidelines.xhtml

other than their spouse or partner during the final 12 months of their stay abroad[31]. This highlights higher levels of sexual risk taking behaviour, echoed by studies focusing on Nepalese migrants to India, which showed widespread use of local female sex-workers by male Nepalese migrant populations, multiple sexual partners and low levels of condom use. Whilst there may be differences between the Indian and GCC or Malaysian contexts, the authors note there is a clear dearth of evidence around non-HIV/AIDS related sexual health of these migrants, and the impact of this on left-behind families[48 49]. Similar findings also revealed from the studies in Bangladesh and China among migrant workers at high risk of heterosexual HIV acquisition[50 51].

Infectious Disease

Close to two thirds of the studies focused on migrant workers in a destination country and provided minimal disaggregated analysis on the Nepalese sub-population. Majority of these were done as a part of arrival screening and focus on infectious diseases were conducted from a destination country perspective. Overwhelmingly, the discussion sections of these studies focused on Nepalese migrant workers as potential vectors for transmitting infectious diseases to native population. This health security framing overlooks Nepalese labour migrants as a vulnerable population by virtue of their poor socioeconomic status in their origin country as well poor working and living conditions, and poor access to healthcare in destination countries[6 52 53]. Similar findings were also reported in a study from Singapore where a relatively high prevalence of malaria, hepatitis and tuberculosis was reported among migrant workers in Singapore[54]. Migrant workers in South Asia generally appear to have a greater prevalence of infectious diseases due to the complex interaction of several factors- this includes higher prevalence of infectious diseases in their native countries together with aforementioned poor access to healthcare and low socioeconomic status[7]. Acknowledgment and consequent introduction of policies to improve these structural drivers of infectious diseases amongst Nepalese migrants would be a more holistic approach that might both better protect the local population and improve the health and wellbeing of the vulnerable migrant population[55.]

Literature Gap for Female Migrant Workers

Women comprise only 7% of Nepalese labour migrant abroad[5]. However, the role of women in the migration story is far more significant and complex than this figure betrays with regards to true numbers of women migrating, roles of women 'left behind' and how it has influenced

For peer review only - http://bmjopen.bmj.com/site/about/guidelines.xhtml

gender norms in Nepalese society. All these reflect on their health from access to sexual and reproductive health services to gender-based violence[56]. We commend Simkhada et al; (2017) in their efforts to explore some of the different health issues that face female migrant workers[8]. They highlighted that almost a quarter of female Nepalese migrants faced multiple health problems and over 40% had faced workplace abuse, with close to half of the 3% that reported becoming pregnant whilst away doing so as a result of sexual abuse[8].

Female labour migration from Nepal has increased significantly over the past decade, driven by increasing demands in primarily GCC destination countries, poor agricultural employment opportunities and a slowly-changing gender norms[57]. One third of remittances to Nepal are from female migrant workers[8 58,]. 90% of female labour migrants are undocumented workers in Gulf countries and this may have resulted from the restrictive governmental labour migration policies such as prohibition of women to work in the Gulf domestic sector[59]. Precarious channels of migration bring greater risks of exploitation and harm to health[60], yet neither the peer-reviewed literature in health, nor do wider literatures reflect the magnitude of these issues. More work is required on the health of Nepalese female migrants abroad, as well the challenges in reintegration that they face on their return[57].

Strengths and Limitations

This review has several strengths. As mentioned earlier, the review is the most comprehensive review to date on this population. As GCC and Malaysia are the most attractive destinations for migration, the findings of this review will have important research and practical implications. Secondly, not restricting studies based on particular health outcomes, peer reviewed studies looking at a range of health issues in this population were included. Screening of studies and quality assessment was conducted by two independent reviewers, ensuring the methodological robustness of the review. However, there were a number of limitations. The review did not systematically include grey literature although a number of key reports were used as reference points to compare to our findings from the peer-reviewed literature. The risk of missed studies by only searching English language databases is noted, particularly through exclusion of relevant Nepalese peer-reviewed journals. Furthermore, whilst, we did not restrict the studies by its design, we did not come across any qualitative studies specifically focused within our target population.

For peer review only - http://bmjopen.bmj.com/site/about/guidelines.xhtml

Conclusion

This review identified a number of health issues among Nepalese migrant workers in the GCC countries and Malaysia, namely those centred on occupational, mental and sexual health of migrants, and infectious disease, together with health-related issues facing female labour migrants. Whilst there are early signs that Nepal may be moving beyond its predominantly remittance economy, there is no doubt that labour migration to Malaysia and the GCC countries is the reality facing an entire generation of working age Nepalese. The studies identified by the review highlight the need for improved health support, whether through regular health checks in destination countries, more stringent policies and legislation around permissible working conditions or better preparation for migration through more relevant pre-departure training. The findings suggest the urgent need to progressive policy changes, both in Nepal and destination countries, to better protect the health of labour migrants and improve their access to essential health services and acceptable working conditions.

Authors Contribution: PP and JC designed and supervised the study. PP wrote the review protocol, conducted the literature search, and wrote the final draft of the manuscript. SW and KK screened the articles, extracted the data, carried out quality assessment and contributed to the initial drafts. PP, JC and AM obtained funding for the study. JC, AM and PS reviewed and edited the manuscript. All authors read and approved the final manuscript.

Funding: This review was funded from Research England's institutional allocation from the Global Challenges Research Fund.

Conflict of Interest: None declared.

Data Availability Statement: All relevant data are provided in the manuscript or uploaded as supplementary information.

For peer review only - http://bmjopen.bmj.com/site/about/guidelines.xhtml

References

1. NPC. Sustainable development goals 2016–2030: National (preliminary) report. *Kathmandu: National Planning Commission* 2015
2. IOM. World migration report 2020: International Organization for Migration, 2019.
3. Khanal MN. Impact of male migration on contraceptive use, unmet need, and fertility in Nepal: Further analysis of the 2011 Nepal Demographic and Health Survey: Ministry of Health and Population 2013.
4. NDHS. Nepal Demographic and Health Survey 2016. Kathmandu: Ministry of Health and Population (MoHP) Nepal and ICF International Inc 2017.
5. MoLE. Labour migration for employment. A status report for Nepal: 2015/2016 – 2016/2017: Ministry of Labour and Employment Kathmandu, Nepal, 2018.
6. Joshi S, Simkhada P, Prescott GJ. Health problems of Nepalese migrants working in three Gulf countries. *BMC Int Health Hum Rights* 2011;11(3)
7. Mucci N, Traversini V, Giorgi G, et al. Migrant workers and physical health: An umbrella review. *Sustainability* 2019;11(1):232.
8. Simkhada P, Van Teijlingen E, Gurung M, et al. A survey of health problems of Nepalese female migrants workers in the Middle-East and Malaysia. *BMC Int Health Hum Rights* 2018;18(4)
9. Farsani SF, Brodovicz K, Soleymanlou N, et al. Incidence and prevalence of diabetic ketoacidosis (DKA) among adults with type 1 diabetes mellitus (T1D): a systematic literature review. *BMJ open* 2017;7(7):e016587.
10. Abu-Madi MA, Behnke JM, Boughattas S, et al. Helminth infections among long-term-residents and settled immigrants in Qatar in the decade from 2005 to 2014: temporal trends and varying prevalence among subjects from different regional origins. *Parasites & Vectors* 2016;9(1):153.
11. Abu-Madi MA, Behnke JM, Ismail A, et al. Assessing the burden of intestinal parasites affecting newly arrived immigrants in Qatar. *Parasites and Vectors* 2016;9(619)
12. Al-Marri M. Pattern of mycobacterial resistance to four anti-tuberculosis drugs in pulmonary tuberculosis patients in the state of Qatar after the implementation of DOTS and a limited expatriate screening programme. *The Int J of TB and Lung Disease* 2001;5(12):1116-21.
13. Al-Thani H, El-Menyar A, Consunji R, et al. Epidemiology of occupational injuries by nationality in Qatar: evidence for focused occupational safety programmes. *Injury* 2015;46(9):1806-13.
14. Humphrey JM, Ranbhise S, Ibrahim E, et al. Multiplex polymerase chain reaction for detection of gastrointestinal pathogens in migrant workers in Qatar. *American J of Trop Med and Hygiene* 2016;95(6):1330-37.
15. Ibrahim AS, Alkhal A, Jacob J, et al. Hepatitis E in Qatar imported by expatriate workers from Nepal: Epidemiological characteristics and clinical manifestations. *J of Med Virology* 2009;81(6):1047-51.
16. Imam YZ, Ahmedullah HS, Akhtar N, et al. Adult tuberculous meningitis in Qatar: a descriptive retrospective study from its referral center. *European Neurology* 2015;73(1-2):90-97.
17. Kavarodi AM, Thomas M, Kannampilly J. Prevalence of oral pre-malignant lesions and its risk factors in an Indian subcontinent low income migrant group in Qatar. *Asian Pac J Cancer Prev* 2014;15(10):4325-9.

For peer review only - http://bmjopen.bmj.com/site/about/guidelines.xhtml

18. Latifi R, El-Menyar A, Al-Thani H, et al. Traffic-related pedestrian injuries amongst expatriate workers in Qatar: a need for cross-cultural injury prevention programme. *Int J of Injury Control and Safety Promotion* 2015;22(2):136-42.
19. Chan B, Amal RN, Noor Hayati M, et al. Seroprevalence of toxoplasmosis among migrant workers from different Asian countries working in Malaysia. *Southeast Asian J of Trop Med and Public Health* 2008;39(1):9.
20. Min NN, Vasudevan SK, Jasman AA, et al. Work-related ocular injuries in Johor Bahru, Malaysia. *International Eye Science[Article]* 2016;16(3):416-22.
21. Noordin R, Mohd Zain SN, Yunus MH, et al. Seroprevalence of lymphatic filariasis among migrant workers in Peninsular Malaysia. *Transactions of The Royal Society of Trop Med and Hygiene* 2017;111(8):370-72.
22. Sahimin N, Lim YA, Ariffin F, et al. Socio-demographic determinants of Toxoplasma gondii seroprevalence in migrant workers of Peninsular Malaysia. *Parasites and Vectors* 2017;10(1):238.
23. Sahimin N, Douadi B, Lim ALY, et al. Distribution of Giardia duodenalis (Assemblages A and B) and Cryptosporidium parvum amongst migrant workers in Peninsular Malaysia. *Acta Tropica* 2018;182:178-84.
24. Woh PY, Thong KL, Behnke JM, et al. Characterization of nontyphoidal Salmonella isolates from asymptomatic migrant food handlers in Peninsular Malaysia. *J Food Protection* 2017;80(8):1378-83.
25. Adhikary P, Sheppard ZA, Keen S, et al. Risky work: Accidents among Nepalese migrant workers in Malaysia, Qatar and Saudi Arabia. *Health Prospect* 2017;16(2):3-10.
26. Adhikary P, Sheppard ZA, Keen S, et al. Health and well-being of Nepalese migrant workers abroad. *Int J of Migration, Health and Social Care* 2018;14(1):96-105.
27. Joshi S, Prescott GJ, Simkhada P, et al. Knowledge and risk perceptions about HIV/AIDS among Nepalese Migrants in Gulf Countries: a cross-sectional study. *Health Science Journal* 2014;8(3):350-60.
28. Alswaidi F, Memish Z, Al Hakeem R, et al. Saudi Arabian expatriate worker fitness-screening programme: a review of 14 years of data. *Eastern Medit Health J* 2013;19(7):664-70.
29. Chattu VK, Mohammad A. Tuberculosis an important global health issue in this era-a cross sectional study of epidemiology of TB among South Asian workers in Saudi Arabia. *Indian J Public Health* 2013;4:278.
30. Dafalla AIA, Almuhairi SASO, AlHosani MHJ, et al. Intestinal parasitic infections among expatriate workers in various occupations in Sharjah, United Arab Emirates. *Rev Inst Med Trop Sao Paulo* 2017;59(e82)
31. Al-Maniri A, Fochsen G, Al-Rawas O, et al. Immigrants and health system challenges to TB control in Oman. *BMC Health Services Research* 2010;10:210.
32. Pattisson P. Majority of Nepal migrant deaths "should be treated as murder", Global development. The Guardian [Internet]. The Guardian. 2014 . https://www.theguardian.com/global-development/2014/may/20/nepal-migrant-deaths-treated-murder (21 October, 2019, date last accessed). 2014
33. Pradhan B, Kjellstrom T, Atar D, et al. Heat stress impacts on cardiac mortality in Nepali migrant workers in Qatar. *Cardiology* 2019;143(1):37-48.

For peer review only - http://bmjopen.bmj.com/site/about/guidelines.xhtml

34. Yang H, Gao J, Wang T, et al. Association between adverse mental health and an unhealthy lifestyle in rural-to-urban migrant workers in Shanghai. *J Formosan Medl Assoc* 2017;116(2):90-98.
35. JPAN. Migrant worker and mental health in Nepal. *J Psych Assoc of Nepal* 2014;1(1)
36. Poudel A. Mental health of migrant workers is a pressing issue, but it has been ignored [Internet].https://kathmandupost.com/valley/2019/05/18/mental-health-of-migrant-workers-is-a-pressing-issue-but-it-has-been-ignored (21 Octomber, 2019, date last accessed).
37. Chapagai M, Pant S, Tulachan P, et al. Psychiatric morbidity among repatriated Nepalese foreign labor migrants-a hospital based study. *J Instit Med* 2017;41(1)
38. Maselko J. Social epidemiology and global mental health: expanding the evidence from high-income to low-and middle-income countries. *Current Epid Reports* 2017;4(2):166-73.
39. Weston G, Zilanawala A, Webb E, et al. Long work hours, weekend working and depressive symptoms in men and women: findings from a UK population-based study. *J Epid Com Health* 2019;73(5):465-74.
40. Donini A. Social suffering and structural violence: Nepali workers in Qatar. *Int Dev Policy* 2019:178-99. http://journals.openedition.org/poldev/3077 (24 October, 2019, date last accessed).
41. Murphy JM, Olivier DC, Monson RR, et al. Depression and anxiety in relation to social status: A prospective epidemiologic study. *Archives of General Psychiatry* 1991;48(3):223-29.
42. Lao CK, Chan YM, Tong HHY, et al. Underdiagnosis of depression in an economically deprived population in Macao, China. *Asia-Pacific Psychiatry* 2016;8(1):70-79.
43. Pulkki-Råback L, Ahola K, Elovainio M, et al. Socio-economic position and mental disorders in a working-age Finnish population: the health 2000 study. *The European J of Public Health* 2011;22(3):327-32.
44. Poudel KC, Jimba M, Okumura J, et al. Migrants' risky sexual behaviours in India and at home in far western Nepal. *J Trop Med Hyg* 2004;9(8):897-903.
45. Bam K, Thapa R, Newman MS, et al. Sexual behavior and condom use among seasonal Dalit migrant laborers to India from Far West, Nepal: a qualitative study. *PLoS One* 2013;8(9):e74903.
46. Simkhada PP, Regmi PR, Van Teijlingen E, et al. Identifying the gaps in Nepalese migrant workers' health and well-being: a review of the literature. *J of Travel Med* 2017;24(4)
47. Aryal N, Regmi PR, van Teijlingen E, et al. Adolescents left behind by migrant workers: a call for community-based mental health interventions in Nepal. *WHO South-East Asia J of Public Health* 2019;8(1):38-41.
48. Aryal N, Regmi P, Teijlingen E, et al. Knowing is not enough: migrant workers' spouses vulnerability to HIV. *SAARC J TB, Lung Diseases and HIV/AIDS* 2016;13(1):9-15.
49. Thapa S, Bista N, Hannes K, et al. Vulnerability of wives of Nepalese labor migrants to HIV infection: integrating quantitative and qualitative evidence. *Women and Health* 2016;56(7):745-66.
50. Urmi AZ, Leung DT, Wilkinson V, et al. Profile of an HIV testing and counseling unit in Bangladesh: majority of new diagnoses among returning migrant workers and spouses. *PloS One* 2015;10(10):e0141483.

For peer review only - http://bmjopen.bmj.com/site/about/guidelines.xhtml

51. Ning C, Jiang J, Ye L, et al. Comparison of three intervention models for promoting circumcision among migrant workers in western China to reduce local sexual transmission of HIV. *PloS one* 2013;8(9):e76107.
52. Seddon D, Adhikari J, Gurung G. Foreign labor migration and the remittance economy of Nepal. *Critical Asian Studies* 2002;34(1):19-40.
53. Bhandari P. Relative deprivation and migration in an agricultural setting of Nepal. *Popn and Envt* 2004;25(5):475-99.
54. Sadarangani SP, Lim PL, Vasoo S. Infectious diseases and migrant worker health in Singapore: a receiving country's perspective. *J of Travel Med* 2017;24(4)
55. Castelli F, Sulis G. Migration and infectious diseases. *Clinical Microb Infect* 2017;23(5):283-89.
56. Colombini M, Mayhew SH, Hawkins B, et al. Agenda setting and framing of gender-based violence in Nepal: how it became a health issue. *Health Policy and Planning* 2015;31(4):493-503.
57. Gioli G, Maharajan A, M G. Neither heroines nor victims: Women migrant workers and changing family and community relations in Nepal [Internet]. 2017. https://www.refworld.org/pdfid/5a1bf0374.pdf (21 October, 2019, date last accessed).
58. TheWorldBank. Migration and remittances factbook 2016 advanced edition [Internet]. 2016.https://siteresources.worldbank.org/INTPROSPECTS/Resources/334934-1199807908806/4549025-1450455807487/Factbookpart1.pdf (21 October, 2019, date last accessed)
59. WOREC. Women and migration. [Internet] 2012. https://issuu.com/worecnepal /docs/migration-and-women (24 Nov, 2019, date last accessed).
60. Pyakurel UP. Restrictive labour migration policy on Nepalese women and consequences. *Sociology and Anthropology* 2018;6(8):650-56.

For peer review only - http://bmjopen.bmj.com/site/about/guidelines.xhtml

Table 1: Characteristics of studies included

Author, Year, Country	Study Design and Setting	Participant Characteristics	Health Outcomes (Measurement Tools)	Key Findings
**Health risk and experiences related issues**				
**Adhikary et al; 2018, Nepal**	Cross-sectional questionnaire-based survey	Male Nepalese construction workers, worked in host countries (Malaysia, Qatar and Saudi Arabia) for >6 months.<br>Total participants – 403<br>Gender –NR<br>Age - NR	Self-reported health and wellbeing status	• 13.2% (n=53) reported poor/very poor health, relating to:<br>- Age older than 40 year reported as poor health (OR= 3.0, 95% 1.0-9.0)<br>- Poor work environment (OR= 6.8, 95% 3.2 – 14.6)<br>- Health risks at work (OR= 4.7, 95% 2.1-10.5)<br>- Prevalence of mental health issues was 23% overall - strong link between perceived health risks and mental health status.
**Adhikary et al; 2017, Nepal**	Cross-sectional questionnaire-based survey	Male Nepalese construction and factory workers, worked >6 months in Malaysia, Qatar or Saudi Arabia.<br>Gender –NR<br>Age - NR	Self-reported perceived health risks and accidents at work	**<u>Perceived health risks at work</u>**<br>• Poor or very poor work environment (rated by the workers) associated with greater perceived health risk at work OR 2.5, 95% CI=1.5-4.4<br>• Prevalence of accidents at work=17%<br>- Variables associated with accidents at work included: >Age 40 and above vs 20-29 (OR= 4.0, 95% CI=1.7-9.7)<br>- Not Satisfied accommodation vs satisfied with accommodation OR=1.9, 95% CI=1.1-3.4)<br>- Poor or very poor work environment vs good/good to fair environment (OR 3.5, 95% CI=1.8-6.7)<br>- Working in Middle-East vs Malaysia. (OR .3.6, 95% CI=1.5-8.5)<br>• Not registered with a doctor vs registered (OR=0.3, 95% CI=0.1-0.7)
**Simkhada et al; (2017), Nepal**<br><br>***[Data for GCCs and Malaysia provided by the authors)***	Retrospective analysis of NGO collected data (July 2009 to July 2014) via Paurakhi Nepal (NGO)	Returnee Nepalese female migrant workers from GCC [/Malaysia<br>(n= 942 = GCC = 933, Malaysia = 9)<br>Median age 31 (IQR 37)<br>Age range – 14-51 years	Various health issues while working in GCC, middle-east and Malaysia (prevalence calculated using information available from client Information Form/Sheet)	• Proportion female with f health problems - 24% (n=226)<br>- Abuse at workplace -41% (n=346)<br>- Accident at workplace - 1.1% (n=10)<br>- Mental health problem - 8.3% (n=78)<br>- Torture or maltreatment at the workplace 30.9% (n=291)<br>- Pregnancy at work place - 3.1% (n=29)<br>- Sexual abuse – 51.7% (n -15/29)<br>- Physical harm -10.9% (n=103)<br>Received health services – 10.8% (n=102)
**Min et al; 2016, Malaysia**	Retrospective analysis of routine healthcare data (January 2011 to December 2013)	Patients attending to the eye casualty with work-related ocular injuries, in Hospital Sultan Ismail in Johor Bahru, Malaysia from	Work related ocular traumas	• 33 cases of Nepalese work-related eye injuries. Causes range from open globe injuries due to being hit by a machine, nail, wood and metal whilst grinding.

For peer review only - http://bmjopen.bmj.com/site/about/guidelines.xhtml

Author, Year, Country	Study Design and Setting	Participant Characteristics	Health Outcomes (Measurement Tools)	Key Findings
		Total 440 work-related ocular traumas.<br>Nepalese – 21.71% (n=33)<br>Gender –NR<br>Age - NR		
**Al-Thani et al; 2015, Qatar**	Retrospective analysis of hospital trauma registry records 2010-2013 Hamad Trauma Centre	Total migrant workers - 2015<br>Nepalese –28% (n=563)<br>Male – 98% (n=1972)<br>Female –2% (n=43)<br>Age - NR	Proportion of occupational injuries and mortality cases	• Overall proportion of occupational injury cases – 27.9% (n= 563), of which<br>- Falls from height - 52% (n=295)<br>- Fall of a heavy object - 21% (n= 115)<br>- Motor vehicle crashes injuries - 17% (n=97)<br>- Machinery injuries - 5% (n= 27)<br>• Others – 5% (n=29)
**Latifi et al; 2015, Qatar**	Retrospective analysis of routine healthcare data	Total traffic related pedestrian injuries (TRPI) patients – 601<br>Total Nepalese expat TRPI patients – 24% (147)<br>Gender –NR<br>Age - NR	Pedestrian morbidity and mortality	• 25.4% of TRPI were of Nepalese migrant workers (vs 16.0% of the general population of Qatar being Nepalese).<br>• 51.4% of TRPI with positive blood alcohol were Nepalese migrant workers.
**Joshi et al, 2014; Nepal**	Cross-sectional study	Nepalese migrants with experience of >6 months in Qatar, Saudi Arabia or United Arab Emirates.<br>Total study size – 408<br>Males – 92.4% (n=377)<br>Aged between 26-35 – 53.4% (n=218)	Knowledge of HIV/AIDS and risk perceptions	• Risk perceptions of HIV/AIDS:<br>- Concerned about HIV/AIDS – 90.8% (n=366)<br>- Perceived themselves at high risk of being infected due to their sexual activities - 59.2% (n=397)<br>• Sexual behaviour:<br>• 17.2% (n=70) had sexual intercourse with a partner other than their spouse during the last 12 months of their stay abroad.
**Alswaidi et al; 2013, Saudi Arabia**	Review of Ministry and Health data from Saudi expat worker fitness screening programme (1997–2010)	Total number of registered expatriate workers - 4 272 480<br>Nepalese – 0.9% (n=38 908).<br>Females – 14% (n=5 367)<br>Males – 86% (n=33 541)<br>Age - NR	Proportion of 'unfit' to workers.	• Cases of unfitness among Nepalese workers by gender:<br>- Unfit males – 1.99% (n=669)<br>- Unfit females – 1.19% (n=64)<br>- Overall unfit – 1.88% (n=733)<br>• Nepalese migrants were the third most unfit population.<br>• Nepalese migrants as proportion of all those with:<br>- Infectious causes of unfitness (incl. hepatitis, HIV, TB) – 1.6% (n=379)<br>- Non-communicable causes of unfitness – 5.3% (n= 354)
**Joshi et al, 2011; Nepal**	Cross-sectional questionnaire survey, Kathmandu (International	Returnee Nepalese male and female migrant workers from Qatar, Saudi Arabia and UAE (n=408)	Prevalence of health problems using self-reported/ questionnaire survey	• Prevalence of health problem(s) – 56.6% (n=231)<br>• Most common problems:<br>- Headache or fever - 30.7% (n=71)<br>- Respiratory symptoms - 21.2% (n=49)

For peer review only - http://bmjopen.bmj.com/site/about/guidelines.xhtml

Author, Year, Country	Study Design and Setting	Participant Characteristics	Health Outcomes (Measurement Tools)	Key Findings
	Airport and nearby hotels/lodges).	Male = 377 (92.4%)<br>Female = 31 (7.6%)<br>Mean Age (SD) – 32 (6.5) years<br>Age ranges – 18-53 years		- Musculoskeletal problems – 19.9% (n=46)<br>- Gastrointestinal illness – 19.5% (n=15)<br>- Injuries/poisoning – 13.9% (n=32)<br>• Prevalence of some type of injury or accident at their workplace - 25% (n=102)<br>• Health insurance in host countries- 36.5% (n=149)<br>• Sought health services or treatment in the working countries- 83.1% (n=192)<br>• Lack of provision of leave during health problem(s)- 48.7% (n=19)
**Infectious diseases related issues**				
**Sahimin et al; 2018, Malaysia**	Cross-sectional study	Migrant workers in Malaysia.<br>Total stool samples examined – 388<br>Nepalese –20.9% (81)<br>Gender –NR<br>Age - NR Gender –NR<br>Age - NR	Prevalence and species identity of hookworms causing infection	• 21.4% (n=~11) of Nepalese migrant worker samples positive for hookworms.
**Dafalla et al; 2017, UAE**	Cross-sectional survey conducted at public health clinic	Immigrant workers – food handlers, babysitters, housemaids, drivers working in Sarjaha, UAE<br>Total sample – 21,347 (number of Nepalese workers not reported)<br>Total infected population –3.3% (n=708)<br>Gender –NR<br>Age - NR	Prevalence of parasitic infections<br>(Examined microscopically and screened for intestinal parasites)	• Proportion of infected migrant workers that are Nepalese – 6.2% (n=44)<br>- All protozoal infections: 7% (n=33)<br>• All helminth infections: 4.2% (n=9)
**Noordin et al; 2017, Malaysia**	Cross-sectional survey (September 2014 to August 2015)	484 migrant workers from manufacturing, services, agriculture and plantation, construction and domestic work sectors.<br>Nepalese – 21.3% (n=103)<br>Gender –NR<br>Age - NR	Prevalence of parasitic infections	• Sero-prevalence of brugian Lymphatic Filariasis [BmR1] – 2.9% (n=3)<br>• Prevalence of parasitic infections (BmSXP) –12.6% (n=13)
**Sahimin et al; 2017, Malaysia**	Correctional survey (Sept. 2014- Aug. 2015)	484 migrant workers<br>Nepalese respondents- 20.5% (n = 99)	Sero-prevalence<br>T. gondii through Questionnaire survey	• Sero-prevalence:<br>- IgG – 74.7% (n = 99)

For peer review only - http://bmjopen.bmj.com/site/about/guidelines.xhtml

Author, Year, Country	Study Design and Setting	Participant Characteristics	Health Outcomes (Measurement Tools)	Key Findings
		Conducted at five working sectors (manufacturing, construction, plantation, domestic and food services)	and laboratory blood tests	• lgM – 6.1% (n=99)
**Woh et al; 2017, Malaysia**	Cross-sectional study	Healthy, asymptomatic migrant food handlers.<br>Total study size – 317<br>Nepalese – 25.2% (n=80)<br>Gender –NR<br>Age - NR	Prevalence of Salmonella carriers, using stool samples.	• Prevalence of salmonella amongst Nepalese migrant food handlers – 3.7% (n=3)
**Abu-Madi et al; 2016a, Qatar**	Retrospective analysis of routine healthcare data (2005 to 2014)	Records held at Hamad Medical Corporation data-base for subjects referred for stool examination - 29,286<br>Nepalese – 4.8% (n=1429)<br>Gender –NR<br>Age - NR	Proportion of helminth infections positive cases.	• Highest proportion of helminth infections among Nepalese workers – 15.26 % 95% CI (13.39–17.12)
**Abu-Madi et al; 2016b, Qatar**	Retrospective analysis of routine healthcare data	Recently arrived migrant workers in Qatar – 2,486<br>Nepalese – 15% (n=373)<br>Gender –NR<br>Age - NR	Presence of intestinal parasites (helminths and protozoa)	• Proportion of positive cases in Nepalese migrant workers:<br>- Helminths combined - 6.2% 95% CI 3.86–9.65<br>- Hookworms - 4.3% 95% CI 2.40–7.35<br>Protozoa combined - 13.7% 95% CI 10.05–18.23
**Humphery et al; 2016, Qatar**	Prospective community-based survey, Doha	Total sample – 126<br>Nepalese – 29.3% (n=37)<br>All male population<br>Median age (IQR) in years = 33 (27–39)	Prevalence of gastrointestinal pathogens (detected using polymerase chain reaction)	• Total prevalence of gastrointestinal pathogens = 62.7% (n=79)<br>• Gastrointestinal pathogens amongst Nepalese migrant workers – 26.6% (n=21)
**Imam et al; 2015, Qatar**	Retrospective analysis of routine healthcare data (January 2006 and December 2012)	Patients with suspected or confirmed tuberculous meningitis. Total study population – 80<br>Nepalese – 37% (n=30)<br>Gender –NR<br>Age - NR	Clinical presentation, diagnosis, treatment, outcome, and the incidence of adult tuberculous meningitis	• 30/80 patients with tuberculous meningitis were Nepalese (37.5%). No further data.
**Kavarodi et al; 2014, Qatar**	Population-based cross-sectional study	Low income expatriate workers from Indian sub-continent (living in Qatar for >6 months)<br>Total participants – 3,946<br>Nepalese – 5.4% (n=213)	Clinical prevalence of suspected oral lesions.	• Oral Lesions in of Nepalese workers 4.69% (n=10).

For peer review only - http://bmjopen.bmj.com/site/about/guidelines.xhtml

Author, Year, Country	Study Design and Setting	Participant Characteristics	Health Outcomes (Measurement Tools)	Key Findings
		Gender –NR<br>Age - NR		
**Chattu and Mohammad, 2013, Saudi Arabia**	Retrospective analysis of routine healthcare data from Qassim region (January 2005 to December 2009)	Migrant workers (n= 165)<br>Male – 42% (n=70)<br>Female –58% (n=95)<br>Age - NR	Proportion of reported TB cases, using laboratory test	• Proportion of migrant workers with tuberculosis from Nepal: 7% (n=12).
**Ibrahim et al; 2009, Qatar**	Prospective community based survey, Alkhor hospital.	anti-HEV IgG Nepalese migrants nationally – 86<br>58 of these seen at Alkhor Hospital.<br>Gender –NR<br>Aged 26.7 (SD-5.6, range 19–41 years)	Prevalence of Hepatitis E (using ELISA test) and other clinical symptoms	• Prevalence of acute HEV amongst those seen at Alkhor Hospital – 74% (n=43)<br>Admitted to hospital – 95.3% (n=41)
**Chan et al; 2008, Malaysia**	Cross-sectional survey conducted in a plantation and detention camp of Malaysia	Total foreign migrant workers - 501<br>Nepalese – 5% (n=26)<br>Gender –NR<br>Age - NR	*Toxoplasma gondii* IgG and IgM sero-prevalence	• Prevalence of Toxoplasma gondii IgG – 46.2% (n=12)<br>• Prevalence of Toxoplasma gondii IgM – 11.5% (n=3)<br>•
**Al-Marri, 2001, Qatar**	Population-based retrospective analysis (January 1996 to December 1998)	Total cases of positive *M. tuberculosis* culture and sensitivity – 406<br>Nepalese migrant cases – 11% (n=44)<br>Gender –NR<br>Age - NR	Drug resistant cases of TB (where positive isolates identified)	- Of total 386 cases of pulmonary TB (321 expats) identified, 44 Nepalese cases of TB, of which 9 cases were drug resistant.

For peer review only - http://bmjopen.bmj.com/site/about/guidelines.xhtml

For peer review only

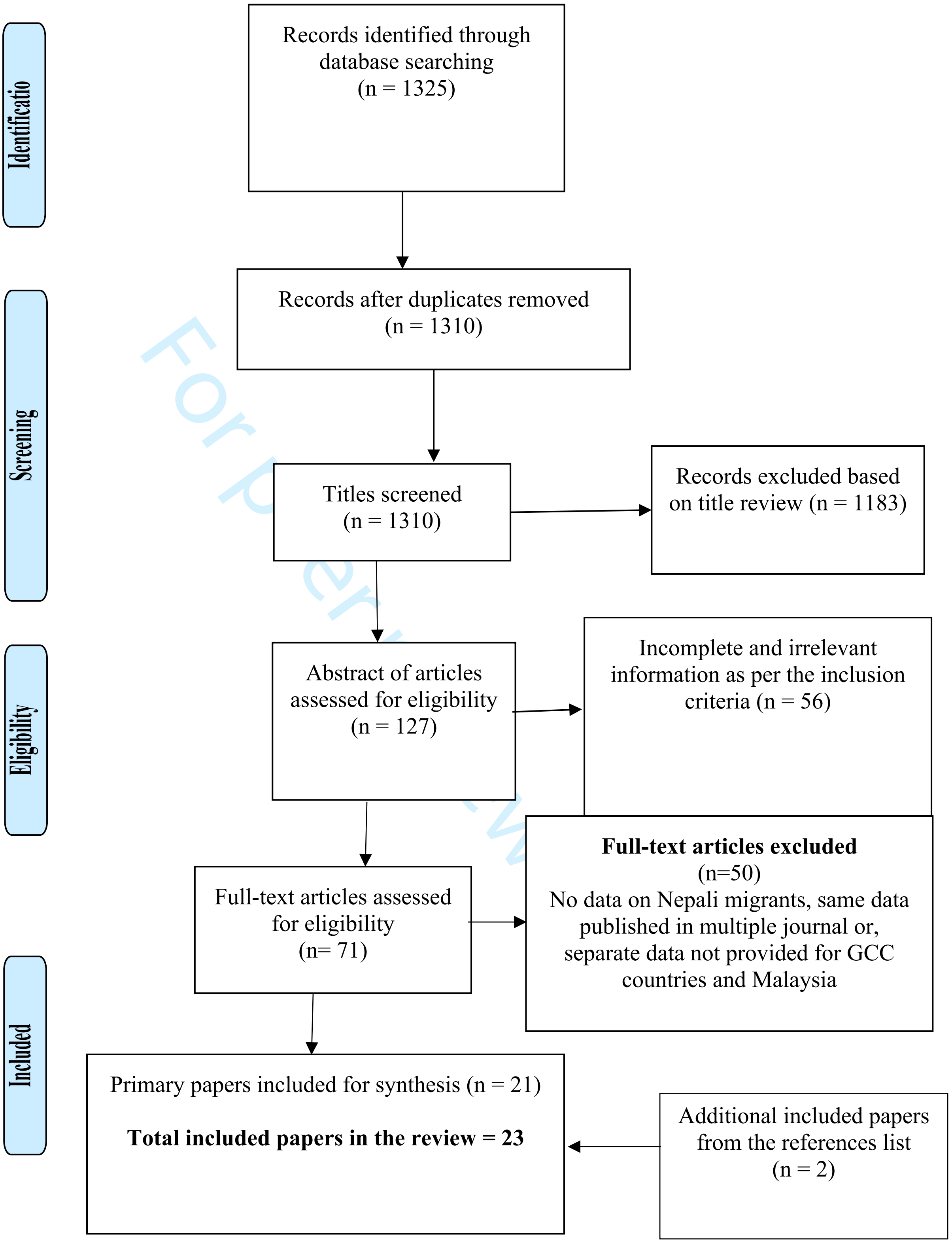

Figure 1: PRISMA Flow Diagram of Study Selection

Appendix 1 Search in MEDLINE

1. Migration {Including Related Terms}
2. Migrant {Including Related Terms}
3. Emigrant {Including Related Terms}
4. Immigrant{Including Related Terms}
5. Expatriate {Including Related Terms}
6. Foreign worker {Including Related Terms}
7. Labor migration {Including Related Terms}
8. Left-behind {Including Related Terms}
9. Migrant families {Including Related Terms}
10. Or/1-9
11. Nepal {Including Related Terms}
12. Nepalese {Including Related Terms}
13. Nepali {Including Related Terms}
14. UAE or United Araba Emirates {Including Related Terms}
15. GCC or Gulf Cooperating council {Including Related Terms}
16. Middle East {Including Related Terms}
17. Bahrain {Including Related Terms}
18. Saudi Arabia {Including Related Terms}
19. Oman {Including Related Terms}
20. Qatar {Including Related Terms}
21. Kuwait {Including Related Terms}
22. Malaysia {Including Related Terms}
23. Or/11-22

For peer review only

24. 10 AND 23

For peer review only

Appendix 2: Quality Assessment

Reference	Was the sample frame appropriate to address the target population?	Were study participants sampled in an appropriate way?	Was the sample size adequate?	Were the study subjects and the setting described in detail?	Was the data analysis conducted with sufficient cov erage of the identified sample?	Were valid methods used for the identification of the condition?	Was the condition measured in a standard, reliable way for all participants?	Was there appropriate statistical analysis?	Was the response rate adequate, and if not, was the low response rate managed appropriately?
**Overview N-23 studies**	**Yes - 22 (96%)**	**Yes - 17 (74%)**	**Yes - 15 (65%)**	**Yes – 19 (83%)**	**Yes – 21 (91%)**	**Yes – 22 (96%)**	**Yes – 21 (91%)**	**Yes – 20 (87%)**	**Yes – 17 (74%)**
	**Unclear - 1 (4%)**	**Unclear - 5 (22%)**	**Unclear – 8 (35%)**	**Unclear – 3 (13%)**	**Unclear – 2 (9%)**	**Unclear – 1 (4%)**	**Unclear – 2 (9%)**	**Unclear – 3 (13%)**	**Unclear – 6 (26%)**
	**No - 0**	**No - 1 (4%)**	**No - 0**	**No – 1 (4%)**	**No - 0**	**No - 0**	**No - 0**	**No - 0**	**No - 0**
Abu-Madi et al; 2016	Yes	Yes	Yes	Yes	Yes	Yes	Yes	Yes	Yes
Abu-Madi et al; 2016	Yes	Yes	Yes	Unclear	Yes	Yes	Yes	Unclear	Yes
Adhikari et at; 2017	Yes	Yes	Yes	Yes	Yes	Yes	Yes	Yes	Yes
Adhikari et at; 2018	Yes	Yes	Yes	Yes	Yes	Yes	Yes	Yes	Yes
Al-Marri et al ; 2001	Yes	Yes	Yes	Yes	Yes	Yes	Yes	Unclear	Yes
Alswaidi et al 2013	Yes	Yes	Yes	Yes	Yes	Yes	Yes	Yes	Yes
Al-Thani et al; 2015	Yes	Unclear	Yes	Yes	Yes	Yes	Unclear	Yes	Yes
Chan et al; 2008	Unclear	Yes	Yes	Yes	Yes	Yes	Yes	Yes	Yes
Chattu et al; 2013	Yes	Yes	Unclear	Yes	Unclear	Yes	Unclear	Yes	Unclear
Dafalla et al; 2017	Yes	Unclear	Yes	Yes	Yes	Yes	Yes	Yes	Unclear
Humphery et al; 2016	Yes	Unclear	Unclear	Yes	Yes	Yes	Yes	Yes	Unclear
Ibrahim et al; 2009	Yes	Yes	Unclear	Yes	Unclear	Yes	Yes	Yes	Unclear
Imam et al; 2015	Yes	Yes	Unclear	Yes	Yes	Yes	Yes	Yes	Yes
Joshi et al; 2011	Yes	Yes	Yes	Yes	Yes	Yes	Yes	Yes	Yes
Joshi et al; 2014	Yes	No	Yes	Yes	Yes	Unclear	Yes	Unclear	Yes
Kavarodi et al; 2014	Yes	Yes	Yes	Yes	Yes	Yes	Yes	Yes	Yes
Latifi et al; 2015	Yes	Yes	Yes	Unclear	Yes	Yes	Yes	Yes	Yes
Min, 2016	Yes	Yes	Unclear	Yes	Yes	Yes	Yes	Yes	Yes
Noordin et al; 2017	Yes	Yes	Unclear	Unclear	Yes	Yes	Yes	No	Unclear
Sahimin et al; 2017	Yes	Yes	Yes	Yes	Yes	Yes	Yes	Yes	Yes
Sahimin, 2018	Yes	Yes	Yes	Yes	Yes	Yes	Yes	Yes	Yes
Simkhada et al; 2017	Yes	Unclear	Unclear	Yes	Yes	Yes	Yes	Yes	Unclear
Who et al; 2017	Yes	Unclear	Unclear	Yes	Yes	Yes	Yes	Yes	Yes

For peer review only

For peer review only

PRISMA 2009 Checklist

Section/topic	#	Checklist item	Reported on page #
**TITLE**			
Title	1	Identify the report as a systematic review, meta-analysis, or both.	1
**ABSTRACT**			
Structured summary	2	Provide a structured summary including, as applicable: background; objectives; data sources; study eligibility criteria, participants, and interventions; study appraisal and synthesis methods; results; limitations; conclusions and implications of key findings; systematic review registration number.	2
**INTRODUCTION**			
Rationale	3	Describe the rationale for the review in the context of what is already known.	4
Objectives	4	Provide an explicit statement of questions being addressed with reference to participants, interventions, comparisons, outcomes, and study design (PICOS).	4
**METHODS**			
Protocol and registration	5	Indicate if a review protocol exists, if and where it can be accessed (e.g., Web address), and, if available, provide registration information including registration number.	5
Eligibility criteria	6	Specify study characteristics (e.g., PICOS, length of follow-up) and report characteristics (e.g., years considered, language, publication status) used as criteria for eligibility, giving rationale.	5
Information sources	7	Describe all information sources (e.g., databases with dates of coverage, contact with study authors to identify additional studies) in the search and date last searched.	5
Search	8	Present full electronic search strategy for at least one database, including any limits used, such that it could be repeated.	Appendix 1
Study selection	9	State the process for selecting studies (i.e., screening, eligibility, included in systematic review, and, if applicable, included in the meta-analysis).	5
Data collection process	10	Describe method of data extraction from reports (e.g., piloted forms, independently, in duplicate) and any processes for obtaining and confirming data from investigators.	6
Data items	11	List and define all variables for which data were sought (e.g., PICOS, funding sources) and any assumptions and simplifications made.	5
Risk of bias in individual studies	12	Describe methods used for assessing risk of bias of individual studies (including specification of whether this was done at the study or outcome level), and how this information is to be used in any data synthesis.	6
Summary measures	13	State the principal summary measures (e.g., risk ratio, difference in means).	6
Synthesis of results	14	Describe the methods of handling data and combining results of studies, if done, including measures of consistency (e.g., I^2) for each meta-analysis.	6

For peer review only - http://bmjopen.bmj.com/site/about/guidelines.xhtml

PRISMA 2009 Checklist

Section/topic	#	Checklist item	Reported on page #
Risk of bias across studies	15	Specify any assessment of risk of bias that may affect the cumulative evidence (e.g., publication bias, selective reporting within studies).	6
Additional analyses	16	Describe methods of additional analyses (e.g., sensitivity or subgroup analyses, meta-regression), if done, indicating which were pre-specified.	NA
**RESULTS**			
Study selection	17	Give numbers of studies screened, assessed for eligibility, and included in the review, with reasons for exclusions at each stage, ideally with a flow diagram.	6,7
Study characteristics	18	For each study, present characteristics for which data were extracted (e.g., study size, PICOS, follow-up period) and provide the citations.	8-10
Risk of bias within studies	19	Present data on risk of bias of each study and, if available, any outcome level assessment (see item 12).	Appendix 2
Results of individual studies	20	For all outcomes considered (benefits or harms), present, for each study: (a) simple summary data for each intervention group (b) effect estimates and confidence intervals, ideally with a forest plot.	8-10
Synthesis of results	21	Present results of each meta-analysis done, including confidence intervals and measures of consistency.	NA
Risk of bias across studies	22	Present results of any assessment of risk of bias across studies (see Item 15).	Appendix 2
Additional analysis	23	Give results of additional analyses, if done (e.g., sensitivity or subgroup analyses, meta-regression [see Item 16]).	NA
**DISCUSSION**			
Summary of evidence	24	Summarize the main findings including the strength of evidence for each main outcome; consider their relevance to key groups (e.g., healthcare providers, users, and policy makers).	10-14
Limitations	25	Discuss limitations at study and outcome level (e.g., risk of bias), and at review-level (e.g., incomplete retrieval of identified research, reporting bias).	13
Conclusions	26	Provide a general interpretation of the results in the context of other evidence, and implications for future research.	10-14
**FUNDING**			
Funding	27	Describe sources of funding for the systematic review and other support (e.g., supply of data); role of funders for the systematic review.	14

From: Moher D, Liberati A, Tetzlaff J, Altman DG, The PRISMA Group (2009). Preferred Reporting Items for Systematic Reviews and Meta-Analyses: The PRISMA Statement. PLoS Med 6(7): e1000097. doi:10.1371/journal.pmed1000097

For peer review only - http://bmjopen.bmj.com/site/about/guidelines.xhtml

PRISMA 2009 Checklist

For peer review only

For peer review only - http://bmjopen.bmj.com/site/about/guidelines.xhtml

BMJ Open

Health and Wellbeing Issues of Nepalese Migrant Workers in the Gulf Cooperation Council Countries and Malaysia: A Systematic Review

Journal:	*BMJ Open*
Manuscript ID	bmjopen-2020-038439.R1
Article Type:	Original research
Date Submitted by the Author:	29-Jun-2020
Complete List of Authors:	Paudyal, Priyamvada; Brighton and Sussex Medical School Division of Primary Care and Public Health Medicine<br>Kulasabanathan, Kavian ; Brighton and Sussex Medical School Division of Primary Care and Public Health Medicine<br>Cassell, Jackie; Brighton and Sussex Medical School Division of Primary Care and Public Health Medicine<br>Memon, Anjum; Brighton and Sussex Medical School Division of Primary Care and Public Health Medicine<br>Simkhada, Padam; University of Huddersfield, School of Human and Health Sciences<br>Wasti, Sharada; Green Tara Nepal
**Primary Subject Heading**:	Global health
Secondary Subject Heading:	Public health
Keywords:	PUBLIC HEALTH, EPIDEMIOLOGY, Health policy < HEALTH SERVICES ADMINISTRATION & MANAGEMENT

SCHOLARONE™
Manuscripts

I, the Submitting Author has the right to grant and does grant on behalf of all authors of the Work (as defined in the below author licence), an exclusive licence and/or a non-exclusive licence for contributions from authors who are: i) UK Crown employees; ii) where BMJ has agreed a CC-BY licence shall apply, and/or iii) in accordance with the terms applicable for US Federal Government officers or employees acting as part of their official duties; on a worldwide, perpetual, irrevocable, royalty-free basis to BMJ Publishing Group Ltd ("BMJ") its licensees and where the relevant Journal is co-owned by BMJ to the co-owners of the Journal, to publish the Work in this journal and any other BMJ products and to exploit all rights, as set out in our licence.

The Submitting Author accepts and understands that any supply made under these terms is made by BMJ to the Submitting Author unless you are acting as an employee on behalf of your employer or a postgraduate student of an affiliated institution which is paying any applicable article publishing charge ("APC") for Open Access articles. Where the Submitting Author wishes to make the Work available on an Open Access basis (and intends to pay the relevant APC), the terms of reuse of such Open Access shall be governed by a Creative Commons licence – details of these licences and which Creative Commons licence will apply to this Work are set out in our licence referred to above.

Other than as permitted in any relevant BMJ Author's Self Archiving Policies, I confirm this Work has not been accepted for publication elsewhere, is not being considered for publication elsewhere and does not duplicate material already published. I confirm all authors consent to publication of this Work and authorise the granting of this licence.

For peer review only

Health and Wellbeing Issues of Nepalese Migrant Workers in the Gulf Cooperation Council Countries and Malaysia: A Systematic Review

Priyamvada Paudyal[1], Kavian Kulasabanathan[1], Jackie Cassell[1], Anjum Memon[1], Padam Simkhada[2], Sharada Pd. Wasti[3]

[1]Department of Primary Care and Public Health, Brighton and Sussex Medical School, Brighton UK; [2]Faculty of Health, University of Huddersfield, UK; [3]Green Tara Nepal, Kathmandu, Nepal

Corresponding Author: Dr Priyamvada Paudyal
Department of Primary Care and Public Health, Brighton and Sussex Medical School, Room 322, Watson Building, Village Way, Falmer, BRIGHTON, BN1 9PH, UK
+44 (0) 1273 644548; p.paudyal@bsms.ac.uk

Running Title: Health issues of Nepalese migrant workers in Gulf Countries and Malaysia

For peer review only

For peer review only - http://bmjopen.bmj.com/site/about/guidelines.xhtml

Abstract: Approximately 3.8 million Nepalese nationals (14% of the total population) work abroad. This systematic review summarises the evidence on health and wellbeing of Nepalese migrant workers in the Gulf Cooperation Council (GCC) countries and Malaysia, the destination countries for 85% of Nepalese migrant workers. A combination of migration and country specific search terms were used to identify relevant studies using EMBASE, MEDLINE, Scopus and Global Health databases. Studies were eligible if they: 1) included Nepalese migrant workers aged ⩾18 years working currently in the GCC countries or Malaysia or returnee migrant workers from these countries; 2) were primary studies that investigated health and wellbeing status/issues; and 3) were published in English language before 8 May 2020. All the included studies were assessed using the Joanna Briggs Institute Checklist for Prevalence Studies. The review protocol can be found at http://sro.sussex.ac.uk/id/eprint/86400/.

A total of 33 studies were eligible for inclusion; 12 studies were conducted in Qatar, eight in Malaysia, nine in Nepal, two in Saudi Arabia and one each in UAE and Kuwait. Nearly half of the studies (n=16) scored as 'high' quality and the rest (n=17) as 'moderate' quality. Five key health and wellbeing related issues were identified in this population: a) occupational hazards; b) sexual health; c) mental health; d) healthcare access and e) infectious diseases. To our knowledge, this is the most comprehensive review of the health and well-being of Nepalese migrant workers in the GCC countries and Malaysia. This review highlights an urgent need to identify and implement policies and practices across Nepal and destination countries to protect the health and wellbeing of migrant workers.

Key Terms: Migration, GCC countries, Health, Wellness, Nepalese

Word Limit 300

Word Count: 268

Strengths and Limitations

- This review is the most comprehensive review to date on this population.
- The review did not restrict studies based on particular health outcomes, peer reviewed studies looking at a range of health issues in this population were included.
- Meta-analysis was not conducted as there was heterogeneity in the outcome measured and the measurement tools used in the studies.

For peer review only - http://bmjopen.bmj.com/site/about/guidelines.xhtml

For peer review only

Introduction

Migration is the overarching narrative of our time, and its impact is increasingly being recognised in global public health agendas. The United Nations (UN) Sustainable Development Goals (SDGs) identify migration as a catalyst for development and recommend that 'no-one should be left behind' to achieve Universal Health Coverage for all[1]. According to the World Migration Report 2020, the number of international migrants has reached approximately 272 million, and two third of these are estimated to be labour migrants[2]. Labour migration has been a key determinant of population changes in Asia, especially in Gulf Cooperation Council (GCC) countries, a major destination for workers within Asia[2].

Nepal is a low-income country going through a demographic transition, with an ageing population and attendant chronic diseases. Approximately 3.8 million Nepalese (14% of the total population) work abroad (not counting those working in India)[3]. The Nepal Demographic and Health Survey (2016) reported that nearly half (47%) the households have at least one family member who migrated in the last 10 years either in internal or international destinations[4]. These migrant workers contribute over a quarter (26.9%) of the country's gross domestic product (GDP) through remittance from abroad[5]. The most recent Nepalese Government report shows that the migration outflow consists predominantly of low-skilled male workers, primarily to Malaysia and the GCC countries[5].

Labour migration contributes significantly to the sociocultural and economic development of both origin and destination countries. However, migrant workers experience specific vulnerabilities, and face a range of health risks while working abroad. These risks are particularly significant for Nepalese workers in the GCC countries, as they are often employed in occupations considered 'difficult, dirty, and dangerous (3Ds)'. These are sectors with higher occupational risks such as agriculture, construction, transport and heavy industry. Furthermore, Nepalese migrant workers consistently work for longer hours as compared to native workers[6 7] and are often exposed to factors which promote poor health and wellbeing, including low wages, poor housing, an unhealthy diet, and difficulty in accessing health services[6, 8]. Many Nepalese migrant workers die abroad every year including a significant number that are unexplained, while a large number return home with debilitating injuries, and both mental and physical illness[6]. This systematic review identified and summarised the evidence from primary studies on the health and wellbeing of Nepalese migrant workers in the GCC countries and

For peer review only - http://bmjopen.bmj.com/site/about/guidelines.xhtml

Malaysia, the destination countries for 85% of labour migration. This review was conducted as a part of University of Sussex internally funded Global Challenges Research Fund (GCRF) project to develop a culturally relevant intervention to support the health and wellbeing of Nepalese migrant workers in GCC countries.

Methods

Protocol Registration

This study protocol was registered at the University of Sussex (http://sro.sussex.ac.uk/id/eprint/86400/). The study followed the PRISMA (Preferred Reporting Items for Systematic Reviews and Meta-Analyses) guidelines and recommendations of the Cochrane Collaboration (www.prisma-statement.org).

Electronic Search

A combination of migration specific search terms (migration, migrant, emigrant, immigrant, expatriate, foreign worker, labor migration, left-behind, migrant families) and country specific search terms (Nepal, Nepalese, Nepali, UAE, United Arab Emirates, GCC, Gulf Cooperation Council, Middle East, Bahrain, Saudi Arabia, Oman, Qatar, Kuwait, Malaysia) were used to identify relevant studies using EMBASE, MEDLINE, Scopus and Global Health databases (Appendix 1). The search aimed to identify all relevant studies regardless of any health outcomes used. As such, no health outcome specific terms were used to limit the electronic search. Reference lists of the relevant studies including those of related systematic reviews and reference lists of the selected studies were further screened to identify potentially eligible studies.

Inclusion and Exclusion Criteria

Studies were eligible if they: 1) included Nepalese migrant workers aged ⩾18 years working currently in the GCC countries or Malaysia or returnee migrant workers from these countries; 2) provided primary data on health and wellbeing status/issues (physical health, mental health, accidents and injuries); and 3) were published in English language before 8 May 2020.

Article Screening and Selection

Once the electronic search was completed, the identified articles were exported to Rayyan (https://rayyan.qcri.org/welcome) and screening was carried out by two reviewers (SW and KK) independently to identify eligible articles. The titles of the identified studies were screened

For peer review only - http://bmjopen.bmj.com/site/about/guidelines.xhtml

to remove any duplicates and irrelevant articles. The abstract of all remaining articles was screened to identify eligible full text articles. Full text articles were reviewed and a consensus was reached to finalise the articles for inclusion. If more than one study were published using the same data source (e.g. routine healthcare date), we used the study with the largest sample size. Any disagreement over eligibility of studies was resolved through discussion with the third reviewer (PP).

Data Extraction and Synthesis

The information extracted from each article included: study reference (authors, publication year and country), study design and settings, participants' characteristics (sample size, age, and gender), health outcomes and key findings (Table 1). Extracted data were analysed and a summary of the narrative synthesis is reported in the results section.

Quality Assessment

The PRISMA guideline suggests that systematic review should assess the risk of bias (based on theoretical grounds) rather than study quality (the best authors could do in the setting). However, we assessed the latter as the studies included in this review were predominately cross-sectional in nature with methodological limitations[9]. Quality assessment for this review was done using the Joanna Briggs Institute (JBI) Critical Appraisal Tools[10]. 'Checklist for Prevalence Studies' was used for cross-sectional studies estimating the prevalence of the condition. The tool contains nine items covering domains related to sampling, outcome assessment, statistical analysis and response rate. Each item was scored one if the response was 'Yes' and scored zero if the response was 'NO' or 'Unclear'. As in the previous review[11], studies with eight or more 'Yes' response were rated as 'high' quality, four to seven as 'moderate' and three or below as 'low' quality. Similarly, 'Checklist for Analytical Cross-sectional Studies' was used for cross-sectional studies reporting effect sizes. The checklist contains eight items covering domains related to sampling, exposure, outcome, confounding factors, and statistical analysis (maximum possible score eight). Studies were categorised as high quality (seven or above), moderate quality (between five and six) or low quality (four and below). Qualitative studies were assessed by using 'Checklist for Qualitative Studies'. The checklist contains ten items with domains covering methodological approach, data collection, analysis and interpretation, researcher's role, participants' voice and ethics. The studies were rated high quality (eight and above), moderate quality (between five-seven) or low quality (four

For peer review only - http://bmjopen.bmj.com/site/about/guidelines.xhtml

and below) as on the previous publication[12]. The assessment was undertaken independently by two reviewers (SW and KK) with any discrepancies resolved by a third reviewer (PP). As the number of studies in this population is limited, we did not exclude studies based on quality assessment. The results of the quality assessment are presented in Appendix 2.

Results

Screening Results

Database searches yielded 2770 articles. After duplicate removal, titles of the 2562 articles were screened and 2253 were excluded. Abstracts of the remaining 309 publications were further screened and 215 of these were excluded. Full text screening of the remaining 94 papers were carried out and a further 61 papers were excluded for various reasons (Figure 1). Altogether, 33 papers were included in this review; 31 were quantitative and two were qualitative studies.

For peer review only

For peer review only - http://bmjopen.bmj.com/site/about/guidelines.xhtml

167 **Figure 1: PRISMA Flow Diagram of Study Selection**

For peer review only

For peer review only - http://bmjopen.bmj.com/site/about/guidelines.xhtml

Study Characteristics

A total of 33 papers were included in the review among them 12 studies were conducted in Qatar [13-24], eight in Malaysia[25-32], nine in Nepal[6 8 33-39], two in Saudi Arabia[40 41,] one each in UAE[42] and Kuwait [43]respectively. Two study included all GCC countries and Malaysia[8 37], whilst another included disaggregated data for the 'Gulf states' but did not specify particular name of the country[17]. Similarly, two study included Malaysia, Qatar and Saudi Arabia [33 34]and another two included in Qatar, Saudi Arabia and UAE[6 35] (Table 1). The study design varied across the studies; the review included 13 retrospective analysis of routine healthcare data [8 13-16 19 21 26 36 39-41 43], 18 cross-sectional studies[6 17 18 20 22-25 27-35 42]. Only two studies were qualitative in nature[37 38]. Nine studies focused specifically on Nepalese migrants as their primary study population [6 8 33-39] whilst the remaining 24 studies mentioned Nepalese migrant workers as part of a sub-analysis (Table 1). In majority of the studies, there was a lack of disaggregated data on demographic characteristics of Nepalese migrant workers. Studies were predominantly conducted in male migrant workers and there was a paucity of research with only one study focusing on female migrant workers[8]. The study mainly fell into two categories: those exploring the health risks and experiences of migrants while abroad and those focusing on infectious diseases (mostly done as a part of arrival screening).

Studies Exploring Health Risk and Experiences

Occupational Health and Hazards

Seven studies (four high quality and three moderate quality) specifically assessed occupational morbidity, mortality, and fitness to work in the destination countries [16 21 26 33 36 38 40]. Majority of these studies were conducted in male migrant workers and the sample of Nepalese migrants varied from 20 to 38,908. Adhikari et al (2017) reported that around one-fifth (17%) of migrant workers had experienced work related accidents[33]. Poor working environment and not being registered with a doctor was associated with a greater perceived health risk at the work place. Another study reported that over a quarter (27.9%) of migrant workers had experienced occupational injuries: more than half (52%) of these workers fell from a height, 21% had injuries due to fall of a heavy object, 17% had motor vehicle accident injuries, 5% had machinery injuries and remaining 5% had other work related injuries[16]. In a study conducted in Saudi Arabia, Nepalese migrant workers were the third-most unfit population to work; 1.6% were unfit due to the presence of infectious disease and 5.3% due to non-communicable disease[40]. Another study reported that more than one quarter (25.4%) of migrants had traffic

For peer review only - http://bmjopen.bmj.com/site/about/guidelines.xhtml

For peer review only

related pedestrian injuries during abroad work[21] (Table 1). A study by Pradhan et al conducted a retrospective analysis of Government of Nepal data from 2009–2017and recorded 1345 deaths, of which workplace accident and road traffic accidents contributed to 12% and 10% deaths respectively[36]. 33 cases of work-related ocular injuries were reported in one study among Nepalese patients of the 440 patients attending a hospital in Malaysia[26]. One qualitative study explored workplace accidents in GCC and Malaysia and reported several issues faced by the workers including lack of workplace safety, long working hours resulting in dehydration and heat stroke and injuries and accidents related issues including life-long disability[38].

Sexual Health

Only one moderate quality study in this review assessed the knowledge, attitudes and perceptions (KAP) of HIV/AIDS related risks[35]. The study was conducted among 408 adult Nepalese migrants (92% male) with at least six months of work experience in one of the three Gulf countries (Qatar, Saudi Arabia and UAE). The study showed that 91% of respondents had concerns about HIV/AIDS, and 17.2% of workers reported having sexual intercourse with a partner other than their spouse within the last 12 months. More than half (59%) of the respondents perceived themselves at high risk of being infected due to their sexual activities[35] (Table 1).

Mental Health

Five studies (all moderate quality) examined mental health issues among migrant workers. The sample of Nepalese migrants workers in these studies ranged between 20 to 1354[8 22 34 36 37]. One study on Nepalese female returnee migrant workers from Middle East and Malaysia reported the prevalence of mental health problems as 8.3%[8]. Another study reported that almost a quarter (23%) of labour migrants to Malaysia, Qatar and Saudi Arabia had experienced mental health issues, with a strong positive link between perceived health risk in the work environment and mental health status[34]. Third study reported a paradoxical finding with 4% increase in the predicted probability of depressive symptoms among Nepalese migrant workers compared to Arab, for every unit increase in perceived quality of life[22]. One study analysed Nepalese government's report and looked at 1354 deaths in Nepalese migrant workers, of which 8.5% were due to suicide[36]. The fifth quality qualitative study reported various mental health problems among the workers including loneliness, social isolation, tensions, anxiety, attempt to suicide[37] (Table 1).

For peer review only - http://bmjopen.bmj.com/site/about/guidelines.xhtml

Healthcare Access

Five studies (one high and four moderate quality) focused on labour migrants' healthcare access issues and the number of Nepalese workers in these studies ranged between 20 to 942 respectively [6 8 33 37 39]. Adhikari et al (2017) reported that workers who were not registered with a doctor had poor health outcomes compared to those who were registered[33]. Another study also reported that only 36.5% workers had access to health insurance and about half (48.7%) did not have paid sick leave during their health problems[6]. Another study on Nepalese female returnee migrant workers reported that only 11% of respondents received health services during their abroad work[8]. The fourth study reported that only insurance 68% of the workers had health insurance abroad and only 20% underwent regular health check-up[39]. In the qualitative study, participants reported poor access to mental health services related, mainly related to communication problems, and stigma to mental health[37] (Table 1).

Other Health Issues

A total of five studies (two high and three moderate quality) involving participants number ranging between 44 to 1354 reported various health issues [8 20 23 36 39]. One study on Nepalese female returnee migrant workers reported a prevalence of workplace abuse, torture or maltreatment at the workplace, and physical harm at 41%, 31% and 11%, respectively[8]. Clinical prevalence of oral lesions among migrant workers was found to be 4.6%[20]. Third study looked at the chronic kidney disease among workers and found that 13.6% of workers had diabetic nephropathy[39] . In the study by Pradhan et al, cardiovascular disease, natural/others reasons and murder contributed to 42%, 25% and 1.7% of deaths respectively[36] . The last study reported that of patients attending to the emergency medical service in Qatar, out-of-hospital cardiac arrest among Nepalese migrant patients was found to be 11.6%[20] (Table 1).

Studies on Infectious Diseases (Parasitic and Bacterial Infections, TB and Hepatitis E)

Of the 33 included studies, 17 studies (nine high and eight moderate quality) reported the proportion of sero-and/or faeco positive cases of infectious diseases (parasitic and bacterial Gastroenteric infections, tuberculosis, hepatitis E) [13-15 17 18 20 24 25 27-32 41-43]. The number of Nepalese workers included in these studies ranged between 12 to 1429. In several of these studies, Nepalese migrant workers had the higher proportion of infectious disease cases among the population studied. These infectious diseases included, toxoplasmosis (46.2%, working in

For peer review only - http://bmjopen.bmj.com/site/about/guidelines.xhtml

Malaysia) [25], tuberculosis (7%, Saudi Arabia and 11%, Qatar) [15 41], tuberculosis meningitis (37.5%, Qatar) [19], diarrhoeal bacterial infection (26.6%, Qatar)[17], protozoan ova/cysts (13.7%), helminths (6.2%), and hookworms (4.3%, Qatar) [13], Hepatitis E (74%, Qatar)[18], Brugian Lymphatic Filariasis (BmR1) (2.9%, Malaysia) and parasitic infection (BmSXP) (13%, Malaysia)[27]. Moreover, prevalence of salmonella among Nepalese migrant food handlers (3.7% Malaysia)[29], mean knowledge of food cleanliness and hygiene (73.1%, Malaysia) and symptom of foodborne illness (18.4% Malaysia)[31]. (Table 1).

Overall Quality Assessment

More than half of the cross-sectional prevalence studies (54% n=15/28) scored as 'high' quality and remaining were of moderate quality[8 13 16-18 20 27 29 31 36 39 41 42]. Similarly, three analytical studies were rated as moderate quality [22 33 34] and the two qualitative studies were rated as one high and one of moderate quality[37 38]. None of the studies were rated as poor quality. The results of the quality assessment scores are presented in Table 1 and details is presented in Appendix 2.

For peer review only

Table 1: Characteristics of studies included (n=33)

Author, Year, Country	Study Design and Setting	Participant Characteristics	Health Outcomes (Measurement Tools)	Key Findings	QA Scores
**Health risk and experiences related issues**					
**Dhakal et al, 2020, Nepal**	Hospital record data evaluated from the hospital data in Nepal (January – July, 2019)	Returnees migrant participants – 44<br>Gender – Male – 95% (n=42)<br>Age – Mean age 37.2 years	Healthcare access and prevalence of Chronic Kidney Disease (CKD)	- Workers with health insurance 68.2% (95% CI 52.4-81.3) (n-30)<br>- Underwent for routine health check-ups annually 20.4% (95% CI 9.8-35.3) (n-9)<br>- No regular health check-up 79.5% (95% CI 64.7-90.0) (n-35)<br>- Exposed to chemicals 27.3% (95% CI 14.9-42.7) (n-12)<br>- Patients were unknown about cause of CKD 77.3% (95% CI 62.1-88.5) (n-34)<br>- Had diabetic nephropathy .13.6% (95% CI 5.1-27.3) (n-6)<br>- Death due to kidney failure (n=1)	Moderate
**Khaled and Gray, 2019, Qatar**	Cross-sectional survey, February 2016	Migrant workers in Qatar<br>Total participants – 2520<br>Nepalese – 26% (n=655)<br>Gender-NR<br>Age - NR	Depressive symptoms	- Compared to Arabs, Nepalese migrant experienced 4%, increase in the predicted probability of depressive symptoms, for every unit increase in perceived quality of life.	Moderate
**Regmi et al, 2019, Nepal**	Qualitative Study (data collected in 2017)	Returnee migrants in Nepal from Qatar, Saudi Arab, Malaysia, Oman, UAE<br>Sampled - 20	Various health issues	- Unfair treatment and discrimination at work<br>- Poor working and living arrangements – dirty toilets and bathrooms<br>- Lack of security, loneliness and poor social life at work place/social isolation<br>- Mental health problems – tensions, anxiety and attempt to suicide and poor access to mental health services<br>- Poor communication facilities<br>- Only formality of pre-departure training package – contents good but poor implementation	Moderate
**Adhikary et al. 2019, Nepal**	Qualitative study (July to September, 2011)	Returnee migrants, interviews conducted interviews in Nepal – 20<br>Male – All<br>Mean age – 31.3 years	Workplace accidents among Nepali male workers in Qatar, Saudi Arabia and Malaysia.	Work place related issues:<br>- Not safe workplace<br>- High work pressure<br>- No medical supports from employer in host country<br>- Long working hours, mostly without timely food and drinking water resulting in dehydration and heat stroke<br>- Communication difficulty due to language barriers<br>- Injuries and accidents related issues	High

For peer review only

Author, Year, Country	Study Design and Setting	Participant Characteristics	Health Outcomes (Measurement Tools)	Key Findings	QA Scores
				- Fall from the roof, trapped in the hole<br>- Injured back bone, legs, hands and head<br>- Life-long disability	
**Pradhan et al, 2019, Nepal**	Retrospective analysis of Government of Nepal provided data (2009 – 2017)	Nepali migrant workers in Qatar<br>Total sample – 1354<br>Gender –NR<br>Age – NR	Analysed the deaths of Nepalese migrant workers	Causes of death due to:<br>- Cardiovascular –42% (95% CI 39.5-42.8) (n=571)<br>- Suicide – 8.5% (95% CI 7.1-10.1) (n=116)<br>- Workplace accident – 12.4% (95% CI 10.7-14.3) (n=169)<br>- Road traffic accident – 10.1% (95% CI 8.5-11.8) (n=137)<br>- Murder – 1.7% (95% CI 1.0-2.5) (n=23)<br>- Natural/others reasons for death – 25% (95% CI 22.6-27.3) (n=338)	Moderate
**Adhikary et al, 2018, Nepal**	Cross-sectional questionnaire-based survey	Male Nepalese construction workers, worked in host countries (Malaysia, Qatar and Saudi Arabia) for >6 months.<br>Total participants – 403<br>Age - NR	Self-reported health and wellbeing status	13.2% (95% CI 10.0-16.8) (n=53) reported poor/very poor health, relating to:<br>- Age older than 40 year reported as poor health (OR= 3.0, 95% 1.0-9.0)<br>- Poor work environment (OR= 6.8, 95% CI 3.2 – 14.6)<br>- Health risks at work (OR= 4.7, 95% CI 2.1-10.5)<br>- Prevalence of mental health issues was 23% overall - strong link between perceived health risks and mental health status.	Moderate
**Adhikary et al; 2017, Nepal**	Cross-sectional questionnaire-based survey	Male Nepalese construction and factory workers, worked >6 months in Malaysia, Qatar or Saudi Arabia.<br>Total participants – 423<br>Age -NR	Self-reported perceived health risks and accidents at work	Poor or very poor work environment (rated by the workers) associated with greater perceived health risk at work (OR 2.5, 95% CI=1.5-4.4)<br><br>Prevalence of accidents at work=17%<br>- Variables associated with accidents at work included: >Age 40 and above vs 20-29 (OR= 4.0, 95% CI=1.7-9.7)<br>- Not Satisfied accommodation vs satisfied with accommodation (OR=1.9, 95% CI=1.1-3.4)<br>- Poor or very poor work environment vs good/good to fair environment (OR 3.5, 95% CI=1.8-6.7)<br>- Working in Middle-East vs Malaysia. (OR .3.6, 95% CI=1.5-8.5)<br>Not registered with a doctor vs registered (OR=0.3, 95% CI=0.1-0.7)	Moderate
**Simkhada et al, 2017, Nepal**<br><br>***[Data for GCCs and Malaysia***	Retrospective analysis of NGO collected data (July 2009 to July 2014) via Paurakhi Nepal (NGO)	Returnee Nepalese female migrant workers from GCC [/Malaysia<br>(n= 942 = GCC = 933, Malaysia = 9)<br>Median age 31 (IQR 37)	Various health issues while working in GCC, middle-east and Malaysia (prevalence	Proportion female with health problems - 24% (95% CI 21.3-26.8) (n=226)<br>- Abuse at workplace -37% (95% CI 33.6-39.9) (n=346)<br>- Accident at workplace - 1.1% (95% CI 0.5-1.2) (n=10)<br>- Mental health problem - 8.3% (95% CI 6.6-10) (n=78)	Moderate

For peer review only

Author, Year, Country	Study Design and Setting	Participant Characteristics	Health Outcomes (Measurement Tools)	Key Findings	QA Scores
provided by the authors)		Age range – 14-51 years	calculated using information available from client Information Form/Sheet)	- Torture or maltreatment at the workplace 30.9% (95% CI 27.9-33.9-) (n=291)<br>- Pregnancy at work place - 3.1% (95% CI 2.1-4.3) (n=29)<br>- Sexual abuse – 51.7% (95% CI 32.5-70.5) (n =15/29)<br>- Physical harm -10.9% (95% CI 9.0-13.1) (n=103)<br><br>Received health services – 10.8% (95% CI 8.9-12.9) (n=102)	
**Irfan et al, 2016, Qatar**	Cross-Sectional study (June 2012-May 2013)	Patients attending to the emergency medical service in Qatar<br>Total participant – 447<br>Nepalese – 11.6% (n=52)<br>Gender-NR<br>Age – Median age 51 years (range 39-66 years)	Proportion of out of hospital cardiac arrest	Out-of-hospital cardiac arrest among Nepalese migrant patients – 11.6% (95% CI 8.8-14.9) (n=52). No further data.	High
**Min et al, 2016, Malaysia**	Retrospective cross-section of routine healthcare data (January 2011 to December 2013)	Patients attending to the eye casualty with work-related ocular injuries, in Hospital Sultan Ismail in Johor Bahru, Malaysia<br>Total 440 work-related ocular traumas.<br>Nepalese – 21.7% (n=33)<br>Gender –NR<br>Age - NR	Work related ocular traumas	33 cases of Nepalese work-related eye injuries. Causes range from open globe injuries due to being hit by a machine, nail, wood and metal whilst grinding.	High
**Al-Thani et al, 2015, Qatar**	Retrospective analysis of hospital trauma registry records 2010-2013 Hamad Trauma Centre	Total migrant participants - 2015<br>Nepalese –28% (n=563)<br>Male – 98% (n=1972)<br>Female –2% (n=43)<br>Age – NR	Proportion of occupational injuries and mortality cases	Overall proportion of occupational injury cases – 27.9% (n= 563), of which<br>- Falls from height – 52.4% (95% CI 48.1-56.5) (n=295)<br>- Fall of a heavy object – 20.4% (95% CI 17.1-24) (n= 115)<br>- Motor vehicle crashes injuries - 17% (95% CI 14.2-20.6) (n=97)<br>- Machinery injuries - 5% (95% CI 3.1-6.9) (n= 27)<br>- Others – 5% (95% CI 3.4-7.3) (n=29)	Moderate
**Latifi et al, 2015, Qatar**	Retrospective analysis of routine healthcare data	Total traffic related pedestrian injuries (TRPI) patients – 601<br>Total Nepalese expat TRPI patients – (n=147)<br>Gender –NR<br>Age – NR	Pedestrian morbidity and mortality	- 25.4% (95% CI 21.0-18.0) of TRPI were of Nepalese migrant workers (vs 16.0% of the general population of Qatar being Nepalese).<br>- 51.4% of TRPI with positive blood alcohol were Nepalese migrant workers.	High

For peer review only - http://bmjopen.bmj.com/site/about/guidelines.xhtml

Author, Year, Country	Study Design and Setting	Participant Characteristics	Health Outcomes (Measurement Tools)	Key Findings	QA Scores
**Joshi et al, 2014, Nepal**	Cross-sectional study	Nepalese migrants with experience of >6 months in Qatar, Saudi Arabia or United Arab Emirates.<br>Total participants – 408<br>Males – 92.4% (n=377)<br>Aged between 26-35 – 53.4% (n=218)	Knowledge of HIV/AIDS and risk perceptions	Risk perceptions of HIV/AIDS:<br>- Concerned about HIV/AIDS – 90% (95% CI 86.3-92.4) (n=366)<br>- Perceived themselves at high risk of being infected due to their sexual activities - 59.2% (n=397)<br>Sexual behaviour:<br>- 17.2% (95% CI 13.6-21.1) (n=70) had sexual intercourse with a partner other than their spouse during the last 12 months of their stay abroad.	Moderate
**Kavarodi et al, 2014, Qatar**	Population-based cross-sectional study	Low income expatriate workers from Indian sub-continent (living in Qatar for >6 months)<br>Total participants – 3,946<br>Nepalese – 5.4% (n=213)<br>Gender –NR<br>Age - NR	Clinical prevalence of suspected oral lesions.	- Oral Lesions in of Nepalese workers 4.7% (95% CI 2.1-7.8) (n=10).	High
**Alswaidi et al, 2013, Saudi Arabia**	Review of Ministry and Health data from Saudi expat worker fitness screening programme (1997–2010)	Total number of registered expatriate workers - 4 272 480<br>Nepalese – 0.9% (n=38 908).<br>Females – 14% (n=5 367)<br>Males – 86% (n=33 541)<br>Age – NR	Proportion of 'unfit' to workers.	Cases of unfitness among Nepalese workers by gender:<br>- Unfit males – 1.99% (95% CI 1.8-2.1) (n=669)<br>- Unfit females – 1.2% (95% CI 0.9-1.5) (n=64)<br>- Overall unfit – 1.9% (95% CI 1.7-2.0) (n=733)<br><br>Nepalese migrants were the third most unfit population.<br><br>Nepalese migrants as proportion of all those with:<br>- Infectious causes of unfitness (incl. hepatitis, HIV, TB) – 1.6% (n=379)<br>- Non-communicable causes of unfitness – 5.3% (n= 354)	High
**Joshi et al, 2011, Nepal**	Cross-sectional questionnaire survey, Kathmandu (International Airport and nearby hotels/lodges).	Returnee Nepalese male and female migrant workers from Qatar, Saudi Arabia and UAE (n=408)<br><br>Male = 377 (92.4%)<br>Female = 31 (7.6%)<br>Mean Age (SD) – 32 (6.5) years<br>Age ranges – 18-53 years	Prevalence of health problems using self-reported/ questionnaire survey	Prevalence of health problem(s) – 56.6% (95% CI 51.6-61.4) (n=231)<br>- Most common problems:<br>- Headache or fever - 30.7% (95% CI 24.8-37.1) (n=71)<br>- Respiratory symptoms - 21.2% (95% CI 16.1-27.0) (n=49)<br>- Musculoskeletal problems – 19.9% (95% CI 14.9-25.6) (n=46)<br>- Gastrointestinal illness – 19.5% (n=45)<br>- Injuries/poisoning – 13.9% (95% CI 9.6-18.9) (n=32)<br>Prevalence of some type of injury or accident at their workplace - 25% (95% CI 20.8-29.5) (n=102)	High

For peer review only

Author, Year, Country	Study Design and Setting	Participant Characteristics	Health Outcomes (Measurement Tools)	Key Findings	QA Scores
				- Health insurance in host countries- 36.5% (95% CI 31.8-41.4) (n=149)<br>- Sought health services or treatment in the working countries- 83.1% (95 % CI 42.1-51.0) (n=192)<br>- Lack of provision of leave during health problem(s)- 48.7% (n=19)	
**Infectious diseases related issues**					
**Al-Awadhi et al, 2019, Kuwait**	Retrospective analysis of routine healthcare data (2015 to 2017)	Migrant workers in Kuwait<br>Total examined participants – 1000<br>Nepalese – 3.3% (n=33)<br>Age - NR<br>Gender-NR	Prevalence of T solium by screening blood using a sensitive taeniasis-specific anti-rES33 antibody assay.	- 6.1% (95% CI 0.7-20.0) (n=2) of Nepalese migrant worker sample tested for T Solium taeniasis-specific lgG antibodies	High
**Sahimin et al, 2019 Malaysia**	Cross-sectional study (September 2014 – August 2015)	Migrant workers from manufacturing, services, agriculture and plantation, construction and domestic work sectors in Malaysia<br>Total participants - 610<br>Nepalese -(n=103)<br>Gender – NR<br>Age - NR	Measure prevalence of *E. dispar and E. histolytica*	- *E. dispar* 4.9% (95% CI 1.4-12.2)and *E. histolytica* infections 3.7% 95% CI (0.8-10.4)	High
**Sahimin et al, 2018, Malaysia**	Cross-sectional study	Migrant workers in Malaysia.<br>Total stool samples examined – 388<br>Nepalese –20.9% (81)<br>Gender –NR<br>Age - NR Gender –NR<br>Age - NR	Prevalence of Giardia duodenalis and Cryptosporidium parvum	- Giardia duodenalis 1.8% [0.7−3.7] and Cryptosporidium parvum 0.3 [0.0−1.4] respectively	High
**Dafalla et al, 2017, UAE**	Cross-sectional survey conducted at public health clinic	Immigrant workers – food handlers, babysitters, housemaids, drivers working in Sarjaha, UAE<br>Total participants– 21,347 (number of Nepalese workers not reported)<br>Total infected population –3.3% (n=708)<br>Gender –NR<br>Age – NR	Prevalence of parasitic infections (Examined microscopically and screened for intestinal parasites)	Proportion of infected migrant workers that are Nepalese – 6.2% (95% CI 4.5 – 8.2) (n=44)<br>- All protozoal infections: 7% (95% CI 5.9 – 8.6) (n=33)<br>- All helminth infections: 4.2% (95% CI 9.8 – 35.3) (n=9)	Moderate

For peer review only

Author, Year, Country	Study Design and Setting	Participant Characteristics	Health Outcomes (Measurement Tools)	Key Findings	QA Scores
**Noordin et al, 2017, Malaysia**	Cross-sectional survey (September 2014 to August 2015)	484 migrant workers from manufacturing, services, agriculture and plantation, construction and domestic work sectors.<br>Nepalese – 21.3% (n=103)<br>Gender –NR<br>Age – NR	Prevalence of parasitic infections	- Sero-prevalence of brugian Lymphatic Filariasis [BmR1] – 2.9% (95% CI 0.6 – 8.2) (n=3)<br>- Prevalence of parasitic infections (BmSXP) –12.6% (95% CI 6.8 – 20.6) (n=13)	Moderate
**Sahimin et al, 2017, Malaysia**	Correctional survey (Sept. 2014- Aug. 2015)	484 migrant workers<br>Nepalese respondents- 20.5% (n = 99)<br>Conducted at five working sectors (manufacturing, construction, plantation, domestic and food services)	Sero-prevalence T. gondii through Questionnaire survey and laboratory blood tests	Sero-prevalence:<br>- IgG – 74.7% (95% CI 65.0 – 82.9)<br>- lgM – 6.1% (95% CI 2.3 – 12.7)	High
**Woh et al, 2017, Malaysia**	Cross-sectional study	Healthy, asymptomatic migrant food handlers.<br>Total participants – 317<br>Nepalese – 25.2% (n=80)<br>Gender –NR<br>Age – NR	Prevalence of Salmonella carriers, using stool samples	- Prevalence of salmonella amongst Nepalese migrant food handlers – 3.7% (95% CI 0.7 – 10.5) (n=3)	Moderate
**Abu-Madi et al, 2016a, Qatar**	Retrospective analysis of routine healthcare data (2005 to 2014)	Records held at Hamad Medical Corporation data-base for subjects referred for stool examination<br>Total participants - 29,286<br>Nepalese – 4.8% (n=1429)<br>Gender –NR<br>Age - NR	Proportion of helminth infections positive cases.	- Highest proportion of helminth infections among Nepalese workers – 15.3 % (95% CI 13.39–17.12)	High
**Abu-Madi et al, 2016b, Qatar**	Retrospective analysis of routine healthcare data	Recently arrived migrant workers in Qatar<br>Total participants – 2,486<br>Nepalese – 15% (n=373)<br>Gender –NR<br>Age - NR	Presence of intestinal parasites (helminths and protozoa)	Proportion of positive cases in Nepalese migrant workers:<br>- Helminths combined - 6.2% (95% CI 3.8–9.6)<br>- Hookworms - 4.3% (95% CI 2.4–7.3)<br>Protozoa combined - 13.7% (95% CI 10.0–18.2)	Moderate
**Humphery et al, 2016, Qatar**	Community-based survey, Doha	Total participants– 126<br>Nepalese – 29.3% (n=37)<br>All male population<br>Median age (IQR) in years = 33 (27–39)	Prevalence of gastrointestinal pathogens (detected using polymerase chain reaction)	- Total prevalence of gastrointestinal pathogens = 62.7% (95% CI 53.6 – 71.1) (n=79)<br>- Gastrointestinal pathogens amongst Nepalese migrant workers – 26.6% (95% CI 10.6 – 24.3) (n=21)	Moderate

For peer review only - http://bmjopen.bmj.com/site/about/guidelines.xhtml

Author, Year, Country	Study Design and Setting	Participant Characteristics	Health Outcomes (Measurement Tools)	Key Findings	QA Scores
**Woh et al, 2016, Malaysia**	Cross-sectional survey (October 2014 to May 2015)	Migrant food handlers living in Malaysia<br>Total participants – 383<br>Nepalese – 24.8% (n-95)<br>Gender – NR<br>Age - NR	Knowledge and practices regarding the food handlings	Mean knowledge scores on:<br>- symptom of foodborne illness among Nepalese migrant – M = 18.4%, SD = 28.8<br>- food cleanliness and hygiene – M= 73.1%, SD = 15.3<br>Proportion of food handling practices among Nepalese migrant –<br>- Poor practices - 21.9% (n=7)<br>- Moderate – 14.3% (n=32)<br>- Good – 43.8% (n=56)	Moderate
**Imam et al, 2015, Qatar**	Retrospective analysis of routine healthcare data (January 2006 and December 2012)	Patients with suspected or confirmed tuberculous meningitis.<br>Total participants – 80<br>Nepalese – 37% (n=30)<br>Gender –NR<br>Age - NR	Clinical presentation, diagnosis, treatment, outcome, and the incidence of adult tuberculous meningitis	- 30/80 patients with tuberculous meningitis were Nepalese (37.5% (95% CI 26.9-49.0). No further data.	High
**Chattu and Mohammad, 2013, Saudi Arabia**	Retrospective analysis of routine healthcare data from Qassim region (January 2005 to December 2009)	Migrant workers (n= 165)<br>Male – 42% (n=70)<br>Female –58% (n=95)<br>Age - NR	Proportion of reported TB cases, using laboratory test	- Proportion of migrant workers with tuberculosis from Nepal: 7% (95% CI 3.8-12.3) (n=12).	Moderate
**Abu-Madi et al, 2011, Qatar**	Cross-sectional survey (June – September, 2009)	Patients resident in Qatar who were randomly recruited and conducted survey – 1538<br>Nepalese – 15.3% (n=236)<br>Gender – Male – 98.3% (n=232)<br>Female – 1.7% (n=4)<br>Age – mean age 28.2 years	Prevalence of intestinal parasitic infections among food handlers and housemaids)	Prevalence of all types of parasitic infections (species) – 29.7%, (95% CI 25.51 - 34.15)<br>- Helminths – 23.7%, (95% CI 19.91-27.98)<br>- Hookworms – 17.8%, (95% CI 14.40-21.73)<br>- A. lumbricoides – 2.5%, (95 CI (1.40-4.50)<br><br>Prevalence of all Protozoa – 9.7%, (95% CI 7.23-12.93)<br>- B. hominis – 3%, (95% CI 1.69-5.01)<br>- Prevalence of non-pathogenic:<br>- Amoebae – 3%, (95% CI (.69-5.01)<br>- G. duodenalis – 3.4%, (95% CI 2.02-5.52)	High
**Ibrahim et al, 2009, Qatar**	Community based survey, Alkhor hospital.	anti-HEV IgG Nepalese migrants nationally – 86<br>58 of these seen at Alkhor Hospital.<br>Gender –NR	Prevalence of Hepatitis E (using ELISA test) and other clinical symptoms	Prevalence of acute HEV amongst those seen at Alkhor Hospital – 74% (95% CI 60.9-84.7) (n=43)<br>- admitted to hospital – 95.3% (95% CI 84.1-99.4) (n=41)	Moderate

For peer review only

**Author, Year, Country**	**Study Design and Setting**	**Participant Characteristics**	**Health Outcomes (Measurement Tools)**	**Key Findings**	**QA Scores**
		Aged 26.7 (SD-5.6, range 19–41 years)			
**Chan et al, 2008, Malaysia**	Cross-sectional survey conducted in a plantation and detention camp of Malaysia	Total foreign migrant workers - 501<br>Nepalese – 5% (n=26)<br>Gender –NR<br>Age - NR	*Toxoplasma gondii* IgG and IgM sero-prevalence	- Prevalence of Toxoplasma gondii IgG – 46.2% (95% CI 26.5-66.6) (n=12)<br>- Prevalence of Toxoplasma gondii IgM – 11.5% (95% CI 2.4-30.0) (n=3)	High
**Al-Marri, 2001, Qatar**	Population-based retrospective analysis (January 1996 to December 1998)	Total cases of positive *M. tuberculosis* culture and sensitivity – 406<br>Nepalese migrant cases – 11% (n=44)<br>Gender –NR<br>Age - NR	Drug resistant cases of TB (where positive isolates identified)	- Of total 386 cases of pulmonary TB (321 expats) identified, 11% (95% CI 7.9-14.2) n= 44, Nepalese cases of TB, of which 9 cases were drug resistant.	High

For peer review only

For peer review only - http://bmjopen.bmj.com/site/about/guidelines.xhtml

Discussion

To our knowledge, this is the most comprehensive review of the health and well-being status/issues of the Nepalese migrant workers in the GCC countries and Malaysia. The resultant lack of disaggregated demographic data means that the overall characteristics of Nepalese participants is difficult to determine. The dissonance between issues covered in the peer-reviewed and grey literature for this population, namely in national and international media and in government reports, is notable. Disproportionately few studies focused on occupational mental, and sexual health of migrant workers.

Occupational Health

Our review identified seven papers focusing on occupational morbidity, mortality and fitness to work in the destination countries[16 21 26 33 36 38 40]. Only three of these focused solely on Nepalese migrants, and none compared occupation or working conditions with morbidity and mortality experienced[33 38 36]. This a crucial gap in the literature and further studies are needed to guide policy change. There has been widespread media coverage of the poor working conditions faced by Nepalese migrant workers and health impacts of these conditions are highlighted by the plight of manual labourers working for the forthcoming 2022 FIFA Qatar World Cup. Close to a fifth of labour migrants to Malaysia, Qatar and Saudi Arabia had experienced a workplace accident[33]. According to a Nepalese government report, there were circa7,467 deaths among Nepalese migrant workers abroad between 2008/09 and 2018/19, and over 40% of the deaths were deemed either of natural or other/unidentified cause[44]. Despite these workers being young (mean age 29 years) and fit (assessed by health screening both at home and destination countries), the magnitude of the proportion of these deaths is unusual in these groups[44]. This raises questions about robustness of post-mortem investigative practices and classification methodologies, a concern highlighted by both the Nepalese government and civil society groups[45]. Indeed, Pradhan et al suggest that many deaths attributed to cardiovascular diseases and 'natural causes' correlate with longer hours worked in high temperatures in this setting[36]. It is worth noting that Nepalese migrant workers themselves are not oblivious to these occupational risks- those who reported a poor or very poor work environment were found to be 3.5 times more likely to suffer a workplace accident[33].

For peer review only

Mental Health

Five studies in the review reported on mental health issues. Adhikari et al (2018) reported that almost a quarter of labour migrants to Malaysia, Qatar and Saudi Arabia had experienced mental health issues, with a strong positive correlation between perceived health risk in the work environment and mental health status[34]. The qualitative study by Regmi et al (2019) highlighted various mental health problems among the workers including loneliness, anxiety, and attempt to suicide[37]. Similar findings were reported in a cross-sectional study of 5000 migrant workers in Shanghai, where 21% reported mental disorders such as obsessive-compulsive disorder, anxiety, and hostility[46].

The Nepalese government report suggests that suicide is a significant cause of mortality in labour migrants to GCC countries and Malaysia, and there is evidence that mental health is an underexplored issue facing this population[47-49]. Only one of the study in this review looked at the suicide cases with nearly 10% of the deaths in these workers resulting from suicide[36]. The paucity of peer-reviewed studies exploring risk factors of poor mental status and psychiatric morbidity for this population requires urgent attention.

Migration for work is a time of significant turmoil: new language, new culture and poor working conditions. Loss of protective familial and wider social networks exacerbate feelings of homesickness, loneliness and hopelessness that commonly develop amongst this population[50-52]. Psychiatric under-diagnosis is common in deprived populations and is compounded by poor screening of those with pre-existing psychiatric conditions[53-55]. The result is lack of mental health support and omission of medications in destination contexts that can worsen conditions. Most common psychiatric morbidity in this population centred around depressive and anxiety-related disorders, although the impact of addiction particularly of alcohol consumption remains underexplored[49 56-59]. The impacts of labour migration on the mental health of left-behind families is also important, but beyond the scope of this review[47 60].

Sexual Health

Only a single study in this review examined sexual health issues amongst this population and exploring HIV/AIDS knowledge, attitudes and perceptions amongst Nepalese migrant workers. Joshi et al; (2014) reported that over 17% had had sexual intercourse with someone other than their spouse or partner during the final 12 months of their stay abroad[61]. This highlights higher levels of sexual risk taking behaviour, echoed by studies focusing on Nepalese migrants to India, which showed widespread use of local female sex-workers by male

For peer review only - http://bmjopen.bmj.com/site/about/guidelines.xhtml

Nepalese migrant populations, multiple sexual partners and low levels of condom use. Whilst there may be differences between the Indian and GCC or Malaysian contexts, the authors note there is a clear dearth of evidence around non-HIV/AIDS related sexual health of these migrants, and the impact of this on left-behind families[62 63]. Similar findings also revealed from the studies in Bangladesh and China among migrant workers at high risk of heterosexual HIV acquisition[64 65].

Infectious Disease

Out of 33 studies, 17 studies focused on migrant workers in a destination country and provided minimal disaggregated analysis on the Nepalese sub-population. Majority of these were done as a part of arrival screening and focus on infectious diseases were conducted from a destination country perspective. Overwhelmingly, the discussion sections of these studies focused on Nepalese migrant workers as potential vectors for transmitting infectious diseases to native population. This health security framing overlooks Nepalese labour migrants as a vulnerable population by virtue of their poor socioeconomic status in their origin country as well poor working and living conditions, and poor access to healthcare in destination countries[6 66 67]. Similar findings were also reported in a study from Singapore where a relatively high prevalence of malaria, hepatitis and tuberculosis was reported among migrant workers in Singapore[68]. Migrant workers in South Asia generally appear to have a greater prevalence of infectious diseases due to the complex interaction of several factors- this includes higher prevalence of infectious diseases in their native countries together with aforementioned poor access to healthcare and low socioeconomic status[7]. Acknowledgment and consequent introduction of policies to improve these structural drivers of infectious diseases amongst Nepalese migrants would be a more holistic approach that might both better protect the local population and improve the health and wellbeing of the vulnerable migrant population[69.]

Literature Gap for Female Migrant Workers

Women comprise only 7% of Nepalese labour migrant abroad[5]. However, the role of women in the migration story is far more significant and complex than this figure betrays with regards to true numbers of women migrating, roles of women ‘left behind’ and how it has influenced gender norms in Nepalese society. The complex interplay between various factors such as socio-cultural norms, women’s role in decision-making, and freedom to mobility reflect on their health from access to sexual and reproductive health services to gender-based violence[70]. Just one study has previously attempted to capture health outcomes among female migrants’[8].

For peer review only - http://bmjopen.bmj.com/site/about/guidelines.xhtml

They highlighted that almost a quarter of female Nepalese migrants faced multiple health problems and over 40% had faced workplace abuse, with close to half of the 3% that reported becoming pregnant whilst away doing so as a result of sexual abuse[8].

Female labour migration from Nepal has increased significantly over the past decade, driven by increasing demands in primarily GCC destination countries, poor agricultural employment opportunities and a slowly-changing gender norms[71]. One third of remittances to Nepal are from female migrant workers[8 72,]. Higher proportion (90%) of female labour migrants are undocumented workers in Gulf countries and this may have resulted from the restrictive governmental labour migration policies such as prohibition of women to work in the Gulf domestic sector[73]. Precarious channels of migration bring greater risks of exploitation and harm to health[74], yet neither the peer-reviewed literature in health, nor do wider literatures reflect the magnitude of these issues. More work is required on the health of Nepalese female migrants abroad, as well the challenges in reintegration that they face on their return[71].

Strengths and Limitations

This review has several strengths. As mentioned earlier, the review is the most comprehensive review to date on this population. As GCC and Malaysia are the most attractive destinations for migration, the findings of this review will have important research implications in terms of highlighting the research gap on specific health problems of migrant workers in general as well as the lack of research focus on female migrant workers. Also, the review will have important practical implications for informing health service delivery and making the services more culturally competent care for Nepalese workers. Secondly, not restricting studies based on particular health outcomes, peer reviewed studies looking at a range of health issues in this population were included. Screening of studies and quality assessment was conducted by two independent reviewers, ensuring low risk of selection bias in this review. We applied research design specific quality assessment tools, providing the accurate ratings of the articles. However, there were a number of limitations. The review did not systematically include grey literature although a number of key reports were used as reference points to compare to our findings from the peer-reviewed literature. The risk of missed studies by only searching English language databases is noted, particularly through exclusion of relevant Nepalese peer-reviewed journals. As there was heterogeneity in the outcome measured and the measurement tools used in the studies, we were unable to conduct meta-analysis. Also, recent guidelines have been published on reporting of narrative synthesis without meta-analysis[75], however these guidelines are more applicable for intervention studies, thus we have not used these in this

For peer review only

For peer review only - http://bmjopen.bmj.com/site/about/guidelines.xhtml

narrative systematic review. As the number of qualitative studies were very small (n=2), we reported the key findings from these studies rather than conducting a separate meta-synthesis.

Conclusion

This review identified a number of health issues among Nepalese migrant workers in the GCC countries and Malaysia, namely those centred on occupational, mental and sexual health of migrants, and infectious disease, together with health-related issues facing female labour migrants. Whilst there are early signs that Nepal may be moving beyond its predominantly remittance economy, there is no doubt that labour migration to Malaysia and the GCC countries is the reality facing an entire generation of working age Nepalese. The studies identified by the review highlight the need for improved health support, whether through regular health checks in destination countries, more stringent policies and legislation around permissible working conditions or better preparation for migration through more relevant pre-departure training. The findings suggest the urgent need to progressive policy changes, both in Nepal and destination countries, to better protect the health of labour migrants and improve their access to essential health services and acceptable working conditions.

Patient and public involvement This review was conducted as a part of a project to develop a culturally relevant intervention to support the health and wellbeing of Nepalese migrant workers in GCC countries. Migrants workers were involved throughout the project duration, including the formulation of research question for this systematic review.

Authors Contribution: PP and JC designed and supervised the study. PP wrote the review protocol, conducted the literature search, and wrote the final draft of the manuscript. SW and KK screened the articles, extracted the data, carried out quality assessment and contributed to the initial drafts. PP, JC and AM obtained funding for the study. JC, AM and PS reviewed and edited the manuscript. All authors read and approved the final manuscript.

Funding: This review was funded from Research England's institutional allocation from the Global Challenges Research Fund.

Conflict of Interest: None declared.

Data Availability Statement: All relevant data are provided in the manuscript or uploaded as supplementary information.

For peer review only

For peer review only - http://bmjopen.bmj.com/site/about/guidelines.xhtml

References

1. NPC. Sustainable development goals 2016–2030: National (preliminary) report. *Kathmandu: National Planning Commission* 2015
2. IOM. World migration report 2020: International Organization for Migration, 2019.
3. Khanal MN. Impact of male migration on contraceptive use, unmet need, and fertility in Nepal: Further analysis of the 2011 Nepal Demographic and Health Survey: Ministry of Health and Population 2013.
4. NDHS. Nepal Demographic and Health Survey 2016. Kathmandu: Ministry of Health and Population (MoHP) Nepal and ICF International Inc 2017.
5. MoLE. Labour migration for employment. A status report for Nepal: 2015/2016 – 2016/2017: Ministry of Labour and Employment Kathmandu, Nepal, 2018.
6. Joshi S, Simkhada P, Prescott GJ. Health problems of Nepalese migrants working in three Gulf countries. *BMC Int Health Hum Rights* 2011;11(3)
7. Mucci N, Traversini V, Giorgi G, et al. Migrant workers and physical health: An umbrella review. *Sustainability* 2019;11(1):232.
8. Simkhada P, Van Teijlingen E, Gurung M, et al. A survey of health problems of Nepalese female migrants workers in the Middle-East and Malaysia. *BMC Int Health Hum Rights* 2018;18(4)
9. Liberati A, Altman DG, Tetzlaff J, et al. The PRISMA statement for reporting systematic reviews and meta-analyses of studies that evaluate health care interventions: explanation and elaboration. *Journal of clinical epidemiology* 2009;62(10):e1-e34.
10. JBI. The Joanna Briggs Institute critical appraisal tools for use in JBI systematic reviews: checklist for prevalence studies. *Crit Apprais Checkl Preval Stud* 2017;7
11. Farsani SF, Brodovicz K, Soleymanlou N, et al. Incidence and prevalence of diabetic ketoacidosis (DKA) among adults with type 1 diabetes mellitus (T1D): a systematic literature review. *BMJ open* 2017;7(7):e016587.
12. Evans C, Tweheyo R, McGarry J, et al. Crossing cultural divides: A qualitative systematic review of factors influencing the provision of healthcare related to female genital mutilation from the perspective of health professionals. *PloS One* 2019;14(3):e0211829.
13. Abu-Madi MA, Behnke JM, Boughattas S, et al. Helminth infections among long-term-residents and settled immigrants in Qatar in the decade from 2005 to 2014: temporal trends and varying prevalence among subjects from different regional origins. *Parasites & Vectors* 2016;9(1):153.
14. Abu-Madi MA, Behnke JM, Ismail A, et al. Assessing the burden of intestinal parasites affecting newly arrived immigrants in Qatar. *Parasites and Vectors* 2016;9(619)
15. Al-Marri M. Pattern of mycobacterial resistance to four anti-tuberculosis drugs in pulmonary tuberculosis patients in the state of Qatar after the implementation of DOTS and a limited expatriate screening programme. *The Int J of TB and Lung Disease* 2001;5(12):1116-21.
16. Al-Thani H, El-Menyar A, Consunji R, et al. Epidemiology of occupational injuries by nationality in Qatar: evidence for focused occupational safety programmes. *Injury* 2015;46(9):1806-13.
17. Humphrey JM, Ranbhise S, Ibrahim E, et al. Multiplex polymerase chain reaction for detection of gastrointestinal pathogens in migrant workers in Qatar. *American J of Trop Med and Hygiene* 2016;95(6):1330-37.
18. Ibrahim AS, Alkhal A, Jacob J, et al. Hepatitis E in Qatar imported by expatriate workers from Nepal: Epidemiological characteristics and clinical manifestations. *J of Med Virology* 2009;81(6):1047-51.

For peer review only - http://bmjopen.bmj.com/site/about/guidelines.xhtml

19. Imam YZ, Ahmedullah HS, Akhtar N, et al. Adult tuberculous meningitis in Qatar: a descriptive retrospective study from its referral center. *European Neurology* 2015;73(1-2):90-97.
20. Kavarodi AM, Thomas M, Kannampilly J. Prevalence of oral pre-malignant lesions and its risk factors in an Indian subcontinent low income migrant group in Qatar. *Asian Pac J Cancer Prev* 2014;15(10):4325-9.
21. Latifi R, El-Menyar A, Al-Thani H, et al. Traffic-related pedestrian injuries amongst expatriate workers in Qatar: a need for cross-cultural injury prevention programme. *Int J of Injury Control and Safety Promotion* 2015;22(2):136-42.
22. Khaled SM, Gray R. Depression in migrant workers and nationals of Qatar: An exploratory cross-cultural study. *International Journal of Social Psychiatry* 2019;65(5):354-67.
23. Irfan FB, Bhutta ZA, Castren M, et al. Epidemiology and outcomes of out-of-hospital cardiac arrest in Qatar: A nationwide observational study. *International Journal of Cardiology* 2016;223:1007-13.
24. Abu-Madi MA, Behnke JM, Ismail A, et al. Comparison of intestinal parasitic infection in newly arrived and resident workers in Qatar. *Parasites & Vectors* 2011;4(1):211.
25. Chan B, Amal RN, Noor Hayati M, et al. Seroprevalence of toxoplasmosis among migrant workers from different Asian countries working in Malaysia. *Southeast Asian J of Trop Med and Public Health* 2008;39(1):9.
26. Min NN, Vasudevan SK, Jasman AA, et al. Work-related ocular injuries in Johor Bahru, Malaysia. *International Eye Science[Article]* 2016;16(3):416-22.
27. Noordin R, Mohd Zain SN, Yunus MH, et al. Seroprevalence of lymphatic filariasis among migrant workers in Peninsular Malaysia. *Transactions of The Royal Society of Trop Med and Hygiene* 2017;111(8):370-72.
28. Sahimin N, Lim YA, Ariffin F, et al. Socio-demographic determinants of Toxoplasma gondii seroprevalence in migrant workers of Peninsular Malaysia. *Parasites and Vectors* 2017;10(1):238.
29. Woh PY, Thong KL, Behnke JM, et al. Characterization of nontyphoidal Salmonella isolates from asymptomatic migrant food handlers in Peninsular Malaysia. *J Food Protection* 2017;80(8):1378-83.
30. Sahimin N, Lim YA, Noordin R, et al. Epidemiology and immunodiagnostics of Strongyloides stercoralis infections among migrant workers in Malaysia. *Asian Pacific Journal of Tropical Medicine* 2019;12(6):250.
31. Woh PY, Thong KL, Behnke JM, et al. Evaluation of basic knowledge on food safety and food handling practices amongst migrant food handlers in Peninsular Malaysia. *Food Control* 2016;70:64-73.
32. Sahimin N, Douadi B, Lim ALY, et al. Distribution of Giardia duodenalis (Assemblages A and B) and Cryptosporidium parvum amongst migrant workers in Peninsular Malaysia. *Acta Tropica* 2018;182:178-84.
33. Adhikary P, Sheppard ZA, Keen S, et al. Risky work: Accidents among Nepalese migrant workers in Malaysia, Qatar and Saudi Arabia. *Health Prospect* 2017;16(2):3-10.
34. Adhikary P, Sheppard ZA, Keen S, et al. Health and well-being of Nepalese migrant workers abroad. *Int J of Migration, Health and Social Care* 2018;14(1):96-105.
35. Joshi S, Prescott GJ, Simkhada P, et al. Knowledge and risk perceptions about HIV/AIDS among Nepalese Migrants in Gulf Countries: a cross-sectional study. *Health Science Journal* 2014;8(3):350-60.
36. Pradhan B, Kjellstrom T, Atar D, et al. Heat stress impacts on cardiac mortality in Nepali migrant workers in Qatar. *Cardiology* 2019;143(1):37-48.

For peer review only - http://bmjopen.bmj.com/site/about/guidelines.xhtml

37. Regmi PR, Van Teijlingen E, Mahato P, et al. The Health of Nepali Migrants in India: A Qualitative Study of Lifestyles and Risks. *International Journal of Environmental Research and Public Health* 2019;16(19):3655.

38. Adhikary P, Keen S, Van Teijlingen E. Workplace accidents among Nepali male workers in the Middle East and Malaysia: A qualitative study. *Journal of immigrant and minority health* 2019;21(5):1115-22.

39. Dhakal N, Shah D. SAT-136 Chronic kidney disease in migrant workers in Nepal. *Kidney International Reports* 2020;5(3):S58.

40. Alswaidi F, Memish Z, Al Hakeem R, et al. Saudi Arabian expatriate worker fitness-screening programme: a review of 14 years of data. *Eastern Medit Health J* 2013;19(7):664-70.

41. Chattu VK, Mohammad A. Tuberculosis an important global health issue in this era-a cross sectional study of epidemiology of TB among South Asian workers in Saudi Arabia. *Indian J Public Health* 2013;4:278.

42. Dafalla AIA, Almuhairi SASO, AlHosani MHJ, et al. Intestinal parasitic infections among expatriate workers in various occupations in Sharjah, United Arab Emirates. *Rev Inst Med Trop Sao Paulo* 2017;59(e82)

43. Al-Awadhi M, Iqbal J, Ahmad S. Cysticercosis, a Potential Public Health Concern in Kuwait: A New Diagnostic Method to Screen Taenia solium Taeniasis Carriers in the Expatriate Population. *Medical Principles and Practice* 2019 doi: DOI: 10.1159/000504625

44. MLESS. Nepal labour migration report 2020. Kathmandu, Nepal: Ministry of Labour Emplyment and Social Security, 2020.

45. Pattisson P. Majority of Nepal migrant deaths "should be treated as murder", Global development. The Guardian [Internet]. The Guardian. 2014 . https://www.theguardian.com/global-development/2014/may/20/nepal-migrant-deaths-treated-murder (21 October, 2019, date last accessed). 2014

46. Yang H, Gao J, Wang T, et al. Association between adverse mental health and an unhealthy lifestyle in rural-to-urban migrant workers in Shanghai. *J Formosan Medl Assoc* 2017;116(2):90-98.

47. JPAN. Migrant worker and mental health in Nepal. *J Psych Assoc of Nepal* 2014;1(1)

48. Poudel A. Mental health of migrant workers is a pressing issue, but it has been ignored [Internet]. https://kathmandupost.com/valley/2019/05/18/mental-health-of-migrant-workers-is-a-pressing-issue-but-it-has-been-ignored (21 Octomber, 2019, date last accessed), 2019.

49. Chapagai M, Pant S, Tulachan P, et al. Psychiatric morbidity among repatriated Nepalese foreign labor migrants-a hospital based study. *J Instit Med* 2017;41(1)

50. Maselko J. Social epidemiology and global mental health: expanding the evidence from high-income to low-and middle-income countries. *Current Epid Reports* 2017;4(2):166-73.

51. Weston G, Zilanawala A, Webb E, et al. Long work hours, weekend working and depressive symptoms in men and women: findings from a UK population-based study. *J Epid Com Health* 2019;73(5):465-74.

52. Donini A. Social suffering and structural violence: Nepali workers in Qatar. *Int Dev Policy* 2019:178-99. http://journals.openedition.org/poldev/3077 (24 October, 2019, date last accessed).

53. Murphy JM, Olivier DC, Monson RR, et al. Depression and anxiety in relation to social status: A prospective epidemiologic study. *Archives of General Psychiatry* 1991;48(3):223-29.

For peer review only - http://bmjopen.bmj.com/site/about/guidelines.xhtml

54. Lao CK, Chan YM, Tong HHY, et al. Underdiagnosis of depression in an economically deprived population in Macao, China. *Asia-Pacific Psychiatry* 2016;8(1):70-79.
55. Pulkki-Råback L, Ahola K, Elovainio M, et al. Socio-economic position and mental disorders in a working-age Finnish population: the health 2000 study. *The European J of Public Health* 2011;22(3):327-32.
56. Pocock NS, Chan Z, Loganathan T, et al. Moving towards culturally competent health systems for migrants? Applying systems thinking in a qualitative study in Malaysia and Thailand. *PloS One* 2020;15(4):e0231154.
57. Poudel KC, Jimba M, Okumura J, et al. Migrants' risky sexual behaviours in India and at home in far western Nepal. *J Trop Med Hyg* 2004;9(8):897-903.
58. Bam K, Thapa R, Newman MS, et al. Sexual behavior and condom use among seasonal Dalit migrant laborers to India from Far West, Nepal: a qualitative study. *PLoS One* 2013;8(9):e74903.
59. Simkhada PP, Regmi PR, Van Teijlingen E, et al. Identifying the gaps in Nepalese migrant workers' health and well-being: a review of the literature. *J of Travel Med* 2017;24(4)
60. Aryal N, Regmi PR, van Teijlingen E, et al. Adolescents left behind by migrant workers: a call for community-based mental health interventions in Nepal. *WHO South-East Asia J of Public Health* 2019;8(1):38-41.
61. Al-Maniri A, Fochsen G, Al-Rawas O, et al. Immigrants and health system challenges to TB control in Oman. *BMC Health Services Research* 2010;10:210.
62. Aryal N, Regmi P, Teijlingen E, et al. Knowing is not enough: migrant workers' spouses vulnerability to HIV. *SAARC J TB, Lung Diseases and HIV/AIDS* 2016;13(1):9-15.
63. Thapa S, Bista N, Hannes K, et al. Vulnerability of wives of Nepalese labor migrants to HIV infection: integrating quantitative and qualitative evidence. *Women and Health* 2016;56(7):745-66.
64. Urmi AZ, Leung DT, Wilkinson V, et al. Profile of an HIV testing and counseling unit in Bangladesh: majority of new diagnoses among returning migrant workers and spouses. *PloS One* 2015;10(10):e0141483.
65. Ning C, Jiang J, Ye L, et al. Comparison of three intervention models for promoting circumcision among migrant workers in western China to reduce local sexual transmission of HIV. *PloS one* 2013;8(9):e76107.
66. Seddon D, Adhikari J, Gurung G. Foreign labor migration and the remittance economy of Nepal. *Critical Asian Studies* 2002;34(1):19-40.
67. Bhandari P. Relative deprivation and migration in an agricultural setting of Nepal. *Popn and Envt* 2004;25(5):475-99.
68. Sadarangani SP, Lim PL, Vasoo S. Infectious diseases and migrant worker health in Singapore: a receiving country's perspective. *J of Travel Med* 2017;24(4)
69. Castelli F, Sulis G. Migration and infectious diseases. *Clinical Microb Infect* 2017;23(5):283-89.
70. Colombini M, Mayhew SH, Hawkins B, et al. Agenda setting and framing of gender-based violence in Nepal: how it became a health issue. *Health Policy and Planning* 2015;31(4):493-503.
71. Gioli G, Maharajan A, M G. Neither heroines nor victims: Women migrant workers and changing family and community relations in Nepal [Internet]. 2017. https://www.refworld.org/pdfid/5a1bf0374.pdf (21 October, 2019, date last accessed).2017.
72. TheWorldBank. Migration and remittances factbook 2016 advanced edition [Internet]. 2016. https://siteresources.worldbank.org/INTPROSPECTS/Resources/334934-1199807908806/4549025-1450455807487/Factbookpart1.pdf (21 October, 2019, date last accessed) 2016

For peer review only - http://bmjopen.bmj.com/site/about/guidelines.xhtml

73. WOREC. Women and migration. [Internet] 2012. https://issuu.com/worecnepal/docs/migration-and-women (24 Nov, 2019, date last accessed)2012.
74. Pyakurel UP. Restrictive labour migration policy on Nepalese women and consequences. *Sociology and Anthropology* 2018;6(8):650-56.
75. Campbell M, McKenzie JE, Sowden A, et al. Synthesis without meta-analysis (SWiM) in systematic reviews: reporting guideline. *BMJ* 2020;368

For peer review only

For peer review only - http://bmjopen.bmj.com/site/about/guidelines.xhtml

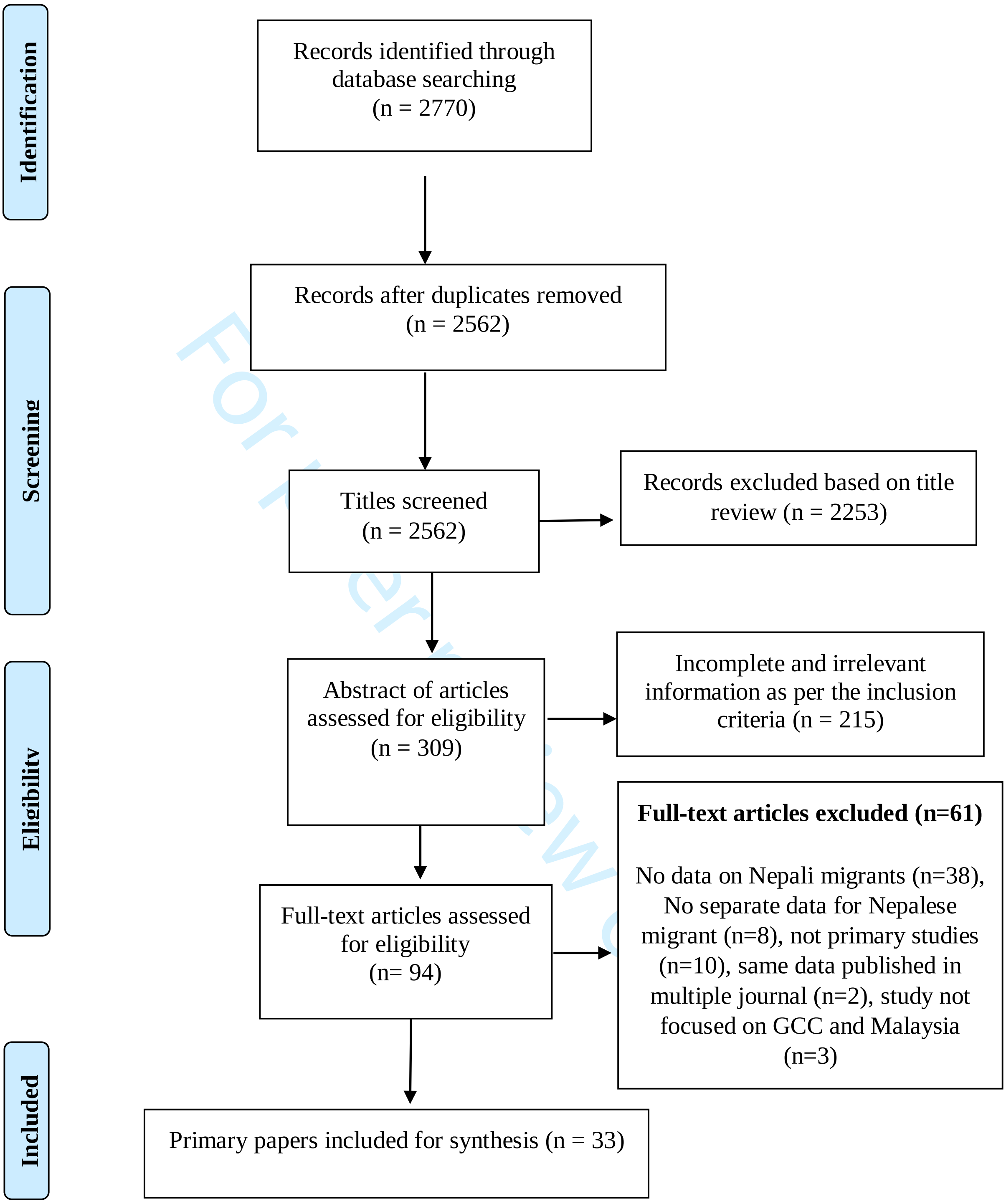

Figure 1: PRISMA Flow Diagram of Study Selection

Appendix 1 Keywords used for search in MEDLINE

1. Migration {Including Related Terms}
2. Migrant {Including Related Terms}
3. Emigrant {Including Related Terms}
4. Immigrant{Including Related Terms}
5. Expatriate {Including Related Terms}
6. Foreign worker {Including Related Terms}
7. Labor migration {Including Related Terms}
8. Left-behind {Including Related Terms}
9. Migrant families {Including Related Terms}
10. Or/1-9
11. Nepal {Including Related Terms}
12. Nepalese {Including Related Terms}
13. Nepali {Including Related Terms}
14. UAE or United Araba Emirates {Including Related Terms}
15. GCC or Gulf Cooperating council {Including Related Terms}
16. Middle East {Including Related Terms}
17. Bahrain {Including Related Terms}
18. Saudi Arabia {Including Related Terms}
19. Oman {Including Related Terms}
20. Qatar {Including Related Terms}
21. Kuwait {Including Related Terms}
22. Malaysia {Including Related Terms}
23. Or/11-22
24. 10 AND 23

For peer review only

For peer review only

Appendix 2:

Quality Assessment of Quantitative Studies (Prevalence Surveys)

Reference	Was the sample frame appropriate to address the target population?	Were study participants sampled in an appropriate way?	Was the sample size adequate?	Were the study subjects and the setting described in detail?	Was the data analysis conducted with sufficient coverage of the identified sample?	Were valid methods used for the identification of the condition?	Was the condition measured in a standard, reliable way for all participants?	Was there appropriate statistical analysis?	Was the response rate adequate, and if not, was the low response rate managed appropriately?	Scores	Overall quality
Overview N-28 studies	Yes - 27 (96.5%)	Yes - 20 (71.4%)	Yes - 17 (60.7%)	Yes – 25 (89.3%)	Yes – 26 (92.9%)	Yes – 26 (92.9%)	Yes – 25 (89.3%)	Yes – 25 (89.3%)	Yes – 15 (53.6%)		H- 15 (53.6%)
	Unclear - 1 (3.5%)	Unclear- 7 (25.0%)	Unclear– 11 (39.3%)	Unclear – 3 (10.7%)	Unclear – 2 (7.1%)	Unclear – 2 (7.1%)	Unclear – 3 (10.7%)	Unclear – 3 (10.7%)	Unclear – 13 (46.4%)		M- 13 (46.4%)
	No - 0	No - 1 (3.6%)	No - 0	No – 0	No - 0	No - 0	No - 0	No - 0	No - 0		
Abu-Madi et al, 2016a	Yes	Yes	Yes	Yes	Yes	Yes	Yes	Yes	Yes	9	High
Abu-Madi et al, 2016b	Yes	Yes	Yes	Unclear	Yes	Yes	Yes	Unclear	Yes	7	Moderate
Abu-Madi et al, 2011	Yes	Yes	Unclear	Yes	Yes	Yes	Yes	Yes	Yes	8	High
Al-Awadhi et al, 2019	Yes	Yes	Yes	Yes	Yes	Yes	Yes	Yes	Unclear	8	High
Al-Marri et al, 2001	Yes	Yes	Yes	Yes	Yes	Yes	Yes	Unclear	Yes	8	High
Alswaidi et al, 2013	Yes	Yes	Yes	Yes	Yes	Yes	Yes	Yes	Yes	9	High
Al-Thani et al, 2015	Yes	Unclear	Yes	Yes	Yes	Yes	Unclear	Yes	Yes	7	Moderate
Chan et al, 2008	Unclear	Yes	Yes	Yes	Yes	Yes	Yes	Yes	Yes	8	High
Chattu et al, 2013	Yes	Yes	Unclear	Yes	Unclear	Yes	Unclear	Yes	Unclear	5	Moderate
Dafalla et al, 2017	Yes	Unclear	Yes	Yes	Yes	Yes	Yes	Yes	Unclear	7	Moderate
Dhakal et al, 2020	Yes	Yes	Unclear	Yes	Yes	Unclear	Yes	Yes	Unclear	6	Moderate
Humphery et al, 2016	Yes	Unclear	Unclear	Yes	Yes	Yes	Yes	Yes	Unclear	6	Moderate
Ibrahim et al, 2009	Yes	Yes	Unclear	Yes	Unclear	Yes	Yes	Yes	Unclear	6	Moderate
Imam et al, 2015	Yes	Yes	Unclear	Yes	Yes	Yes	Yes	Yes	Yes	8	High
Irfan et al, 2016	Yes	Yes	Yes	Yes	Yes	Yes	Yes	Yes	Unclear	8	High

For peer review only

Reference	Was the sample frame appropriate to address the target population?	Were study participants sampled in an appropriate way?	Was the sample size adequate?	Were the study subjects and the setting described in detail?	Was the data analysis conducted with sufficient cover age of the identified sample?	Were valid methods used for the identification of the condition?	Was the condition measured in a standard, reliable way for all participants?	Was there appropriate statistical analysis?	Was the response rate adequate, and if not, was the low response rate managed appropriately?	Scores	Overall quality
Joshi et al, 2011	Yes	Yes	Yes	Yes	Yes	Yes	Yes	Yes	Yes	9	High
Joshi et al, 2014	Yes	No	Yes	Yes	Yes	Unclear	Yes	Unclear	Yes	7	Moderate
Kavarodi et al, 2014	Yes	Yes	Yes	Yes	Yes	Yes	Yes	Yes	Yes	9	High
Latifi et al, 2015	Yes	Yes	Yes	Unclear	Yes	Yes	Yes	Yes	Yes	8	High
Min, 2016	Yes	Yes	Unclear	Yes	Yes	Yes	Yes	Yes	Yes	8	High
Noordin et al, 2017	Yes	Yes	Unclear	Unclear	Yes	Yes	Yes	No	Unclear	5	Moderate
Pradhan et al, 2019	Yes	Unclear	Unclear	Yes	Yes	Yes	Unclear	Yes	Unclear	5	Moderate
Sahimin et al, 2019	Yes	Yes	Yes	Yes	Yes	Yes	Yes	Yes	Unclear	8	High
Sahimin et al, 2018	Yes	Yes	Yes	Yes	Yes	Yes	Yes	Yes	Yes	9	High
Sahimin et al, 2017	Yes	Yes	Yes	Yes	Yes	Yes	Yes	Yes	Yes	9	High
Simkhada et al, 2017	Yes	Unclear	Unclear	Yes	Yes	Yes	Yes	Yes	Unclear	6	Moderate
Woh et al, 2016	Yes	Unclear	Unclear	Yes	Yes	Yes	Yes	Yes	Unclear	6	Moderate
Woh et al, 2017	Yes	Unclear	Unclear	Yes	Yes	Yes	Yes	Yes	Yes	7	Moderate

For peer review only

Quality Assessment of Quantitative Studies (Analytical Cross-sectional Surveys)

Reference (n=3 studies)	Were the criteria for inclusion in the sample clearly defined?	Were the study subjects and the setting described in detail?	Was the exposure measured in a valid and reliable way?	Were objective, standard criteria used for measurement of the condition?	Were confounding factors identified?	Were strategies to deal with confounding factors stated?	Were the outcomes measured in a valid and reliable way?	Was appropriate statistical analysis used?	Score	Overall Quality
Adhikary et al, 2017	Yes	Yes	Yes	Yes	No	No	Yes	Yes	6	Moderate
Adhikary et al, 2018	Yes	Yes	Yes	Yes	No	No	Yes	Yes	6	Moderate
Khaled and Gray, 2019	Yes	Yes	Yes	Yes	No	No	Yes	Yes	6	Moderate

Quality Assessment of the Qualitative Studies

Reference (n=2 studies)	Is there congruity between the stated philosophical perspective and the research methodology?	Is there congruity between the research methodology and the research question or objectives?	Is there congruity between the research methodology and the methods used to collect data?	Is there congruity between the research methodology and the representation and analysis of data?	Is there congruity between the research methodology and the interpretation of results?	Is there a statement locating the researcher culturally or theoretically?	Is the influence of the researcher on the research, and vice- versa, addressed?	Are participants, and their voices, adequately represented?	Is the research ethical according to current criteria or, for recent studies, and is there evidence of ethical approval by an appropriate body?	Do the conclusions drawn in the research report flow from the analysis, or interpretation, of the data?	Score	Overall Quality
Adhikary et al, 2019	Yes	Yes	Yes	Yes	Yes	Unclear	Unclear	Yes	Yes	Yes	8	High
Regmi et al, 2019	Yes	Yes	Yes	Unclear	Yes	No	Unclear	Yes	Yes	Yes	7	Moderate

For peer review only

PRISMA 2009 Checklist

Section/topic	#	Checklist item	Reported on page #
**TITLE**			
Title	1	Identify the report as a systematic review, meta-analysis, or both.	1
**ABSTRACT**			
Structured summary	2	Provide a structured summary including, as applicable: background; objectives; data sources; study eligibility criteria, participants, and interventions; study appraisal and synthesis methods; results; limitations; conclusions and implications of key findings; systematic review registration number.	2
**INTRODUCTION**			
Rationale	3	Describe the rationale for the review in the context of what is already known.	3
Objectives	4	Provide an explicit statement of questions being addressed with reference to participants, interventions, comparisons, outcomes, and study design (PICOS).	3
**METHODS**			
Protocol and registration	5	Indicate if a review protocol exists, if and where it can be accessed (e.g., Web address), and, if available, provide registration information including registration number.	4
Eligibility criteria	6	Specify study characteristics (e.g., PICOS, length of follow-up) and report characteristics (e.g., years considered, language, publication status) used as criteria for eligibility, giving rationale.	4
Information sources	7	Describe all information sources (e.g., databases with dates of coverage, contact with study authors to identify additional studies) in the search and date last searched.	4
Search	8	Present full electronic search strategy for at least one database, including any limits used, such that it could be repeated.	Appendix 1
Study selection	9	State the process for selecting studies (i.e., screening, eligibility, included in systematic review, and, if applicable, included in the meta-analysis).	4,5
Data collection process	10	Describe method of data extraction from reports (e.g., piloted forms, independently, in duplicate) and any processes for obtaining and confirming data from investigators.	5
Data items	11	List and define all variables for which data were sought (e.g., PICOS, funding sources) and any assumptions and simplifications made.	5
Risk of bias in individual studies	12	Describe methods used for assessing risk of bias of individual studies (including specification of whether this was done at the study or outcome level), and how this information is to be used in any data synthesis.	5
Summary measures	13	State the principal summary measures (e.g., risk ratio, difference in means).	5
Synthesis of results	14	Describe the methods of handling data and combining results of studies, if done, including measures of consistency (e.g., I^2) for each meta-analysis.	5

For peer review only - http://bmjopen.bmj.com/site/about/guidelines.xhtml

PRISMA 2009 Checklist

Section/topic	#	Checklist item	Reported on page #
Risk of bias across studies	15	Specify any assessment of risk of bias that may affect the cumulative evidence (e.g., publication bias, selective reporting within studies).	5
Additional analyses	16	Describe methods of additional analyses (e.g., sensitivity or subgroup analyses, meta-regression), if done, indicating which were pre-specified.	NA
**RESULTS**			
Study selection	17	Give numbers of studies screened, assessed for eligibility, and included in the review, with reasons for exclusions at each stage, ideally with a flow diagram.	6
Study characteristics	18	For each study, present characteristics for which data were extracted (e.g., study size, PICOS, follow-up period) and provide the citations.	8-19
Risk of bias within studies	19	Present data on risk of bias of each study and, if available, any outcome level assessment (see item 12).	Appendix 2
Results of individual studies	20	For all outcomes considered (benefits or harms), present, for each study: (a) simple summary data for each intervention group (b) effect estimates and confidence intervals, ideally with a forest plot.	Table 1
Synthesis of results	21	Present results of each meta-analysis done, including confidence intervals and measures of consistency.	NA
Risk of bias across studies	22	Present results of any assessment of risk of bias across studies (see Item 15).	Appendix 2
Additional analysis	23	Give results of additional analyses, if done (e.g., sensitivity or subgroup analyses, meta-regression [see Item 16]).	NA
**DISCUSSION**			
Summary of evidence	24	Summarize the main findings including the strength of evidence for each main outcome; consider their relevance to key groups (e.g., healthcare providers, users, and policy makers).	20
Limitations	25	Discuss limitations at study and outcome level (e.g., risk of bias), and at review-level (e.g., incomplete retrieval of identified research, reporting bias).	23
Conclusions	26	Provide a general interpretation of the results in the context of other evidence, and implications for future research.	24
**FUNDING**			
Funding	27	Describe sources of funding for the systematic review and other support (e.g., supply of data); role of funders for the systematic review.	24

From: Moher D, Liberati A, Tetzlaff J, Altman DG, The PRISMA Group (2009). Preferred Reporting Items for Systematic Reviews and Meta-Analyses: The PRISMA Statement. PLoS Med 6(7): e1000097. doi:10.1371/journal.pmed1000097

For peer review only - http://bmjopen.bmj.com/site/about/guidelines.xhtml

BMJ Open

Health and Wellbeing Issues of Nepalese Migrant Workers in the Gulf Cooperation Council Countries and Malaysia: A Systematic Review

Journal:	*BMJ Open*
Manuscript ID	bmjopen-2020-038439.R2
Article Type:	Original research
Date Submitted by the Author:	25-Aug-2020
Complete List of Authors:	Paudyal, Priyamvada; Brighton and Sussex Medical School Division of Primary Care and Public Health Medicine<br>Kulasabanathan, Kavian ; Brighton and Sussex Medical School Division of Primary Care and Public Health Medicine<br>Cassell, Jackie; Brighton and Sussex Medical School Division of Primary Care and Public Health Medicine<br>Memon, Anjum; Brighton and Sussex Medical School Division of Primary Care and Public Health Medicine<br>Simkhada, Padam; University of Huddersfield, School of Human and Health Sciences<br>Wasti, Sharada; Green Tara Nepal
**Primary Subject Heading**:	Global health
Secondary Subject Heading:	Public health
Keywords:	PUBLIC HEALTH, EPIDEMIOLOGY, Health policy < HEALTH SERVICES ADMINISTRATION & MANAGEMENT

SCHOLARONE™
Manuscripts

I, the Submitting Author has the right to grant and does grant on behalf of all authors of the Work (as defined in the below author licence), an exclusive licence and/or a non-exclusive licence for contributions from authors who are: i) UK Crown employees; ii) where BMJ has agreed a CC-BY licence shall apply, and/or iii) in accordance with the terms applicable for US Federal Government officers or employees acting as part of their official duties; on a worldwide, perpetual, irrevocable, royalty-free basis to BMJ Publishing Group Ltd ("BMJ") its licensees and where the relevant Journal is co-owned by BMJ to the co-owners of the Journal, to publish the Work in this journal and any other BMJ products and to exploit all rights, as set out in our licence.

The Submitting Author accepts and understands that any supply made under these terms is made by BMJ to the Submitting Author unless you are acting as an employee on behalf of your employer or a postgraduate student of an affiliated institution which is paying any applicable article publishing charge ("APC") for Open Access articles. Where the Submitting Author wishes to make the Work available on an Open Access basis (and intends to pay the relevant APC), the terms of reuse of such Open Access shall be governed by a Creative Commons licence – details of these licences and which Creative Commons licence will apply to this Work are set out in our licence referred to above.

Other than as permitted in any relevant BMJ Author's Self Archiving Policies, I confirm this Work has not been accepted for publication elsewhere, is not being considered for publication elsewhere and does not duplicate material already published. I confirm all authors consent to publication of this Work and authorise the granting of this licence.

For peer review only

Health and Wellbeing Issues of Nepalese Migrant Workers in the Gulf Cooperation Council Countries and Malaysia: A Systematic Review

Priyamvada Paudyal[1], Kavian Kulasabanathan[1], Jackie Cassell[1], Anjum Memon[1], Padam Simkhada[2], Sharada Prasad Wasti[3]

[1]Department of Primary Care and Public Health, Brighton and Sussex Medical School, Brighton UK; [2]Faculty of Health, University of Huddersfield, UK; [3]Green Tara Nepal, Kathmandu, Nepal

Corresponding Author: Dr Priyamvada Paudyal
Department of Primary Care and Public Health, Brighton and Sussex Medical School, Room 322, Watson Building, Village Way, Falmer, BRIGHTON, BN1 9PH, UK
+44 (0) 1273 644548; p.paudyal@bsms.ac.uk

Running Title: Health issues of Nepalese migrant workers in Gulf Countries and Malaysia

For peer review only

For peer review only - http://bmjopen.bmj.com/site/about/guidelines.xhtml

Abstract:

Objective: To summarise the evidence on health and wellbeing of Nepalese migrant workers in the Gulf Cooperation Council (GCC) countries and Malaysia

Design: Systematic Review

Data Sources: EMBASE, MEDLINE, Scopus and Global Health databases

Eligibility Criteria: Studies were eligible if they: 1) included Nepalese migrant workers aged 18 or older working in the GCC countries or Malaysia or returnee migrant workers from these countries; 2) were primary studies that investigated health and wellbeing status/issues; and 3) were published in English language before 8 May 2020.

Study Appraisal: All included studies were critically appraised using Joanna Briggs Institute study specific tools.

Results: A total of 33 studies were eligible for inclusion; 12 studies were conducted in Qatar, eight in Malaysia, nine in Nepal, two in Saudi Arabia and one each in UAE and Kuwait. In majority of the studies, there was a lack of disaggregated data on demographic characteristics of Nepalese migrant workers. Nearly half of the studies (n=16) scored as 'high' quality and the rest (n=17) as 'moderate' quality. Five key health and wellbeing related issues were identified in this population: a) occupational hazards; b) sexual health; c) mental health; d) healthcare access and e) infectious diseases.

Conclusion: To our knowledge, this is the most comprehensive review of the health and well-being of Nepalese migrant workers in the GCC countries and Malaysia. This review highlights an urgent need to identify and implement policies and practices across Nepal and destination countries to protect the health and wellbeing of migrant workers.

Protocol Registration: The review protocol was developed and registered on the University of Sussex website

Key Terms: Migration, GCC countries, Health, Wellness, Nepalese

For peer review only - http://bmjopen.bmj.com/site/about/guidelines.xhtml

Word Limit 300

Word Count: 264

Strengths and Limitations

- This review is the most comprehensive review to date on this population.
- The review did not restrict studies based on particular health outcomes, peer reviewed studies looking at a range of health issues in this population were included.
- Meta-analysis was not conducted as there was heterogeneity in the outcome measured and the measurement tools used in the studies.

For peer review only

Introduction

Migration is the overarching narrative of our time, and its impact is increasingly being recognised in global public health agendas. The United Nations (UN) Sustainable Development Goals (SDGs) identify migration as a catalyst for development and recommend that 'no-one should be left behind' to achieve Universal Health Coverage for all[1]. According to the World Migration Report 2020, the number of international migrants has reached approximately 272 million, and two third of these are estimated to be labour migrants[2]. Labour migration has been a key determinant of population changes in Asia, especially in Gulf Cooperation Council (GCC) countries, a major destination for workers within Asia[2].

Nepal is a low-income country going through a demographic transition, with an ageing population and attendant chronic diseases. According to the Nepal Migration Report 2020, over four million labour approval were issued to Nepalese workers in the last decade since 2008/2009[3]. The Nepal Demographic and Health Survey (2016) reported that nearly half (47%) the households have at least one family member who migrated in the last 10 years either in internal or international destinations[4]. These migrant workers contribute over a quarter of the country's gross domestic product (GDP) through remittance from abroad. The migration outflow consists predominantly of low-skilled male workers, primarily to Malaysia and the GCC countries[3].

Labour migration contributes significantly to the sociocultural and economic development of both origin and destination countries. However, migrant workers experience specific vulnerabilities, and face a range of health risks while working abroad. These risks are particularly significant for Nepalese workers in the GCC countries, as they are often employed in occupations considered 'difficult, dirty, and dangerous (3Ds)'. These are sectors with higher occupational risks such as agriculture, construction, transport and heavy industry. Furthermore, Nepalese migrant workers consistently work for longer hours as compared to native workers[5 6] and are often exposed to factors which promote poor health and wellbeing, including low wages, poor housing, an unhealthy diet, and difficulty in accessing health services[5, 7]. Many Nepalese migrant workers die abroad every year including a significant number that are unexplained, while a large number return home with debilitating injuries, and both mental and physical illness[5]. This systematic review identified and summarised the evidence from primary studies on the health and wellbeing of Nepalese migrant workers in the GCC countries and

For peer review only - http://bmjopen.bmj.com/site/about/guidelines.xhtml

Malaysia, the destination countries for 88% of labour migration. This review was conducted as a part of University of Sussex internally funded Global Challenges Research Fund (GCRF) project to develop a culturally relevant intervention to support the health and wellbeing of Nepalese migrant workers in GCC countries.

Methods

Protocol Registration

This study protocol was registered at the University of Sussex (http://sro.sussex.ac.uk/id/eprint/86400/). The study followed the PRISMA (Preferred Reporting Items for Systematic Reviews and Meta-Analyses) guidelines and recommendations of the Cochrane Collaboration (www.prisma-statement.org).

Electronic Search

A combination of migration specific search terms (migration, migrant, emigrant, immigrant, expatriate, foreign worker, labor migration, left-behind, migrant families) and country specific search terms (Nepal, Nepalese, Nepali, UAE, United Arab Emirates, GCC, Gulf Cooperation Council, Middle East, Bahrain, Saudi Arabia, Oman, Qatar, Kuwait, Malaysia) were used to identify relevant studies using EMBASE, MEDLINE, Scopus and Global Health databases (Appendix 1). The search aimed to identify all relevant studies regardless of any health outcomes used. As such, no health outcome specific terms were used to limit the electronic search. Reference lists of the relevant studies including those of related systematic reviews and reference lists of the selected studies were further screened to identify potentially eligible studies.

Inclusion and Exclusion Criteria

Studies were eligible if they: 1) included Nepalese migrant workers aged 18 or older working in the GCC countries or Malaysia or returnee migrant workers from these countries; 2) provided primary data on health and wellbeing status/issues (physical health, mental health, accidents and injuries); and 3) were published in English language before 8 May 2020.

Article Screening and Selection

Once the electronic search was completed, the identified articles were exported to Rayyan (https://rayyan.qcri.org/welcome) and screening was carried out by two reviewers (SW and KK) independently to identify eligible articles. The titles of the identified studies were screened

For peer review only - http://bmjopen.bmj.com/site/about/guidelines.xhtml

to remove any duplicates and irrelevant articles. The abstract of all remaining articles was screened to identify eligible full text articles. Full text articles were reviewed and a consensus was reached to finalise the articles for inclusion. If more than one study were published using the same data source (e.g. routine healthcare date), we used the study with the largest sample size. Any disagreement over eligibility of studies was resolved through discussion with the third reviewer (PP).

Data Extraction and Synthesis

The information extracted from each article included: study reference (authors, publication year and country), study design and settings, participants' characteristics (sample size, age, and gender), health outcomes and key findings (Table 1). Extracted data were analysed and a summary of the narrative synthesis is reported in the results section. Meta-analysis was not conducted as there was heterogeneity in the outcome measured and the measurement tools used in the studies.

Quality Assessment

The PRISMA guideline suggests that systematic review should assess the risk of bias (based on theoretical grounds) rather than study quality (the best authors could do in the setting). However, we assessed the latter as the studies included in this review were predominately cross-sectional in nature with methodological limitations[8]. Quality assessment for this review was done using the Joanna Briggs Institute (JBI) Critical Appraisal Tools[9]. The JBI prevalence study critical appraisal tool was used for cross-sectional studies estimating the prevalence of the condition. The tool contains nine items covering domains related to sampling, outcome assessment, statistical analysis and response rate. Each item was scored one if the response was 'Yes' and scored zero if the response was 'NO' or 'Unclear'. As in the previous review[10], studies with eight or more 'Yes' response were rated as 'high' quality, four to seven as 'moderate' and three or below as 'low' quality. Similarly, the JBI analytical cross-sectional study critical appraisal tool was used for cross-sectional studies reporting effect sizes. The checklist contains eight items covering domains related to sampling, exposure, outcome, confounding factors, and statistical analysis (maximum possible score eight). Studies were categorised as high quality (seven or above), moderate quality (between five and six) or low quality (four and below). Qualitative studies were assessed by using the JBI qualitative study critical appraisal tool. The checklist contains ten items with domains covering methodological

For peer review only - http://bmjopen.bmj.com/site/about/guidelines.xhtml

approach, data collection, analysis and interpretation, researcher's role, participants' voice and ethics. The studies were rated high quality (eight and above), moderate quality (between five-seven) or low quality (four and below) as on the previous publication[11]. The assessment was undertaken independently by two reviewers (SW and KK) with any discrepancies resolved by a third reviewer (PP). As the number of studies in this population is limited, we did not exclude studies based on quality assessment. The results of the quality assessment are presented in Appendix 2.

Results

Screening Results

Database searches yielded 2770 articles. After duplicate removal, titles of the 2562 articles were screened and 2253 were excluded. Abstracts of the remaining 309 publications were further screened and 215 of these were excluded. Full text screening of the remaining 94 papers were carried out and a further 61 papers were excluded for various reasons (Figure 1). Altogether, 33 papers were included in this review; 31 were quantitative and two were qualitative studies.

For peer review only - http://bmjopen.bmj.com/site/about/guidelines.xhtml

Figure 1: PRISMA Flow Diagram of Study Selection

For peer review only

For peer review only - http://bmjopen.bmj.com/site/about/guidelines.xhtml

Study Characteristics

A total of 33 papers were included in the review among them 12 studies were conducted in Qatar [12-23], eight in Malaysia[24-31], nine in Nepal[5,7,32-38], two in Saudi Arabia[39,40], one each in UAE[41] and Kuwait [42]respectively. Two study included all GCC countries and Malaysia[7,38], another two study included Malaysia, Qatar and Saudi Arabia [32,33]and further two included in Qatar, Saudi Arabia and UAE[5,34] (Table 1). The study design varied across the studies; the review included 13 retrospective analysis of routine healthcare data [7,12-15,18,20,25,35,37,39,40,42] and 18 cross-sectional studies[5,16,17,19,21-24,26-34,41]. Only two studies were qualitative in nature[36,38]. Nine studies focused specifically on Nepalese migrants as their primary study population [5,7,32-38] whilst the remaining 24 studies mentioned Nepalese migrant workers as part of a sub-analysis (Table 1). In majority of the studies, there was a lack of disaggregated data on demographic characteristics of Nepalese migrant workers. There was a paucity of research with female migrant workers, with just one study identified in this review [7]. The study mainly fell into two categories: those exploring the health risks and experiences of migrants while abroad and those focusing on infectious diseases (mostly done as a part of arrival screening).

Studies Exploring Health Risk and Experiences

Occupational Health and Hazards

Seven studies (four high quality and three moderate quality) specifically assessed occupational morbidity, mortality, and fitness to work in the destination countries [15,20,25,32,35,36,39]. Majority of these studies were conducted in male migrant workers and the sample of Nepalese migrants varied from 20 to 38,908. Adhikari et al (2017) reported that around one-fifth (17%) of migrant workers had experienced work related accidents[32]. Poor working environment and not being registered with a doctor was associated with a greater perceived health risk at the work place. Another study reported that over a quarter (27.9%) of migrant workers had experienced occupational injuries: more than half (52%) of these workers fell from a height, 21% had injuries due to fall of a heavy object, 17% had motor vehicle accident injuries, 5% had machinery injuries and remaining 5% had other work related injuries[15]. In a study conducted in Saudi Arabia, Nepalese migrant workers were the third-most unfit population to work; 1.6% were unfit due to the presence of infectious disease and 5.3% due to non-communicable disease[39]. Another study reported that more than one quarter (25.4%) of migrants had traffic related pedestrian injuries during abroad work[20] (Table 1). A study by Pradhan et al conducted a retrospective analysis of Government of Nepal data from 2009–2017and recorded 1345

For peer review only

deaths, of which workplace accident and road traffic accidents contributed to 12% and 10% deaths respectively[35]. 33 cases of work-related ocular injuries were reported in one study among Nepalese patients of the 440 patients attending a hospital in Malaysia[25]. One qualitative study explored workplace accidents in GCC and Malaysia and reported several issues faced by the workers including lack of workplace safety, long working hours resulting in dehydration and heat stroke and injuries and accidents related issues including life-long disability[36].

Sexual Health

Only one moderate quality study in this review assessed the knowledge, attitudes and perceptions (KAP) of HIV/AIDS related risks[34]. The study was conducted among 408 adult Nepalese migrants (92% male) with at least six months of work experience in one of the three Gulf countries (Qatar, Saudi Arabia and UAE). The study showed that 91% of respondents had concerns about HIV/AIDS, and 17.2% of workers reported having sexual intercourse with a partner other than their spouse within the last 12 months. More than half (59%) of the respondents perceived themselves at high risk of being infected due to their sexual activities[34] (Table 1).

Mental Health

Five studies (all moderate quality) examined mental health issues among migrant workers. The sample of Nepalese migrants workers in these studies ranged between 20 to 1354[7 21 33 35 38]. One study on Nepalese female returnee migrant workers from Middle East and Malaysia reported the prevalence of mental health problems as 8.3%[7]. Another study reported that almost a quarter (23%) of labour migrants to Malaysia, Qatar and Saudi Arabia had experienced mental health issues, with a strong positive link between perceived health risk in the work environment and mental health status[33]. Third study reported a paradoxical finding with 4% increase in the predicted probability of depressive symptoms among Nepalese migrant workers compared to Arab, for every unit increase in perceived quality of life[21]. One study analysed Nepalese government's report and looked at 1354 deaths in Nepalese migrant workers, of which 8.5% were due to suicide[35]. The fifth quality qualitative study reported various mental health problems among the workers including loneliness, social isolation, tensions, anxiety, attempt to suicide[38] (Table 1).

For peer review only - http://bmjopen.bmj.com/site/about/guidelines.xhtml

Healthcare Access

Five studies (one high and four moderate quality) focused on labour migrants' healthcare access issues and the number of Nepalese workers in these studies ranged between 20 to 942 respectively [5 7 32 37 38]. Adhikari et al (2017) reported that workers who were not registered with a doctor had poor health outcomes compared to those who were registered[32]. Another study also reported that only 36.5% workers had access to health insurance and about half (48.7%) did not have paid sick leave during their health problems[5]. Another study on Nepalese female returnee migrant workers reported that only 11% of respondents received health services during their abroad work[7]. The fourth study reported that only insurance 68% of the workers had health insurance abroad and only 20% underwent regular health check-up[37]. In the qualitative study, participants reported poor access to mental health services related, mainly related to communication problems, and stigma to mental health[38] (Table 1).

Other Health Issues

A total of five studies (two high and three moderate quality) involving participants number ranging between 44 to 1354 reported various health issues [7 19 22 35 37]. One study on Nepalese female returnee migrant workers reported a prevalence of workplace abuse, torture or maltreatment at the workplace, and physical harm at 41%, 31% and 11%, respectively[7]. Clinical prevalence of oral lesions among migrant workers was found to be 4.6%[19]. Third study looked at the chronic kidney disease among workers and found that 13.6% of workers had diabetic nephropathy[37]. In the study by Pradhan et al, cardiovascular disease, natural/others reasons and murder contributed to 42%, 25% and 1.7% of deaths respectively[35]. The last study reported that of patients attending to the emergency medical service in Qatar, out-of-hospital cardiac arrest among Nepalese migrant patients was found to be 11.6%[22] (Table 1).

Studies on Infectious Diseases (Parasitic and Bacterial Infections, TB and Hepatitis E)

Of the 33 included studies, 17 studies (nine high and eight moderate quality) reported the proportion of sero-and/or faeco positive cases of infectious diseases (parasitic and bacterial Gastroenteric infections, tuberculosis, hepatitis E) [12-14 16 17 19 23 24 26-31 40-42]. The number of Nepalese workers included in these studies ranged between 12 to 1429. In several of these studies, Nepalese migrant workers had the higher proportion of infectious disease cases among the population studied. These infectious diseases included, toxoplasmosis (46.2%, working in Malaysia)[24], tuberculosis (7%, Saudi Arabia and 11%, Qatar)[14 40], tuberculosis meningitis

For peer review only - http://bmjopen.bmj.com/site/about/guidelines.xhtml

(37.5%, Qatar) [18], diarrhoeal bacterial infection (26.6%, Qatar)[16], protozoan ova/cysts (13.7%), helminths (6.2%), and hookworms (4.3%, Qatar)[12], Hepatitis E (74%, Qatar)[17], Brugian Lymphatic Filariasis (BmR1) (2.9%, Malaysia) and parasitic infection (BmSXP) (13%, Malaysia)[26]. Moreover, prevalence of salmonella among Nepalese migrant food handlers (3.7% Malaysia)[28], mean knowledge of food cleanliness and hygiene (73.1%, Malaysia) and symptom of foodborne illness (18.4% Malaysia)[30] (Table 1).

Overall Quality Assessment

More than half of the cross-sectional prevalence studies (54% n=15/28) scored as 'high' quality and remaining were of moderate quality[7 12 15-17 19 26 28 30 35 37 40 41]. Similarly, three analytical studies were rated as moderate quality [21 32 33] and the two qualitative studies were rated as one high and one of moderate quality[36 38]. None of the studies were rated as poor quality. The results of the quality assessment scores are presented in Table 1 and details is presented in Appendix 2.

For peer review only

For peer review only - http://bmjopen.bmj.com/site/about/guidelines.xhtml

Table 1: Characteristics of studies included (n=33)

Author, Year, Country	Study Design and Setting	Participant Characteristics	Health Outcomes (Measurement Tools)	Key Findings	QA Scores
**Health risk and experiences related issues**					
**Dhakal et al, 2020, Nepal**	Hospital record data evaluated from the hospital data in Nepal (January – July, 2019)	Returnees migrant participants – 44<br>Gender – Male – 95% (n=42)<br>Age – Mean age 37.2 years	Healthcare access and prevalence of Chronic Kidney Disease (CKD)	- Workers with health insurance 68.2% (95% CI 52.4-81.3) (n=30)<br>- Underwent for routine health check-ups annually 20.4% (95% CI 9.8-35.3) (n=9)<br>- No regular health check-up 79.5% (95% CI 64.7-90.0) (n=35)<br>- Exposed to chemicals 27.3% (95% CI 14.9-42.7) (n=12)<br>- Patients were unknown about cause of CKD 77.3% (95% CI 62.1-88.5) (n=34)<br>- Had diabetic nephropathy .13.6% (95% CI 5.1-27.3) (n=6)<br>- Death due to kidney failure (n=1)	Moderate
**Khaled and Gray, 2019, Qatar**	Cross-sectional survey, February 2016	Migrant workers in Qatar<br>Total participants – 2520<br>Nepalese – 26% (n=655)<br>Gender-NR<br>Age - NR	Depressive symptoms	- Compared to Arabs, Nepalese migrant experienced 4%, increase in the predicted probability of depressive symptoms, for every unit increase in perceived quality of life.	Moderate
**Regmi et al, 2019, Nepal**	Qualitative Study (data collected in 2017)	Returnee migrants in Nepal from Qatar, Saudi Arab, Malaysia, Oman, UAE<br>Sampled - 20	Various health issues	- Unfair treatment and discrimination at work<br>- Poor working and living arrangements – dirty toilets and bathrooms<br>- Lack of security, loneliness and poor social life at work place/social isolation<br>- Mental health problems – tensions, anxiety and attempt to suicide and poor access to mental health services<br>- Poor communication facilities<br>- Only formality of pre-departure training package – contents good but poor implementation	Moderate
**Adhikary et al. 2019, Nepal**	Qualitative study (July to September, 2011)	Returnee migrants, interviews conducted interviews in Nepal – 20<br>Male – All<br>Mean age – 31.3 years	Workplace accidents among Nepali male workers in Qatar, Saudi Arabia and Malaysia.	Work place related issues:<br>- Not safe workplace<br>- High work pressure<br>- No medical supports from employer in host country<br>- Long working hours, mostly without timely food and drinking water resulting in dehydration and heat stroke<br>- Communication difficulty due to language barriers	High

For peer review only - http://bmjopen.bmj.com/site/about/guidelines.xhtml

Author, Year, Country	Study Design and Setting	Participant Characteristics	Health Outcomes (Measurement Tools)	Key Findings	QA Scores
				- Injuries and accidents related issues<br>- Fall from the roof, trapped in the hole<br>- Injured back bone, legs, hands and head<br>- Life-long disability	
**Pradhan et al, 2019, Nepal**	Retrospective analysis of Government of Nepal provided data (2009 – 2017)	Nepali migrant workers in Qatar<br>Total sample – 1354<br>Gender –NR<br>Age – NR	Analysed the deaths of Nepalese migrant workers	Causes of death due to:<br>- Cardiovascular –42% (95% CI 39.5-42.8) (n=571)<br>- Suicide – 8.5% (95% CI 7.1-10.1) (n=116)<br>- Workplace accident – 12.4% (95% CI 10.7-14.3) (n=169)<br>- Road traffic accident – 10.1% (95% CI 8.5-11.8) (n=137)<br>- Murder – 1.7% (95% CI 1.0-2.5) (n=23)<br>- Natural/others reasons for death – 25% (95% CI 22.6-27.3) (n=338)	Moderate
**Adhikary et al, 2018, Nepal**	Cross-sectional questionnaire-based survey	Male Nepalese construction workers, worked in host countries (Malaysia, Qatar and Saudi Arabia) for >6 months.<br>Total participants – 403<br>Age - NR	Self-reported health and wellbeing status	13.2% (95% CI 10.0-16.8) (n=53) reported poor/very poor health, relating to:<br>- Age older than 40 year reported as poor health (OR= 3.0, 95% 1.0-9.0)<br>- Poor work environment (OR= 6.8, 95% CI 3.2 – 14.6)<br>- Health risks at work (OR= 4.7, 95% CI 2.1-10.5)<br>- Prevalence of mental health issues was 23% overall - strong link between perceived health risks and mental health status.	Moderate
**Adhikary et al; 2017, Nepal**	Cross-sectional questionnaire-based survey	Male Nepalese construction and factory workers, worked >6 months in Malaysia, Qatar or Saudi Arabia.<br>Total participants – 423<br>Age -NR	Self-reported perceived health risks and accidents at work	Poor or very poor work environment (rated by the workers) associated with greater perceived health risk at work (OR 2.5, 95% CI=1.5-4.4)<br><br>Prevalence of accidents at work=17%<br>- Variables associated with accidents at work included: >Age 40 and above vs 20-29 (OR= 4.0, 95% CI=1.7-9.7)<br>- Not Satisfied accommodation vs satisfied with accommodation (OR=1.9, 95% CI=1.1-3.4)<br>- Poor or very poor work environment vs good/good to fair environment (OR 3.5, 95% CI=1.8-6.7)<br>- Working in Middle-East vs Malaysia. (OR .3.6, 95% CI=1.5-8.5)<br>Not registered with a doctor vs registered (OR=0.3, 95% CI=0.1-0.7)	Moderate
**Simkhada et al, 2017, Nepal**<br><br>***[Data for GCCs and Malaysia***	Retrospective analysis of NGO collected data (July 2009 to July 2014)	Returnee Nepalese female migrant workers from GCC and Malaysia<br>Total particpants-942<br>GCC=933	Various health issues while working in GCC, middle-east and Malaysia	Proportion female with health problems - 24% (95% CI 21.3-26.8) (n=226)<br>- Abuse at workplace -37% (95% CI 33.6-39.9) (n=346)<br>- Accident at workplace - 1.1% (95% CI 0.5-1.2) (n=10)<br>- Mental health problem - 8.3% (95% CI 6.6-10) (n=78)	Moderate

For peer review only - http://bmjopen.bmj.com/site/about/guidelines.xhtml

Author, Year, Country	Study Design and Setting	Participant Characteristics	Health Outcomes (Measurement Tools)	Key Findings	QA Scores
provided by the authors)	via Paurakhi Nepal (NGO)	Malaysia=9<br>Median age 31 (IQR 37)<br>Age range – 14-51 years	(prevalence calculated using information available from client Information Form/Sheet)	- Torture or maltreatment at the workplace 30.9% (95% CI 27.9-33.9-) (n=291)<br>- Pregnancy at work place - 3.1% (95% CI 2.1-4.3) (n=29)<br>- Sexual abuse – 51.7% (95% CI 32.5-70.5) (n =15/29)<br>- Physical harm -10.9% (95% CI 9.0-13.1) (n=103)<br><br>Received health services – 10.8% (95% CI 8.9-12.9) (n=102)	
**Irfan et al, 2016, Qatar**	Cross-Sectional study (June 2012-May 2013)	Patients attending to the emergency medical service in Qatar<br>Total participant – 447<br>Nepalese – 11.6% (n=52)<br>Gender-NR<br>Age – Median age 51 years (range 39-66 years)	Proportion of out of hospital cardiac arrest	Out-of-hospital cardiac arrest among Nepalese migrant patients – 11.6% (95% CI 8.8-14.9) (n=52). No further data.	High
**Min et al, 2016, Malaysia**	Retrospective cross-section of routine healthcare data (January 2011 to December 2013)	Patients attending to the eye casualty with work-related ocular injuries, in Hospital Sultan Ismail in Johor Bahru, Malaysia<br>Total 440 work-related ocular traumas.<br>Nepalese – 21.7% (n=33)<br>Gender –NR<br>Age - NR	Work related ocular traumas	33 cases of Nepalese work-related eye injuries. Causes range from open globe injuries due to being hit by a machine, nail, wood and metal whilst grinding.	High
**Al-Thani et al, 2015, Qatar**	Retrospective analysis of hospital trauma registry records 2010-2013 Hamad Trauma Centre	Total migrant participants - 2015<br>Nepalese –28% (n=563)<br>Male – 98% (n=1972)<br>Female –2% (n=43)<br>Age – NR	Proportion of occupational injuries and mortality cases	Overall proportion of occupational injury cases – 27.9% (n= 563), of which<br>- Falls from height – 52.4% (95% CI 48.1-56.5) (n=295)<br>- Fall of a heavy object – 20.4% (95% CI 17.1-24) (n= 115)<br>- Motor vehicle crashes injuries - 17% (95% CI 14.2-20.6) (n=97)<br>- Machinery injuries - 5% (95% CI 3.1-6.9) (n= 27)<br>- Others – 5% (95% CI 3.4-7.3) (n=29)	Moderate
**Latifi et al, 2015, Qatar**	Retrospective analysis of routine healthcare data	Total traffic related pedestrian injuries (TRPI) patients – 601<br>Total Nepalese expat TRPI patients – (n=147)<br>Gender –NR<br>Age – NR	Pedestrian morbidity and mortality	- 25.4% (95% CI 21.0-18.0) of TRPI were of Nepalese migrant workers (vs 16.0% of the general population of Qatar being Nepalese).<br>- 51.4% of TRPI with positive blood alcohol were Nepalese migrant workers.	High

For peer review only - http://bmjopen.bmj.com/site/about/guidelines.xhtml

Author, Year, Country	Study Design and Setting	Participant Characteristics	Health Outcomes (Measurement Tools)	Key Findings	QA Scores
**Joshi et al, 2014, Nepal**	Cross-sectional study	Nepalese migrants with experience of >6 months in Qatar, Saudi Arabia or United Arab Emirates.<br>Total participants – 408<br>Males – 92.4% (n=377)<br>Aged between 26-35 – 53.4% (n=218)	Knowledge of HIV/AIDS and risk perceptions	Risk perceptions of HIV/AIDS:<br>- Concerned about HIV/AIDS – 90% (95% CI 86.3-92.4) (n=366)<br>- Perceived themselves at high risk of being infected due to their sexual activities - 59.2% (n=397)<br>Sexual behaviour:<br>- 17.2% (95% CI 13.6-21.1) (n=70) had sexual intercourse with a partner other than their spouse during the last 12 months of their stay abroad.	Moderate
**Kavarodi et al, 2014, Qatar**	Population-based cross-sectional study	Low income expatriate workers from Indian sub-continent (living in Qatar for >6 months)<br>Total participants – 3,946<br>Nepalese – 5.4% (n=213)<br>Gender –NR<br>Age - NR	Clinical prevalence of suspected oral lesions	- Oral Lesions in of Nepalese workers 4.7% (95% CI 2.1-7.8) (n=10).	High
**Alswaidi et al, 2013, Saudi Arabia**	Review of Ministry and Health data from Saudi expat worker fitness screening programme (1997–2010)	Total number of registered expatriate workers - 4 272 480<br>Nepalese – 0.9% (n=38 908).<br>Females – 14% (n=5 367)<br>Males – 86% (n=33 541)<br>Age – NR	Proportion of 'unfit' to workers.	Cases of unfitness among Nepalese workers by gender:<br>- Unfit males – 1.99% (95% CI 1.8-2.1) (n=669)<br>- Unfit females – 1.2% (95% CI 0.9-1.5) (n=64)<br>- Overall unfit – 1.9% (95% CI 1.7-2.0) (n=733)<br><br>Nepalese migrants were the third most unfit population.<br><br>Nepalese migrants as proportion of all those with:<br>- Infectious causes of unfitness (incl. hepatitis, HIV, TB) – 1.6% (n=379)<br>- Non-communicable causes of unfitness – 5.3% (n= 354)	High
**Joshi et al, 2011, Nepal**	Cross-sectional questionnaire survey, Kathmandu (International Airport and nearby hotels/lodges).	Returnee Nepalese male and female migrant workers from Qatar, Saudi Arabia and UAE (n=408)<br><br>Male = 377 (92.4%)<br>Female = 31 (7.6%)<br>Mean Age (SD) – 32 (6.5) years<br>Age ranges – 18-53 years	Prevalence of health problems using self-reported/ questionnaire survey	Prevalence of health problem(s) – 56.6% (95% CI 51.6-61.4) (n=231)<br>- Most common problems:<br>- Headache or fever - 30.7% (95% CI 24.8-37.1) (n=71)<br>- Respiratory symptoms - 21.2% (95% CI 16.1-27.0) (n=49)<br>- Musculoskeletal problems – 19.9% (95% CI 14.9-25.6) (n=46)<br>- Gastrointestinal illness – 19.5% (n=45)<br>- Injuries/poisoning – 13.9% (95% CI 9.6-18.9) (n=32)<br>Prevalence of some type of injury or accident at their workplace - 25% (95% CI 20.8-29.5) (n=102)	High

For peer review only - http://bmjopen.bmj.com/site/about/guidelines.xhtml

Author, Year, Country	Study Design and Setting	Participant Characteristics	Health Outcomes (Measurement Tools)	Key Findings	QA Scores
				- Health insurance in host countries- 36.5% (95% CI 31.8-41.4) (n=149)<br>- Sought health services or treatment in the working countries- 83.1% (95 % CI 42.1-51.0) (n=192)<br>- Lack of provision of leave during health problem(s)- 48.7% (n=19)	
**Infectious diseases related issues**					
**Al-Awadhi et al, 2019, Kuwait**	Retrospective analysis of routine healthcare data (2015 to 2017)	Migrant workers in Kuwait<br>Total examined participants – 1000<br>Nepalese – 3.3% (n=33)<br>Age - NR<br>Gender-NR	Prevalence of T solium by screening blood using a sensitive taeniasis-specific anti-rES33 antibody assay.	- 6.1% (95% CI 0.7-20.0) (n=2) of Nepalese migrant worker sample tested for T Solium taeniasis-specific lgG antibodies	High
**Sahimin et al, 2019 Malaysia**	Cross-sectional study (September 2014 – August 2015)	Migrant workers from manufacturing, services, agriculture and plantation, construction and domestic work sectors in Malaysia<br>Total participants - 610<br>Nepalese -(n=103)<br>Gender – NR<br>Age - NR	Measure prevalence of *E. dispar and E. histolytica*	- *E. dispar* 4.9% (95% CI 1.4-12.2)and *E. histolytica* infections 3.7% 95% CI (0.8-10.4)	High
**Sahimin et al, 2018, Malaysia**	Cross-sectional study	Migrant workers in Malaysia.<br>Total stool samples examined – 388<br>Nepalese –20.9% (81)<br>Gender –NR<br>Age - NR Gender –NR<br>Age - NR	Prevalence of Giardia duodenalis and Cryptosporidium parvum	- Giardia duodenalis 1.8% [0.7−3.7] and Cryptosporidium parvum 0.3 [0.0−1.4] respectively	High
**Dafalla et al, 2017, UAE**	Cross-sectional survey conducted at public health clinic	Immigrant workers – food handlers, babysitters, housemaids, drivers working in Sarjaha, UAE<br>Total participants– 21,347 (number of Nepalese workers not reported)<br>Total infected population –3.3% (n=708)<br>Gender –NR<br>Age – NR	Prevalence of parasitic infections (Examined microscopically and screened for intestinal parasites)	Proportion of infected migrant workers that are Nepalese – 6.2% (95% CI 4.5 – 8.2) (n=44)<br>- All protozoal infections: 7% (95% CI 5.9 – 8.6) (n=33)<br>- All helminth infections: 4.2% (95% CI 9.8 – 35.3) (n=9)	Moderate

For peer review only - http://bmjopen.bmj.com/site/about/guidelines.xhtml

Author, Year, Country	Study Design and Setting	Participant Characteristics	Health Outcomes (Measurement Tools)	Key Findings	QA Scores
**Noordin et al, 2017, Malaysia**	Cross-sectional survey (September 2014 to August 2015)	484 migrant workers from manufacturing, services, agriculture and plantation, construction and domestic work sectors.<br>Nepalese – 21.3% (n=103)<br>Gender –NR<br>Age – NR	Prevalence of parasitic infections	- Sero-prevalence of brugian Lymphatic Filariasis [BmR1] – 2.9% (95% CI 0.6 – 8.2) (n=3)<br>- Prevalence of parasitic infections (BmSXP) –12.6% (95% CI 6.8 – 20.6) (n=13)	Moderate
**Sahimin et al, 2017, Malaysia**	Correctional survey (Sept. 2014- Aug. 2015)	484 migrant workers<br>Nepalese respondents- 20.5% (n = 99)<br>Conducted at five working sectors (manufacturing, construction, plantation, domestic and food services)	Sero-prevalence T. gondii through Questionnaire survey and laboratory blood tests	Sero-prevalence:<br>- IgG – 74.7% (95% CI 65.0 – 82.9)<br>- lgM – 6.1% (95% CI 2.3 – 12.7)	High
**Woh et al, 2017, Malaysia**	Cross-sectional study	Healthy, asymptomatic migrant food handlers.<br>Total participants – 317<br>Nepalese – 25.2% (n=80)<br>Gender –NR<br>Age – NR	Prevalence of Salmonella carriers, using stool samples	- Prevalence of salmonella amongst Nepalese migrant food handlers – 3.7% (95% CI 0.7 – 10.5) (n=3)	Moderate
**Abu-Madi et al, 2016a, Qatar**	Retrospective analysis of routine healthcare data (2005 to 2014)	Records held at Hamad Medical Corporation data-base for subjects referred for stool examination<br>Total participants - 29,286<br>Nepalese – 4.8% (n=1429)<br>Gender –NR<br>Age - NR	Proportion of helminth infections positive cases.	- Highest proportion of helminth infections among Nepalese workers – 15.3 % (95% CI 13.39–17.12)	High
**Abu-Madi et al, 2016b, Qatar**	Retrospective analysis of routine healthcare data	Recently arrived migrant workers in Qatar<br>Total participants – 2,486<br>Nepalese – 15% (n=373)<br>Gender –NR<br>Age - NR	Presence of intestinal parasites (helminths and protozoa)	Proportion of positive cases in Nepalese migrant workers:<br>- Helminths combined - 6.2% (95% CI 3.8–9.6)<br>- Hookworms - 4.3% (95% CI 2.4–7.3)<br>Protozoa combined - 13.7% (95% CI 10.0–18.2)	Moderate
**Humphery et al, 2016, Qatar**	Community-based survey, Doha	Total participants– 126<br>Nepalese – 29.3% (n=37)<br>All male population<br>Median age (IQR) in years = 33 (27–39)	Prevalence of gastrointestinal pathogens (detected using polymerase chain reaction)	- Total prevalence of gastrointestinal pathogens = 62.7% (95% CI 53.6 – 71.1) (n=79)<br>- Gastrointestinal pathogens amongst Nepalese migrant workers – 26.6% (95% CI 10.6 – 24.3) (n=21)	Moderate

For peer review only - http://bmjopen.bmj.com/site/about/guidelines.xhtml

Author, Year, Country	Study Design and Setting	Participant Characteristics	Health Outcomes (Measurement Tools)	Key Findings	QA Scores
**Woh et al, 2016, Malaysia**	Cross-sectional survey (October 2014 to May 2015)	Migrant food handlers living in Malaysia<br>Total participants – 383<br>Nepalese – 24.8% (n-95)<br>Gender – NR<br>Age - NR	Knowledge and practices regarding the food handlings	Mean knowledge scores on:<br>- Symptom of foodborne illness among Nepalese migrant – M = 18.4%, SD = 28.8<br>- food cleanliness and hygiene – M= 73.1%, SD = 15.3<br>Proportion of food handling practices among Nepalese migrant –<br>- Poor practices - 21.9% (n=7)<br>- Moderate – 14.3% (n=32)<br>- Good – 43.8% (n=56)	Moderate
**Imam et al, 2015, Qatar**	Retrospective analysis of routine healthcare data (January 2006 and December 2012)	Patients with suspected or confirmed tuberculous meningitis.<br>Total participants – 80<br>Nepalese – 37% (n=30)<br>Gender –NR<br>Age - NR	Clinical presentation, diagnosis, treatment, outcome, and the incidence of adult tuberculous meningitis	- 30/80 patients with tuberculous meningitis were Nepalese (37.5% (95% CI 26.9-49.0). No further data.	High
**Chattu and Mohammad, 2013, Saudi Arabia**	Retrospective analysis of routine healthcare data from Qassim region (January 2005 to December 2009)	Migrant workers (n= 165)<br>Male – 42% (n=70)<br>Female –58% (n=95)<br>Age - NR	Proportion of reported TB cases, using laboratory test	- Proportion of migrant workers with tuberculosis from Nepal: 7% (95% CI 3.8-12.3) (n=12).	Moderate
**Abu-Madi et al, 2011, Qatar**	Cross-sectional survey (June – September, 2009)	Patients resident in Qatar who were randomly recruited and conducted survey – 1538<br>Nepalese – 15.3% (n=236)<br>Gender – Male – 98.3% (n=232)<br>Female – 1.7% (n=4)<br>Age – mean age 28.2 years	Prevalence of intestinal parasitic infections among food handlers and housemaids)	Prevalence of all types of parasitic infections (species) – 29.7%, (95% CI 25.51 - 34.15)<br>- Helminths – 23.7%, (95% CI 19.91-27.98)<br>- Hookworms – 17.8%, (95% CI 14.40-21.73)<br>- A. lumbricoides – 2.5%, (95 CI (1.40-4.50)<br><br>Prevalence of all Protozoa – 9.7%, (95% CI 7.23-12.93)<br>- B. hominis – 3%, (95% CI 1.69-5.01)<br>- Prevalence of non-pathogenic:<br>- Amoebae – 3%, (95% CI (.69-5.01)<br>- G. duodenalis – 3.4%, (95% CI 2.02-5.52)	High
**Ibrahim et al, 2009, Qatar**	Community based survey, Alkhor hospital.	anti-HEV IgG Nepalese migrants nationally – 86<br>58 of these seen at Alkhor Hospital.<br>Gender –NR	Prevalence of Hepatitis E (using ELISA test) and other clinical symptoms	Prevalence of acute HEV amongst those seen at Alkhor Hospital – 74% (95% CI 60.9-84.7) (n=43)<br>- admitted to hospital – 95.3% (95% CI 84.1-99.4) (n=41)	Moderate

For peer review only - http://bmjopen.bmj.com/site/about/guidelines.xhtml

Author, Year, Country	Study Design and Setting	Participant Characteristics	Health Outcomes (Measurement Tools)	Key Findings	QA Scores
		Aged 26.7 (SD-5.6, range 19–41 years)			
**Chan et al, 2008, Malaysia**	Cross-sectional survey conducted in a plantation and detention camp of Malaysia	Total foreign migrant workers - 501<br>Nepalese – 5% (n=26)<br>Gender –NR<br>Age - NR	*Toxoplasma gondii* IgG and IgM sero-prevalence	- Prevalence of Toxoplasma gondii IgG – 46.2% (95% CI 26.5-66.6) (n=12)<br>- Prevalence of Toxoplasma gondii IgM – 11.5% (95% CI 2.4-30.0) (n=3)	High
**Al-Marri, 2001, Qatar**	Population-based retrospective analysis (January 1996 to December 1998)	Total cases of positive *M. tuberculosis* culture and sensitivity – 406<br>Nepalese migrant cases – 11% (n=44)<br>Gender –NR<br>Age - NR	Drug resistant cases of TB (where positive isolates identified)	- Of total 386 cases of pulmonary TB (321 expats) identified, 11% (95% CI 7.9-14.2) n= 44, Nepalese cases of TB, of which 9 cases were drug resistant.	High

For peer review only

Discussion

To our knowledge, this is the most comprehensive review of the health and well-being status/issues of the Nepalese migrant workers in the GCC countries and Malaysia. The resultant lack of disaggregated demographic data means that the overall characteristics of Nepalese participants is difficult to determine. The dissonance between issues covered in the peer-reviewed and grey literature for this population, namely in national and international media and in government reports, is notable. Disproportionately few studies focused on occupational mental, and sexual health of migrant workers.

Occupational Health

Our review identified seven papers focusing on occupational morbidity, mortality and fitness to work in the destination countries[15 20 32 35 36 39]. Only three of these focused solely on Nepalese migrants, and none compared occupation or working conditions with morbidity and mortality experienced[32 36 35]. This a crucial gap in the literature and further studies are needed to guide policy change. There has been widespread media coverage of the poor working conditions faced by Nepalese migrant workers and health impacts of these conditions are highlighted by the plight of manual labourers working for the forthcoming 2022 FIFA Qatar World Cup. Close to a fifth of labour migrants to Malaysia, Qatar and Saudi Arabia had experienced a workplace accident[32]. According to a Nepalese government report, there were circa 7,467 deaths among Nepalese migrant workers abroad between 2008/09 and 2018/19, and over 40% of the deaths were deemed either of natural or other/unidentified cause[3]. Despite these workers being young (mean age 29 years) and fit (assessed by health screening both at home and destination countries), the magnitude of the proportion of these deaths is unusual in these groups[3]. This raises questions about robustness of post-mortem investigative practices and classification methodologies, a concern highlighted by both the Nepalese government and civil society groups[43]. Indeed, Pradhan et al suggest that many deaths attributed to cardiovascular diseases and 'natural causes' correlate with longer hours worked in high temperatures in this setting[35]. It is worth noting that Nepalese migrant workers themselves are not oblivious to these occupational risks- those who reported a poor or very poor work environment were found to be 3.5 times more likely to suffer a workplace accident[32].

For peer review only - http://bmjopen.bmj.com/site/about/guidelines.xhtml

Mental Health

Five studies in the review reported on mental health issues. Adhikari et al (2018) reported that almost a quarter of labour migrants to Malaysia, Qatar and Saudi Arabia had experienced mental health issues, with a strong positive correlation between perceived health risk in the work environment and mental health status[33]. The qualitative study by Regmi et al (2019) highlighted various mental health problems among the workers including loneliness, anxiety, and attempt to suicide[38]. Similar findings were reported in a cross-sectional study of 5000 migrant workers in Shanghai, where 21% reported mental disorders such as obsessive-compulsive disorder, anxiety, and hostility[44].

The Nepalese government report suggests that suicide is a significant cause of mortality in labour migrants to GCC countries and Malaysia, and there is evidence that mental health is an underexplored issue facing this population[45-47]. Only one of the study in this review looked at the suicide cases with nearly 10% of the deaths in these workers resulting from suicide[35]. The paucity of peer-reviewed studies exploring risk factors of poor mental status and psychiatric morbidity for this population requires urgent attention.

Migration for work is a time of significant turmoil: new language, new culture and poor working conditions. Loss of protective familial and wider social networks exacerbate feelings of homesickness, loneliness and hopelessness that commonly develop amongst this population[48-50]. Psychiatric under-diagnosis is common in deprived populations and is compounded by poor screening of those with pre-existing psychiatric conditions[51-54]. The result is lack of mental health support and omission of medications in destination contexts that can worsen conditions. Most common psychiatric morbidity in this population centred around depressive and anxiety-related disorders, although the impact of addiction particularly of alcohol consumption remains underexplored[47 55-57]. The impacts of labour migration on the mental health of left-behind families is also important, but beyond the scope of this review[45 58].

Sexual Health

Only a single study in this review examined sexual health issues amongst this population and exploring HIV/AIDS knowledge, attitudes and perceptions amongst Nepalese migrant workers. Joshi et al; (2014) reported that over 17% had had sexual intercourse with someone other than their spouse or partner during the final 12 months of their stay abroad[34]. This highlights higher levels of sexual risk taking behaviour, echoed by studies focusing on Nepalese migrants to India, which showed widespread use of local female sex-workers by male

For peer review only - http://bmjopen.bmj.com/site/about/guidelines.xhtml

Nepalese migrant populations, multiple sexual partners and low levels of condom use. Whilst there may be differences between the Indian and GCC or Malaysian contexts, the authors note there is a clear dearth of evidence around non-HIV/AIDS related sexual health of these migrants, and the impact of this on left-behind families[59 60]. Similar findings also revealed from the studies in Bangladesh and China among migrant workers at high risk of heterosexual HIV acquisition[61 62].

Infectious Disease

Out of 33 studies, 17 studies focused on migrant workers in a destination country and provided minimal disaggregated analysis on the Nepalese sub-population. Majority of these were done as a part of arrival screening and focus on infectious diseases were conducted from a destination country perspective. Overwhelmingly, the discussion sections of these studies focused on Nepalese migrant workers as potential vectors for transmitting infectious diseases to native population. This health security framing overlooks Nepalese labour migrants as a vulnerable population by virtue of their poor socioeconomic status in their origin country as well poor working and living conditions, and poor access to healthcare in destination countries[5 63 64]. Similar findings were also reported in a study from Singapore where a relatively high prevalence of malaria, hepatitis and tuberculosis was reported among migrant workers in Singapore[65]. Migrant workers in South Asia generally appear to have a greater prevalence of infectious diseases due to the complex interaction of several factors- this includes higher prevalence of infectious diseases in their native countries together with aforementioned poor access to healthcare and low socioeconomic status[6]. Acknowledgment and consequent introduction of policies to improve these structural drivers of infectious diseases amongst Nepalese migrants would be a more holistic approach that might both better protect the local population and improve the health and wellbeing of the vulnerable migrant population[66.]

Literature Gap for Female Migrant Workers

Women comprise only 7% of Nepalese labour migrant abroad[5]. However, the role of women in the migration story is far more significant and complex than this figure betrays with regards to true numbers of women migrating, roles of women 'left behind' and how it has influenced gender norms in Nepalese society. The complex interplay between various factors such as socio-cultural norms, women's role in decision-making, and freedom to mobility reflect on their health from access to sexual and reproductive health services to gender-based violence[67]. Just one study has previously attempted to capture health outcomes among female migrants[7].

For peer review only - http://bmjopen.bmj.com/site/about/guidelines.xhtml

They highlighted that almost a quarter of female Nepalese migrants faced multiple health problems and over 40% had faced workplace abuse, with close to half of the 3% that reported becoming pregnant whilst away doing so as a result of sexual abuse[7].

Female labour migration from Nepal has increased significantly over the past decade, driven by increasing demands in primarily GCC destination countries, poor agricultural employment opportunities and a slowly-changing gender norms[68]. One third of remittances to Nepal are from female migrant workers[7 69,]. Higher proportion (90%) of female labour migrants are undocumented workers in Gulf countries and this may have resulted from the restrictive governmental labour migration policies such as prohibition of women to work in the Gulf domestic sector[70]. Precarious channels of migration bring greater risks of exploitation and harm to health[71], yet neither the peer-reviewed literature in health, nor do wider literatures reflect the magnitude of these issues. More work is required on the health of Nepalese female migrants abroad, as well the challenges in reintegration that they face on their return[68].

Strengths and Limitations

This review has several strengths. As mentioned earlier, the review is the most comprehensive review to date on this population. As GCC and Malaysia are the most attractive destinations for migration, the findings of this review will have important research implications in terms of highlighting the research gap on specific health problems of migrant workers in general as well as the lack of research focus on female migrant workers. This review also has important practical implications, such as informing the design of culturally appropriate care and outreach for Nepalese workers. Secondly, not restricting studies based on particular health outcomes, peer reviewed studies looking at a range of health issues in this population were included. Screening of studies and quality assessment was conducted by two independent reviewers, ensuring low risk of selection bias in this review. We applied research design specific quality assessment tools, providing the accurate ratings of the articles. However, there were a number of limitations. The review did not systematically include grey literature although a number of key reports were used as reference points to compare to our findings from the peer-reviewed literature. The risk of missed studies by only searching English language databases is noted, particularly through exclusion of relevant Nepalese peer-reviewed journals. Also, recent guidelines have been published on reporting of narrative synthesis without meta-analysis[72], however these guidelines are more applicable for intervention studies, thus we have not used these in this narrative systematic review. As the number of qualitative studies were very small

For peer review only

For peer review only - http://bmjopen.bmj.com/site/about/guidelines.xhtml

(n=2), we reported the key findings from these studies rather than conducting a separate meta-synthesis.

Conclusion

This review identified a number of health issues among Nepalese migrant workers in the GCC countries and Malaysia, namely those centred on occupational, mental and sexual health of migrants, and infectious disease, together with health-related issues facing female labour migrants. Whilst there are early signs that Nepal may be moving beyond its predominantly remittance economy, there is no doubt that labour migration to Malaysia and the GCC countries is the reality facing an entire generation of working age Nepalese. The studies identified by the review highlight the need for improved health support, whether through regular health checks in destination countries, more stringent policies and legislation around permissible working conditions or better preparation for migration through more relevant pre-departure training. The findings suggest the urgent need to progressive policy changes, both in Nepal and destination countries, to better protect the health of labour migrants and improve their access to essential health services and acceptable working conditions.

Patient and public involvement This review was conducted as a part of a project to develop a culturally relevant intervention to support the health and wellbeing of Nepalese migrant workers in GCC countries. Migrants workers were involved throughout the project duration, including the formulation of research question for this systematic review.

Authors Contribution: PP and JC designed and supervised the study. PP wrote the review protocol, conducted the literature search, and wrote the final draft of the manuscript. SW and KK screened the articles, extracted the data, carried out quality assessment and contributed to the initial drafts. PP, JC and AM obtained funding for the study. JC, AM and PS reviewed and edited the manuscript. All authors read and approved the final manuscript.

Funding: This review was funded from Research England's institutional allocation from the Global Challenges Research Fund (Reference Number G2626).

Conflict of Interest: None declared.

Data Availability Statement: All relevant data are provided in the manuscript or uploaded as supplementary information.

For peer review only

For peer review only - http://bmjopen.bmj.com/site/about/guidelines.xhtml

References

1. UN. The sustainable development goals report 2019. 2019 ed. New York: United Nations, 2019.
2. IOM. World migration report 2020: International Organization for Migration, 2019.
3. MLESS. Nepal labour migration report 2020. Kathmandu, Nepal: Ministry of Labour Emplyment and Social Security, 2020.
4. NDHS. Nepal Demographic and Health Survey 2016. Kathmandu: Ministry of Health and Population (MoHP) Nepal and ICF International Inc 2017.
5. Joshi S, Simkhada P, Prescott GJ. Health problems of Nepalese migrants working in three Gulf countries. *BMC Int Health Hum Rights* 2011;11(3)
6. Mucci N, Traversini V, Giorgi G, et al. Migrant workers and physical health: An umbrella review. *Sustainability* 2019;11(1):232.
7. Simkhada P, Van Teijlingen E, Gurung M, et al. A survey of health problems of Nepalese female migrants workers in the Middle-East and Malaysia. *BMC Int Health Hum Rights* 2018;18(4)
8. Liberati A, Altman DG, Tetzlaff J, et al. The PRISMA statement for reporting systematic reviews and meta-analyses of studies that evaluate health care interventions: explanation and elaboration. *Journal of clinical epidemiology* 2009;62(10):e1-e34.
9. JBI. The Joanna Briggs Institute critical appraisal tools for use in JBI systematic reviews: Checklist for systematic reviews and research syntheses, 2017.
10. Farsani SF, Brodovicz K, Soleymanlou N, et al. Incidence and prevalence of diabetic ketoacidosis (DKA) among adults with type 1 diabetes mellitus (T1D): a systematic literature review. *BMJ open* 2017;7(7):e016587.
11. Evans C, Tweheyo R, McGarry J, et al. Crossing cultural divides: A qualitative systematic review of factors influencing the provision of healthcare related to female genital mutilation from the perspective of health professionals. *PloS One* 2019;14(3):e0211829.
12. Abu-Madi MA, Behnke JM, Boughattas S, et al. Helminth infections among long-term-residents and settled immigrants in Qatar in the decade from 2005 to 2014: temporal trends and varying prevalence among subjects from different regional origins. *Parasites & Vectors* 2016;9(1):153.
13. Abu-Madi MA, Behnke JM, Ismail A, et al. Assessing the burden of intestinal parasites affecting newly arrived immigrants in Qatar. *Parasites and Vectors* 2016;9(619)
14. Al-Marri M. Pattern of mycobacterial resistance to four anti-tuberculosis drugs in pulmonary tuberculosis patients in the state of Qatar after the implementation of DOTS and a limited expatriate screening programme. *The Int J of TB and Lung Disease* 2001;5(12):1116-21.
15. Al-Thani H, El-Menyar A, Consunji R, et al. Epidemiology of occupational injuries by nationality in Qatar: evidence for focused occupational safety programmes. *Injury* 2015;46(9):1806-13.
16. Humphrey JM, Ranbhise S, Ibrahim E, et al. Multiplex polymerase chain reaction for detection of gastrointestinal pathogens in migrant workers in Qatar. *American J of Trop Med and Hygiene* 2016;95(6):1330-37.
17. Ibrahim AS, Alkhal A, Jacob J, et al. Hepatitis E in Qatar imported by expatriate workers from Nepal: Epidemiological characteristics and clinical manifestations. *J of Med Virology* 2009;81(6):1047-51.
18. Imam YZ, Ahmedullah HS, Akhtar N, et al. Adult tuberculous meningitis in Qatar: a descriptive retrospective study from its referral center. *European Neurology* 2015;73(1-2):90-97.

For peer review only - http://bmjopen.bmj.com/site/about/guidelines.xhtml

19. Kavarodi AM, Thomas M, Kannampilly J. Prevalence of oral pre-malignant lesions and its risk factors in an Indian subcontinent low income migrant group in Qatar. *Asian Pac J Cancer Prev* 2014;15(10):4325-9.
20. Latifi R, El-Menyar A, Al-Thani H, et al. Traffic-related pedestrian injuries amongst expatriate workers in Qatar: a need for cross-cultural injury prevention programme. *Int J of Injury Control and Safety Promotion* 2015;22(2):136-42.
21. Khaled SM, Gray R. Depression in migrant workers and nationals of Qatar: An exploratory cross-cultural study. *International Journal of Social Psychiatry* 2019;65(5):354-67.
22. Irfan FB, Bhutta ZA, Castren M, et al. Epidemiology and outcomes of out-of-hospital cardiac arrest in Qatar: A nationwide observational study. *International Journal of Cardiology* 2016;223:1007-13.
23. Abu-Madi MA, Behnke JM, Ismail A, et al. Comparison of intestinal parasitic infection in newly arrived and resident workers in Qatar. *Parasites & Vectors* 2011;4(1):211.
24. Chan B, Amal RN, Noor Hayati M, et al. Seroprevalence of toxoplasmosis among migrant workers from different Asian countries working in Malaysia. *Southeast Asian J of Trop Med and Public Health* 2008;39(1):9.
25. Min NN, Vasudevan SK, Jasman AA, et al. Work-related ocular injuries in Johor Bahru, Malaysia. *International Eye Science[Article]* 2016;16(3):416-22.
26. Noordin R, Mohd Zain SN, Yunus MH, et al. Seroprevalence of lymphatic filariasis among migrant workers in Peninsular Malaysia. *Transactions of The Royal Society of Trop Med and Hygiene* 2017;111(8):370-72.
27. Sahimin N, Lim YA, Ariffin F, et al. Socio-demographic determinants of Toxoplasma gondii seroprevalence in migrant workers of Peninsular Malaysia. *Parasites and Vectors* 2017;10(1):238.
28. Woh PY, Thong KL, Behnke JM, et al. Characterization of nontyphoidal Salmonella isolates from asymptomatic migrant food handlers in Peninsular Malaysia. *J Food Protection* 2017;80(8):1378-83.
29. Sahimin N, Lim YA, Noordin R, et al. Epidemiology and immunodiagnostics of Strongyloides stercoralis infections among migrant workers in Malaysia. *Asian Pacific Journal of Tropical Medicine* 2019;12(6):250.
30. Woh PY, Thong KL, Behnke JM, et al. Evaluation of basic knowledge on food safety and food handling practices amongst migrant food handlers in Peninsular Malaysia. *Food Control* 2016;70:64-73.
31. Sahimin N, Douadi B, Lim ALY, et al. Distribution of Giardia duodenalis (Assemblages A and B) and Cryptosporidium parvum amongst migrant workers in Peninsular Malaysia. *Acta Tropica* 2018;182:178-84.
32. Adhikary P, Sheppard ZA, Keen S, et al. Risky work: Accidents among Nepalese migrant workers in Malaysia, Qatar and Saudi Arabia. *Health Prospect* 2017;16(2):3-10.
33. Adhikary P, Sheppard ZA, Keen S, et al. Health and well-being of Nepalese migrant workers abroad. *Int J of Migration, Health and Social Care* 2018;14(1):96-105.
34. Joshi S, Prescott GJ, Simkhada P, et al. Knowledge and risk perceptions about HIV/AIDS among Nepalese Migrants in Gulf Countries: a cross-sectional study. *Health Science Journal* 2014;8(3):350-60.
35. Pradhan B, Kjellstrom T, Atar D, et al. Heat stress impacts on cardiac mortality in Nepali migrant workers in Qatar. *Cardiology* 2019;143(1):37-48.
36. Adhikary P, Keen S, Van Teijlingen E. Workplace accidents among Nepali male workers in the Middle East and Malaysia: A qualitative study. *Journal of immigrant and minority health* 2019;21(5):1115-22.
37. Dhakal N, Shah D. SAT-136 Chronic kidney disease in migrant workers in Nepal. *Kidney International Reports* 2020;5(3):S58.

For peer review only - http://bmjopen.bmj.com/site/about/guidelines.xhtml

38. Regmi PR, Aryal N, Van Teijlingen E, et al. Nepali migrant workers and the need for pre-departure training on mental health: a qualitative study. *Journal of Immigrant and Minority Health* 2019:1-9.
39. Alswaidi F, Memish Z, Al Hakeem R, et al. Saudi Arabian expatriate worker fitness-screening programme: a review of 14 years of data. *Eastern Medit Health J* 2013;19(7):664-70.
40. Chattu VK, Mohammad A. Tuberculosis an important global health issue in this era-a cross sectional study of epidemiology of TB among South Asian workers in Saudi Arabia. *Indian J Public Health* 2013;4:278.
41. Dafalla AIA, Almuhairi SASO, AlHosani MHJ, et al. Intestinal parasitic infections among expatriate workers in various occupations in Sharjah, United Arab Emirates. *Rev Inst Med Trop Sao Paulo* 2017;59(e82)
42. Al-Awadhi M, Iqbal J, Ahmad S. Cysticercosis, a Potential Public Health Concern in Kuwait: A New Diagnostic Method to Screen Taenia solium Taeniasis Carriers in the Expatriate Population. *Medical Principles and Practice* 2019 doi: DOI: 10.1159/000504625
43. Pattisson P. Majority of Nepal migrant deaths "should be treated as murder", Global development. The Guardian [Internet]. The Guardian. 2014 . https://www.theguardian.com/global-development/2014/may/20/nepal-migrant-deaths-treated-murder (21 October, 2019, date last accessed). 2014
44. Yang H, Gao J, Wang T, et al. Association between adverse mental health and an unhealthy lifestyle in rural-to-urban migrant workers in Shanghai. *J Formosan Medl Assoc* 2017;116(2):90-98.
45. JPAN. Migrant worker and mental health in Nepal. *J Psych Assoc of Nepal* 2014;1(1)
46. Poudel A. Mental health of migrant workers is a pressing issue, but it has been ignored [Internet]. https://kathmandupost.com/valley/2019/05/18/mental-health-of-migrant-workers-is-a-pressing-issue-but-it-has-been-ignored (21 Octomber, 2019, date last accessed), 2019.
47. Chapagai M, Pant S, Tulachan P, et al. Psychiatric morbidity among repatriated Nepalese foreign labor migrants-a hospital based study. *J Instit Med* 2017;41(1)
48. Maselko J. Social epidemiology and global mental health: expanding the evidence from high-income to low-and middle-income countries. *Current Epid Reports* 2017;4(2):166-73.
49. Weston G, Zilanawala A, Webb E, et al. Long work hours, weekend working and depressive symptoms in men and women: findings from a UK population-based study. *J Epid Com Health* 2019;73(5):465-74.
50. Donini A. Social suffering and structural violence: Nepali workers in Qatar. *Int Dev Policy* 2019:178-99. http://journals.openedition.org/poldev/3077 (24 October, 2019, date last accessed).
51. Murphy JM, Olivier DC, Monson RR, et al. Depression and anxiety in relation to social status: A prospective epidemiologic study. *Archives of General Psychiatry* 1991;48(3):223-29.
52. Lao CK, Chan YM, Tong HHY, et al. Underdiagnosis of depression in an economically deprived population in Macao, China. *Asia-Pacific Psychiatry* 2016;8(1):70-79.
53. Pulkki-Råback L, Ahola K, Elovainio M, et al. Socio-economic position and mental disorders in a working-age Finnish population: the health 2000 study. *The European J of Public Health* 2011;22(3):327-32.
54. Pocock NS, Chan Z, Loganathan T, et al. Moving towards culturally competent health systems for migrants? Applying systems thinking in a qualitative study in Malaysia and Thailand. *PloS One* 2020;15(4):e0231154.

For peer review only - http://bmjopen.bmj.com/site/about/guidelines.xhtml

55. Poudel KC, Jimba M, Okumura J, et al. Migrants' risky sexual behaviours in India and at home in far western Nepal. *J Trop Med Hyg* 2004;9(8):897-903.
56. Bam K, Thapa R, Newman MS, et al. Sexual behavior and condom use among seasonal Dalit migrant laborers to India from Far West, Nepal: a qualitative study. *PLoS One* 2013;8(9):e74903.
57. Simkhada PP, Regmi PR, Van Teijlingen E, et al. Identifying the gaps in Nepalese migrant workers' health and well-being: a review of the literature. *J of Travel Med* 2017;24(4)
58. Aryal N, Regmi PR, van Teijlingen E, et al. Adolescents left behind by migrant workers: a call for community-based mental health interventions in Nepal. *WHO South-East Asia J of Public Health* 2019;8(1):38-41.
59. Aryal N, Regmi P, Teijlingen E, et al. Knowing is not enough: migrant workers' spouses vulnerability to HIV. *SAARC J TB, Lung Diseases and HIV/AIDS* 2016;13(1):9-15.
60. Thapa S, Bista N, Hannes K, et al. Vulnerability of wives of Nepalese labor migrants to HIV infection: integrating quantitative and qualitative evidence. *Women and Health* 2016;56(7):745-66.
61. Urmi AZ, Leung DT, Wilkinson V, et al. Profile of an HIV testing and counseling unit in Bangladesh: majority of new diagnoses among returning migrant workers and spouses. *PloS One* 2015;10(10):e0141483.
62. Ning C, Jiang J, Ye L, et al. Comparison of three intervention models for promoting circumcision among migrant workers in western China to reduce local sexual transmission of HIV. *PloS one* 2013;8(9):e76107.
63. Seddon D, Adhikari J, Gurung G. Foreign labor migration and the remittance economy of Nepal. *Critical Asian Studies* 2002;34(1):19-40.
64. Bhandari P. Relative deprivation and migration in an agricultural setting of Nepal. *Popn and Envt* 2004;25(5):475-99.
65. Sadarangani SP, Lim PL, Vasoo S. Infectious diseases and migrant worker health in Singapore: a receiving country's perspective. *J of Travel Med* 2017;24(4)
66. Castelli F, Sulis G. Migration and infectious diseases. *Clinical Microb Infect* 2017;23(5):283-89.
67. Colombini M, Mayhew SH, Hawkins B, et al. Agenda setting and framing of gender-based violence in Nepal: how it became a health issue. *Health Policy and Planning* 2015;31(4):493-503.
68. Gioli G, Maharajan A, M G. Neither heroines nor victims: Women migrant workers and changing family and community relations in Nepal [Internet]. 2017. https://www.refworld.org/pdfid/5a1bf0374.pdf (21 October, 2019, date last accessed).2017.
69. TheWorldBank. Migration and remittances factbook 2016 advanced edition [Internet]. 2016. https://siteresources.worldbank.org/INTPROSPECTS/Resources/334934-1199807908806/4549025-1450455807487/Factbookpart1.pdf (21 October, 2019, date last accessed) 2016
70. WOREC. Women and migration. [Internet] 2012. https://issuu.com/worecnepal /docs/migration-and-women (24 Nov, 2019, date last accessed)2012.
71. Pyakurel UP. Restrictive labour migration policy on Nepalese women and consequences. *Sociology and Anthropology* 2018;6(8):650-56.
72. Campbell M, McKenzie JE, Sowden A, et al. Synthesis without meta-analysis (SWiM) in systematic reviews: reporting guideline. *bmj* 2020;368.

For peer review only - http://bmjopen.bmj.com/site/about/guidelines.xhtml

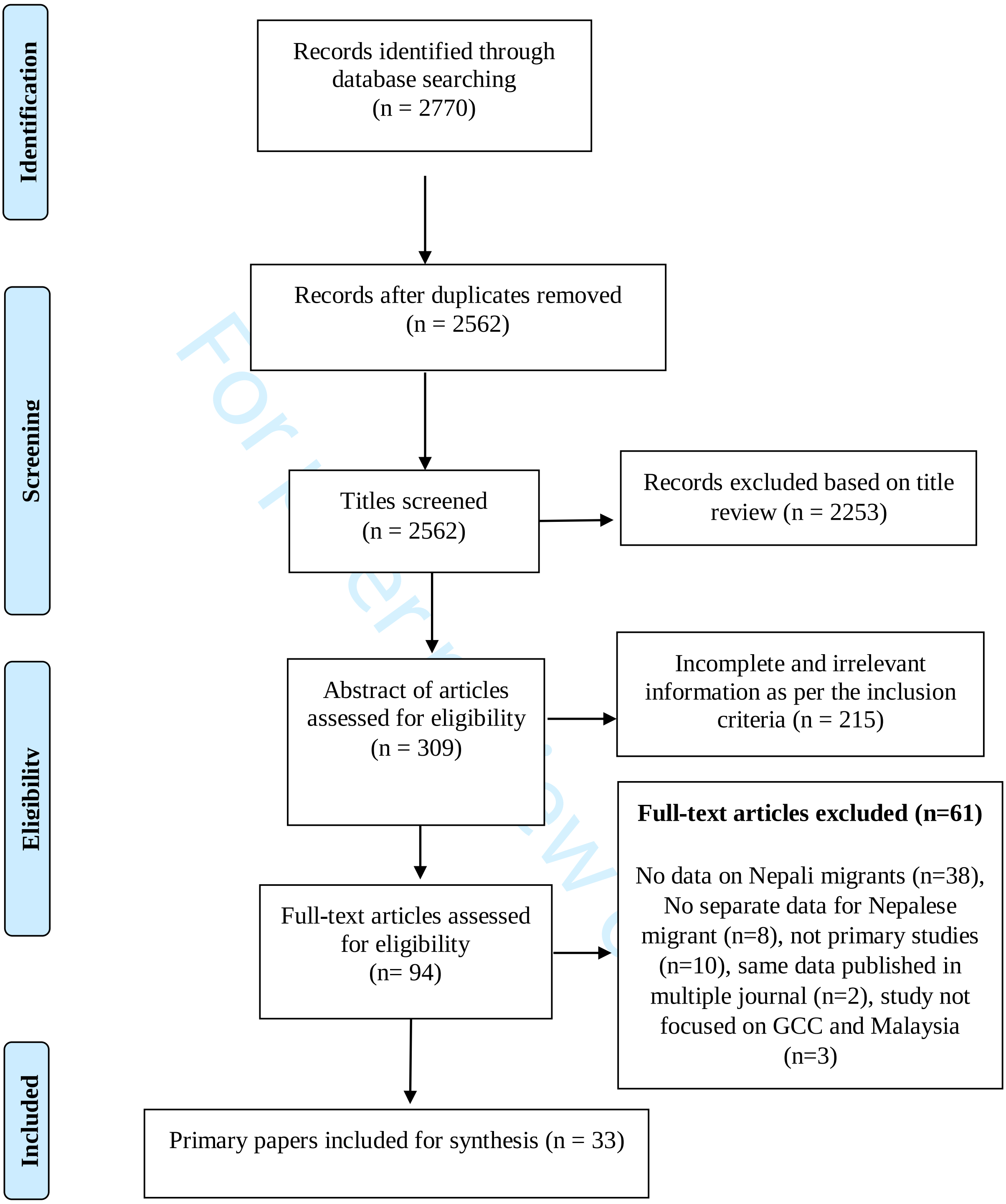

Figure 1: PRISMA Flow Diagram of Study Selection

Appendix 1 Keywords used for search in MEDLINE

1. Migration {Including Related Terms}
2. Migrant {Including Related Terms}
3. Emigrant {Including Related Terms}
4. Immigrant{Including Related Terms}
5. Expatriate {Including Related Terms}
6. Foreign worker {Including Related Terms}
7. Labor migration {Including Related Terms}
8. Left-behind {Including Related Terms}
9. Migrant families {Including Related Terms}
10. Or/1-9
11. Nepal {Including Related Terms}
12. Nepalese {Including Related Terms}
13. Nepali {Including Related Terms}
14. UAE or United Araba Emirates {Including Related Terms}
15. GCC or Gulf Cooperating council {Including Related Terms}
16. Middle East {Including Related Terms}
17. Bahrain {Including Related Terms}
18. Saudi Arabia {Including Related Terms}
19. Oman {Including Related Terms}
20. Qatar {Including Related Terms}
21. Kuwait {Including Related Terms}
22. Malaysia {Including Related Terms}
23. Or/11-22
24. 10 AND 23

For peer review only

For peer review only

Appendix 2:

Quality Assessment of Quantitative Studies (Prevalence Surveys)

Reference	Was the sample frame appropriate to address the target population?	Were study participants sampled in an appropriate way?	Was the sample size adequate?	Were the study subjects and the setting described in detail?	Was the data analysis conducted with sufficient coverage of the identified sample?	Were valid methods used for the identification of the condition?	Was the condition measured in a standard, reliable way for all participants?	Was there appropriate statistical analysis?	Was the response rate adequate, and if not, was the low response rate managed appropriately?	Scores	Overall quality
Overview N-28 studies	Yes - 27 (96.5%)	Yes - 20 (71.4%)	Yes - 17 (60.7%)	Yes – 25 (89.3%)	Yes – 26 (92.9%)	Yes – 26 (92.9%)	Yes – 25 (89.3%)	Yes – 25 (89.3%)	Yes – 15 (53.6%)		H- 15 (53.6%)
	Unclear - 1 (3.5%)	Unclear- 7 (25.0%)	Unclear– 11 (39.3%)	Unclear – 3 (10.7%)	Unclear – 2 (7.1%)	Unclear – 2 (7.1%)	Unclear – 3 (10.7%)	Unclear – 3 (10.7%)	Unclear – 13 (46.4%)		M- 13 (46.4%)
	No - 0	No - 1 (3.6%)	No - 0	No – 0	No - 0	No - 0	No - 0	No - 0	No - 0		
Abu-Madi et al, 2016a	Yes	Yes	Yes	Yes	Yes	Yes	Yes	Yes	Yes	9	High
Abu-Madi et al, 2016b	Yes	Yes	Yes	Unclear	Yes	Yes	Yes	Unclear	Yes	7	Moderate
Abu-Madi et al, 2011	Yes	Yes	Unclear	Yes	Yes	Yes	Yes	Yes	Yes	8	High
Al-Awadhi et al, 2019	Yes	Yes	Yes	Yes	Yes	Yes	Yes	Yes	Unclear	8	High
Al-Marri et al, 2001	Yes	Yes	Yes	Yes	Yes	Yes	Yes	Unclear	Yes	8	High
Alswaidi et al, 2013	Yes	Yes	Yes	Yes	Yes	Yes	Yes	Yes	Yes	9	High
Al-Thani et al, 2015	Yes	Unclear	Yes	Yes	Yes	Yes	Unclear	Yes	Yes	7	Moderate
Chan et al, 2008	Unclear	Yes	Yes	Yes	Yes	Yes	Yes	Yes	Yes	8	High
Chattu et al, 2013	Yes	Yes	Unclear	Yes	Unclear	Yes	Unclear	Yes	Unclear	5	Moderate
Dafalla et al, 2017	Yes	Unclear	Yes	Yes	Yes	Yes	Yes	Yes	Unclear	7	Moderate
Dhakal et al, 2020	Yes	Yes	Unclear	Yes	Yes	Unclear	Yes	Yes	Unclear	6	Moderate
Humphery et al, 2016	Yes	Unclear	Unclear	Yes	Yes	Yes	Yes	Yes	Unclear	6	Moderate
Ibrahim et al, 2009	Yes	Yes	Unclear	Yes	Unclear	Yes	Yes	Yes	Unclear	6	Moderate
Imam et al, 2015	Yes	Yes	Unclear	Yes	Yes	Yes	Yes	Yes	Yes	8	High
Irfan et al, 2016	Yes	Yes	Yes	Yes	Yes	Yes	Yes	Yes	Unclear	8	High

For peer review only

Reference	Was the sample frame appropriate to address the target population?	Were study participants sampled in an appropriate way?	Was the sample size adequate?	Were the study subjects and the setting described in detail?	Was the data analysis conducted with sufficient cover age of the identified sample?	Were valid methods used for the identification of the condition?	Was the condition measured in a standard, reliable way for all participants?	Was there appropriate statistical analysis?	Was the response rate adequate, and if not, was the low response rate managed appropriately?	Scores	Overall quality
Joshi et al, 2011	Yes	Yes	Yes	Yes	Yes	Yes	Yes	Yes	Yes	9	High
Joshi et al, 2014	Yes	No	Yes	Yes	Yes	Unclear	Yes	Unclear	Yes	7	Moderate
Kavarodi et al, 2014	Yes	Yes	Yes	Yes	Yes	Yes	Yes	Yes	Yes	9	High
Latifi et al, 2015	Yes	Yes	Yes	Unclear	Yes	Yes	Yes	Yes	Yes	8	High
Min, 2016	Yes	Yes	Unclear	Yes	Yes	Yes	Yes	Yes	Yes	8	High
Noordin et al, 2017	Yes	Yes	Unclear	Unclear	Yes	Yes	Yes	No	Unclear	5	Moderate
Pradhan et al, 2019	Yes	Unclear	Unclear	Yes	Yes	Yes	Unclear	Yes	Unclear	5	Moderate
Sahimin et al, 2019	Yes	Yes	Yes	Yes	Yes	Yes	Yes	Yes	Unclear	8	High
Sahimin et al, 2018	Yes	Yes	Yes	Yes	Yes	Yes	Yes	Yes	Yes	9	High
Sahimin et al, 2017	Yes	Yes	Yes	Yes	Yes	Yes	Yes	Yes	Yes	9	High
Simkhada et al, 2017	Yes	Unclear	Unclear	Yes	Yes	Yes	Yes	Yes	Unclear	6	Moderate
Woh et al, 2016	Yes	Unclear	Unclear	Yes	Yes	Yes	Yes	Yes	Unclear	6	Moderate
Woh et al, 2017	Yes	Unclear	Unclear	Yes	Yes	Yes	Yes	Yes	Yes	7	Moderate

For peer review only

Quality Assessment of Quantitative Studies (Analytical Cross-sectional Surveys)

Reference (n=3 studies)	Were the criteria for inclusion in the sample clearly defined?	Were the study subjects and the setting described in detail?	Was the exposure measured in a valid and reliable way?	Were objective, standard criteria used for measurement of the condition?	Were confounding factors identified?	Were strategies to deal with confounding factors stated?	Were the outcomes measured in a valid and reliable way?	Was appropriate statistical analysis used?	Score	Overall Quality
Adhikary et al, 2017	Yes	Yes	Yes	Yes	No	No	Yes	Yes	6	Moderate
Adhikary et al, 2018	Yes	Yes	Yes	Yes	No	No	Yes	Yes	6	Moderate
Khaled and Gray, 2019	Yes	Yes	Yes	Yes	No	No	Yes	Yes	6	Moderate

Quality Assessment of the Qualitative Studies

Reference (n=2 studies)	Is there congruity between the stated philosophical perspective and the research methodology?	Is there congruity between the research methodology and the research question or objectives?	Is there congruity between the research methodology and the methods used to collect data?	Is there congruity between the research methodology and the representation and analysis of data?	Is there congruity between the research methodology and the interpretation of results?	Is there a statement locating the researcher culturally or theoretically?	Is the influence of the researcher on the research, and vice- versa, addressed?	Are participants, and their voices, adequately represented?	Is the research ethical according to current criteria or, for recent studies, and is there evidence of ethical approval by an appropriate body?	Do the conclusions drawn in the research report flow from the analysis, or interpretation, of the data?	Score	Overall Quality
Adhikary et al, 2019	Yes	Yes	Yes	Yes	Yes	Unclear	Unclear	Yes	Yes	Yes	8	High
Regmi et al, 2019	Yes	Yes	Yes	Unclear	Yes	No	Unclear	Yes	Yes	Yes	7	Moderate

PRISMA 2009 Checklist

Section/topic	#	Checklist item	Reported on page #
**TITLE**			
Title	1	Identify the report as a systematic review, meta-analysis, or both.	1
**ABSTRACT**			
Structured summary	2	Provide a structured summary including, as applicable: background; objectives; data sources; study eligibility criteria, participants, and interventions; study appraisal and synthesis methods; results; limitations; conclusions and implications of key findings; systematic review registration number.	2
**INTRODUCTION**			
Rationale	3	Describe the rationale for the review in the context of what is already known.	4
Objectives	4	Provide an explicit statement of questions being addressed with reference to participants, interventions, comparisons, outcomes, and study design (PICOS).	4
**METHODS**			
Protocol and registration	5	Indicate if a review protocol exists, if and where it can be accessed (e.g., Web address), and, if available, provide registration information including registration number.	5
Eligibility criteria	6	Specify study characteristics (e.g., PICOS, length of follow-up) and report characteristics (e.g., years considered, language, publication status) used as criteria for eligibility, giving rationale.	5
Information sources	7	Describe all information sources (e.g., databases with dates of coverage, contact with study authors to identify additional studies) in the search and date last searched.	5
Search	8	Present full electronic search strategy for at least one database, including any limits used, such that it could be repeated.	Appendix 1
Study selection	9	State the process for selecting studies (i.e., screening, eligibility, included in systematic review, and, if applicable, included in the meta-analysis).	5,6
Data collection process	10	Describe method of data extraction from reports (e.g., piloted forms, independently, in duplicate) and any processes for obtaining and confirming data from investigators.	6
Data items	11	List and define all variables for which data were sought (e.g., PICOS, funding sources) and any assumptions and simplifications made.	6
Risk of bias in individual studies	12	Describe methods used for assessing risk of bias of individual studies (including specification of whether this was done at the study or outcome level), and how this information is to be used in any data synthesis.	6,7
Summary measures	13	State the principal summary measures (e.g., risk ratio, difference in means).	6
Synthesis of results	14	Describe the methods of handling data and combining results of studies, if done, including measures of consistency (e.g., I^2) for each meta-analysis.	6

For peer review only - http://bmjopen.bmj.com/site/about/guidelines.xhtml

PRISMA 2009 Checklist

Section/topic	#	Checklist item	Reported on page #
Risk of bias across studies	15	Specify any assessment of risk of bias that may affect the cumulative evidence (e.g., publication bias, selective reporting within studies).	6,7
Additional analyses	16	Describe methods of additional analyses (e.g., sensitivity or subgroup analyses, meta-regression), if done, indicating which were pre-specified.	NA
**RESULTS**			
Study selection	17	Give numbers of studies screened, assessed for eligibility, and included in the review, with reasons for exclusions at each stage, ideally with a flow diagram.	7
Study characteristics	18	For each study, present characteristics for which data were extracted (e.g., study size, PICOS, follow-up period) and provide the citations.	9-20
Risk of bias within studies	19	Present data on risk of bias of each study and, if available, any outcome level assessment (see item 12).	Appendix 2
Results of individual studies	20	For all outcomes considered (benefits or harms), present, for each study: (a) simple summary data for each intervention group (b) effect estimates and confidence intervals, ideally with a forest plot.	Table 1
Synthesis of results	21	Present results of each meta-analysis done, including confidence intervals and measures of consistency.	NA
Risk of bias across studies	22	Present results of any assessment of risk of bias across studies (see Item 15).	Appendix 2
Additional analysis	23	Give results of additional analyses, if done (e.g., sensitivity or subgroup analyses, meta-regression [see Item 16]).	NA
**DISCUSSION**			
Summary of evidence	24	Summarize the main findings including the strength of evidence for each main outcome; consider their relevance to key groups (e.g., healthcare providers, users, and policy makers).	21
Limitations	25	Discuss limitations at study and outcome level (e.g., risk of bias), and at review-level (e.g., incomplete retrieval of identified research, reporting bias).	24
Conclusions	26	Provide a general interpretation of the results in the context of other evidence, and implications for future research.	25
**FUNDING**			
Funding	27	Describe sources of funding for the systematic review and other support (e.g., supply of data); role of funders for the systematic review.	25

From: Moher D, Liberati A, Tetzlaff J, Altman DG, The PRISMA Group (2009). Preferred Reporting Items for Systematic Reviews and Meta-Analyses: The PRISMA Statement. PLoS Med 6(7): e1000097. doi:10.1371/journal.pmed1000097

For peer review only - http://bmjopen.bmj.com/site/about/guidelines.xhtml
